# Meta-Analysis of Autoimmune Regulator-Regulated Genes in Human and Murine Models: A Novel Human Model Provides Insights on the Role of Autoimmune Regulator in Regulating STAT1 and STAT1-Regulated Genes

**DOI:** 10.3389/fimmu.2018.01380

**Published:** 2018-06-28

**Authors:** Thomas R. J. Lovewell, Andrew J. G. McDonagh, Andrew G. Messenger, Mimoun Azzouz, Rachid Tazi-Ahnini

**Affiliations:** ^1^Department of Infection, Immunity and Cardiovascular Disease, The Medical School, University of Sheffield, Sheffield, United Kingdom; ^2^Department of Dermatology, Royal Hallamshire Hospital, Sheffield, United Kingdom; ^3^Department of Neuroscience, The Medical School, University of Sheffield, Sheffield, United Kingdom

**Keywords:** Aire gene, STAT1, auitoimmune polyglandular syndrome 1, tissue-restricted antigens, TEC1 A3 cells

## Abstract

Autoimmune regulator (AIRE) regulates promiscuous expression of tissue-restricted antigens in medullary epithelial cells (mTEC) of the thymus. To understand the diverse effects of AIRE, it is crucial to elucidate the molecular mechanisms underlying the process of AIRE-regulated gene expression. In this study, we generated a recombinant AIRE expression variant of the TEC 1A3 human cell line, TEC 1A3 AIRE^hi^, to determine genes targeted by AIRE, and using microarray analysis, we identified 482 genes showing significant differential expression (*P* < 0.05; false discovery rate <5%), with 353 upregulated and 129 downregulated by AIRE expression. Microarray data were validated by quantitative PCR, confirming the differential expression of 12 known AIRE-regulated genes. Comparison of AIRE-dependent differential expression in our cell line model with murine datasets identified 447 conserved genes with a number of transcription regulatory interactions, forming several key nodes, including STAT1, which had over 30 interactions with other AIRE-regulated genes. As STAT1 mutations cause dominant chronic mucocutaneous candidiasis and decreased STAT1 levels in monocytes of autoimmune polyglandular syndrome 1 (APS-1) patients, it was important to further characterize AIRE–STAT1 interactions. TEC 1A3AIRE^hi^ were treated with the STAT1 phosphorylation inhibitors fludarabine and LLL3 showed that phosphorylated STAT1 (p-STAT1) was not responsible for any of the observed differential expression. Moreover, treatment of TEC 1A3 AIRE^hi^ with STAT1 shRNA did not induce any significant variation in the expression of unphosphorylated STAT1 (U-STAT1) downstream genes, suggesting that these genes were directly regulated by AIRE but not *via* U-STAT1. The novel model system we have developed provides potential opportunities for further analysis of the pathogenesis of (APS-1) and the wider roles of the AIRE gene.

## Introduction

Autoimmune polyglandular syndrome 1 (APS-1) is a rare monogenic disorder, with characteristic features of hypoparathyroidism, autoimmune Addison’s disease, chronic mucocutaneous candidiasis (CMC), and a wide range of other autoimmune disorders found at a higher incidence than in the general population (1, 2). APS-1 is caused by mutations in the autoimmune regulator (AIRE) gene (3, 4), a transcription factor that regulates promiscuous expression of tissue-restricted antigens in the thymus and plays a role in central tolerance mechanisms (5). AIRE has been found to bind to DNA in a sequence-specific manner (6) and to bind to histone H3. However, the interaction with this histone is inhibited by methylation of lysine 4 (K4) or chemical modification of other residues in close proximity to K4. These modifications are typically associated with chromatin that is activated for transcription (7–9). AIRE also interacts with several other proteins (10) and activates promiscuous gene expression of TSA’s by releasing blocked RNA polymerase II (RNA Pol II), suggesting its involvement in transcriptional elongation rather than transcriptional initiation (11). AIRE/Aire interacts with transcriptional co-activator CREB-binding protein (CBP), binds to local histone marks H3K4, and recruits positive transcription elongation factor b (P-TEFb) by interacting with C-type cyclin (CycT1) (7, 12, 13). P-TEFb phosphorylates NELF and DSIF causing dislodement of the former and converting the latter into an elongation factor. This change enables phosphorylated RNA Pol II to perform productive elongation and results in the activation of gene expression, which proceeds until termination (14). DNA-dependent protein kinase (DNA-PK) phosphorylates AIRE/Aire and influences its transactivation function (15). Sufficient levels of RNA Pol II, and unmodified H3K4, and DNA-PK proteins ensure that AIRE/Aire is recruited and lead to the expression of TSA’s and their presentation to T-cells by MHC class II determinants. In addition, the protein inhibitor of activated STAT1 interacts with AIRE to regulate target genes (16). AIRE is a major regulator of thousands of TSA genes expressed in mTECs and affects their transcription in a stochastic but ordered manner (17). Indeed, AIRE is selectively expressed in approximately 50% of MHC class II^hi^ mTECs, and a small subset (1–3%) of the total number of mTECs expresses each specific TSA (10, 18). Among the most interesting AIRE-regulated genes are those encoding autoantigens for which TSA’s have been detected in APS-1 patients. In fact, serum from APS-1 patients has been found to have high titers of autoantibodies against more than 20 tissue-specific antigens (19). For instance, in APS-1 with hypoparathyroidism, NLR family pyrin domain containing 5 (NLRP5) (20) and calcium-sensing receptor (CaSR) have been identified as parathyroid autoantigens (21). NLRP5 is a cytoplasmic receptor expressed most specifically in the chief cells of the parathyroid gland and in oocytes (22). Autoantibodies against NLRP5 have been detected in almost 50% of APS-1 patients with hypoparathyroidism but were absent in all APS-1 patients without hypoparathyroidism (20). Autoantibodies against steroid 21-hydroxylase (CYP21A2), steroid 17α-hydroxylase (CYP17A1), and cytochrome P450 family 11 subfamily A member 1 (CYP11A1) have been detected in many APS-1 patients with Addison’s disease years before adrenal insufficiency became clinically apparent (23). These autoantigens are also reported to be involved in hypogonadism in APS-1 patients (20, 24). In addition, autoantibodies against prostate antigen transglutaminase 4 (TGM4), a regulator of semen viscosity and promoter of semen coagulation, can be found in male patients with APS-1 (25). T1D occurs in only 1–18% of APS-1 patients but high titer autoantibodies against diabetes-related autoantigens, such as glutamic acid decarboxylase isoform 65 (GAD65), glutamic acid decarboxylase isoform 67 (GAD67), aromatic l-amino acid decarboxylase (AADC), and islet antigen 2 (IA-2) have been detected in 30–66% of APS-1 patients (26, 27). APS-1 patients with tryptophan hydroxylase autoantibodies develop intestinal malabsorption (28). Interstitial lung disease is reported in 6–10% of APS-1 patients in association with autoantibodies to potassium channel-regulating protein (KCNRG) and bactericidal/permeability-increasing fold-containing B1 protein (BPIFB1) (29, 30). APS-1 patients with hypothyroidism show antibodies to thyroglobulin and thyroid peroxidase (31). The transcription factors SRY-box 9 (SOX9) and SRY-Box 10 (SOX10) were reported to be vitiligo autoantigens in APS-1 patients with autoantibodies detected in 15 and 22% of sera, respectively (32). Hedstrand et al. (33) reported an autoantigen related to APS-1 tyrosine hydroxylase, was identified in sera from patients with alopecia areata (AA) with immunoreactivity detected in 44% of the 94 APS-1 patients studied and this reactivity associated with the presence of AA.

Since the advent of *Aire* knockout mice, we have been able to explore the biological effects of Aire at the systemic, tissue, and cellular level and the thymi from these murine models have been used to explore the biochemical properties of Aire, particularly for determining the downstream targets of aire-regulated gene expression (ARGE) (5, 34–39). Cell line models in many respects are more suited for exploring the molecular mechanisms of AIRE/Aire function, as they are not limited by the number of AIRE^+^/Aire^+^ cells in a single tissue, can be easily manipulated by chemical and genetic treatments and critically, are the only available option for the study of AIRE in a model system of human origin (40). Our aim was to determine the human gene targets of ARGE, then, to identify and explore any conserved patterns of expression between our cell line model and murine ARGE data.

## Materials and Methods

### Cell Culture

The TEC 1A3 cell line was originally established from fetal and postnatal human thymus tissue by Toribio’s group in 1994 (41). TEC 1A3 cells (a gift from Dr. Gauchon, Inserm U580, Paris) were maintained in RPMI 1640 medium with l-glutamine (Lonza) supplemented with 10% fetal calf serum (Lonza). Medium for cell line variants were supplemented with either zeocin or hygromycin B, dependent on the cell line variant.

### Generation of TEC 1A3 flp-in Host Cell Line

A TEC 1A3 cell line containing a single FRT (Flp recombinase target site) was generated using the pFRT/*LacZeo* vector following the flp-in system manual (012402 version C, Invitrogen). In brief, this process involved the transfection of TEC 1A3 cells with a pFRT/*LacZeo* vector using FuGENE6 reagent (Promega). Cells were grown in media supplemented with 250 µg/ml zeocin to select for those containing the pFRT/*LacZeo* vector integrated into the genome, with cloning rings used to isolate foci to collect individual cell lines. Southern blotting was used to screen the different cell lines to identify those containing only a single integrant of the pFRT/*LacZeo* vector. Relative expression efficiency from the FRT region for each TEC 1A3/pFRT/*LacZeo* cell line generated was estimated by comparing β-galactosidase activity using the β-gal assay kit (Invitrogen). The cell line with the highest β-galactosidase activity was chosen to be the TEC 1A3 flp-In host cell line for subsequent reactions.

### Generation of Stably Transfected Recombinant AIRE (rAIRE) Expression Cell Lines

Autoimmune regulator cDNA was PCR amplified from a pET31/rAIRE expression vector (a gift from Prof. She, Medical College of Georgia, USA) using the primers AAAAAAGCTAGCCGCCACCATGGCGACGGACGCGGCGCTA and GCTACAGGGCCCTCAATGATGATGATGATGATG.

The amplified cDNA was inserted into the multiple-cloning site of the pcDNA5/FRT vector using the NheI and ApaI restriction sites, generating the pcDNA5/FRT/rAIRE expression vector. Both the TEC 1A3 flp-in host cells and T-REx 293 cells were seeded into 6-well plates to reach 60–70% confluence after 24 h. The cells were co-transfected with 1 µg plasmid DNA (the Flp recombinase expression vector pOG44 with the pcDNA5/FRT/rAIRE expression vector at 20:1 ratio) using 3 µl FuGENE6 reagent (Promega) per transfection. After 24 h, cells were washed with PBS then maintained in fresh media supplemented with hygromycin B at 150 µg/ml to select for cells containing integrated pcDNA5/FRT/rAIRE expression vector (AIRE^hi^ variants).

### Western Blotting

Cell pellets were resuspended in 2× loading buffer [100 mM tris–Cl (pH 6.8), 4% SDS, 20% glycerol, 0.2% bromophenol blue, and 200 mM dithiothreitol] and proteins separated by SDS-PAGE [pH8.8, 10% (37:1) acrylamide, 0.375 M tris–Cl, and 0.1% SDS]. After electrophoresis, gel was transferred to PVDF membrane using iblot system (Invitrogen). Primary antibodies used were 1:1,000 polyclonal goat anti-aire-1 (D-17) IgG (sc-17986, Santa Cruz Biotechnology, Inc.) and 1:500 polyclonal goat anti-actin (I-19) IgG (sc-1616, Santa Cruz Biotechnology, Inc.). The secondary antibody used was 1:2,500 polyclonal rabbit anti-goat IgG conjugated with peroxidase (A5420, Sigma), visualized using EZ-ECL chemiluminescent reagent (Biological Industries). The PhosphoPlus Stat1 (Tyr701) antibody kit was used to detect STAT1 and p-STAT1 (Tyr701) in samples.

### Microarray Analysis

RNA was extracted from cell pellets from three separate cultures of TEC 1A3 AIRE^lo^ cells and three separate cultures of TEC 1A3 AIRE^hi^ cells using the SV Total RNA isolation kit (Promega). The quality and quantity of the RNA from all samples was assessed (2100 bioanalyzer, RNA 6000 Pico LabChip; Agilent, CA, USA, and NanoDrop 1000 spectrophotometer, respectively). Linear amplification of each RNA sample was undertaken following the Eberwine procedure (42) using the GeneChip^®^ 3′ IVT Express Kit (Affymetrix), and checked again for quality and quantity. Fifteen microgram of amplified cRNA from the three TEC 1A3 AIRE^lo^ cells and three TEC 1A3 AIRE^hi^ cells were fragmented and each hybridized individually to a separate Human Genome U133 Plus 2.0 GeneChip (Affymetrix), as specific in manufacturer’s protocols, and subsequently underwent washes. Chips were then stained, scanned, and signal intensities for each transcript produced using GeneChip Operating Software. These data (uploaded to GSE 35244) and data generated from wild-type and rAire^+^ HEK 293 cells [GSE24733 (8)] were analyzed using Genespring GX v11.0 (Agilent Technologies) with the PLIER16 algorithm used to normalize these values, and subsequently analyzed by Student’s t-test [*P* ≤ 0.05 and *P* ≤ 0.05 with Benjamini–Hochberg correction to ensure false discovery rate (FDR) <5%] to determine genes that showed significant differential expression in the AIRE^hi^ (rAire^+^ in HEK) cells compared to AIRE^lo^ (Aire^−^/wild-type in HEK) cells.

### Quantitative and RT-PCR

RNA was extracted from cells using trizol and purified from the trizol-cell lysate using the Direct-Zol miniprep kit (Zymo Research), before being used for cDNA synthesis using the Superscipt III First-Strand Synthesis System (Invitrogen) with random hexamer primers. cDNA synthesis for all three RNA samples from TEC 1A3 AIRE^lo^ and TEC 1A3 AIRE^hi^ cells used for microarray analysis was carried out the same way.

Triplicate QPCR reactions were carried out for each sample using *Power* SYBR^®^ Green PCR (Applied Biosystems) and each of the primer pairs listed in Table S1 in Supplementary Material. Validation QPCR reactions were carried out in 96 well plates using a Stratagene MX3000P QPCR System (Aglient Technologies). Other QPCR reactions were carried out in 384-well plates using an Applied Bioystems 7900HT Real-Time PCR machine. β-Actin expression was tested for each RNA sample on every plate and used to normalize C_t_ values determined for the other genes tested. The ΔΔC_t_ method was used to calculate the change in relative expression for each gene between AIRE^lo^ and AIRE^hi^ cells, or between untreated and treated AIRE^hi^ cells.

RT-PCR was carried out using AmpliTaq Gold 360 (Applied Biosystems) under standard reaction conditions using 1 µl human adult thymus cDNA (c1234264, AMS biotechnology) and primers at a final concentration of 0.5 µM. The standard PCR program from the AmpliTaq Gold 360 product manual was used, with an annealing temperature of 60°C and 38 cycles. Gene-specific primer sequences are given in Table S3 in Supplementary Material.

### Generation of Conserved Gene List

Genespring GX v11.0 (Agilent Technologies) was again used to analyze data generated on medullary thymic epithelial cells from wild-type and Aire knockout mice of the C57BL/6, BALB/c, and NOD strains [GSE8564 (38) and GSE12073 (39)], with the PLIER16 algorithm used to normalize raw values. Each dataset is the “differential expression dataset” generated by comparing gene expression between normalized data from WT samples and normalized data from Aire KO samples. Two “differential expression datasets” were generated from NOD mice, together with one from each of B6 and Balb/c.

These datasets, along with our normalized TEC1A3 cell line dataset (GSE35244), were then analyzed by Student’s *t*-test (*P* ≤ 0.05) to determine genes that showed significant differential expression in the AIRE^hi^ (wild-type mice) cells compared to AIRE^lo^ (knockout mice) cells. Genespring GX was used to compare individually each analyzed murine dataset with our analyzed TEC 1A3 data set to determine gene conservation across two data sets. Individual conserved gene lists were exported, compiled, and filtered to show only genes with differential expression reported for the human dataset and at least three of the four murine datasets, with each reported gene showing a fold-change ≥ 1.2 for probes from at least three different datasets.

### Functional Analysis of AIRE-Regulated Genes

The lists of AIRE-regulated genes from both our TEC 1A3 data set and from the conserved gene list were analyzed by DAVID^1^ (43) and the Edge-Express DB element of Fantom 4^2^ (44).

### Inhibition and Gene Knockdown

The STAT1 phosphorylation inhibitors fludarabine phosphate (Sigma) and LLL3, a kind gift from Dr. Pui-Kai Li, College of Pharmacy, Ohio State University, were dissolved in DMSO and used to supplement cell culture media to the final given concentrations.

Two 21-nucleotide sequences targeting human STAT1 (designated c48 and c660, based on cDNA position) were subcloned in the pLVTHM genome vector (Addgene plasmid 12247) according to the manufacturer’s protocol. Briefly, siSTAT1-c48 sense oligonucleotide 5′-CGCGTCCCCGCAGGTTCACCAGCTTTATGATTCAAGAGATCATAAAGCTGGTGAACCTGCTTTTTGGAAAT-3′ and siSTAT1-c48 antisense oligonucleotide 5′-CGATTTCCAAAAAGCAGGTTCACCAGCTTTATGATCTCTTGAATCATAAAGCTGGTGAACCTGCGGGGA were annealed, and siSTAT1-c660 sense oligonucleotide 5′-CGCGTCCCCGCTGAATGTCACTGAACTTACTTCAAGAGAGTAAGTTCAGTGACATTCAGCTTTTTGGAAAT-3′ and siSTAT1-c660 antisense oligonucleotide 5′-CGATTTCCAAAAAGCTGAATGTCACTGAACTTACTCTCTTGAAGTAAGTTCAGTGACATTCAGCGGGGA were also annealed, and each set of annealed oligonucleotides separately cloned into the MluI/ClaI digested vector.

This approach allowed generation of a stem-loop-stem small hairpin RNA to effectively reduce PTEN expression levels (45). Self-inactivating (SIN) lentiviral vector stocks pseudotyped with the vesicular stomatitis virus-G envelope protein (VSV-G) were prepared by transient calcium phosphate transfection of the human embryonic kidney 293T cell line as previously described. Viral titers were estimated using the p24 capsid protein measured by enzyme linked immunosorbent assay (46).

TEC 1A3 AIRE^hi^ cells were transduced with either lentiviral vectors carrying the STAT1 knockdown small interfering RNA (multiplicity of infection = 100) or the scrambled small interfering RNA (multiplicity of infection = 100). Transduced cells were cultured for 7 days, before being harvested for analysis by QPCR and western blotting.

## Results

### Site-Specific Recombination to Generate rAIRE Expression Variants of TEC 1A3 Cells

**Figure 1:**
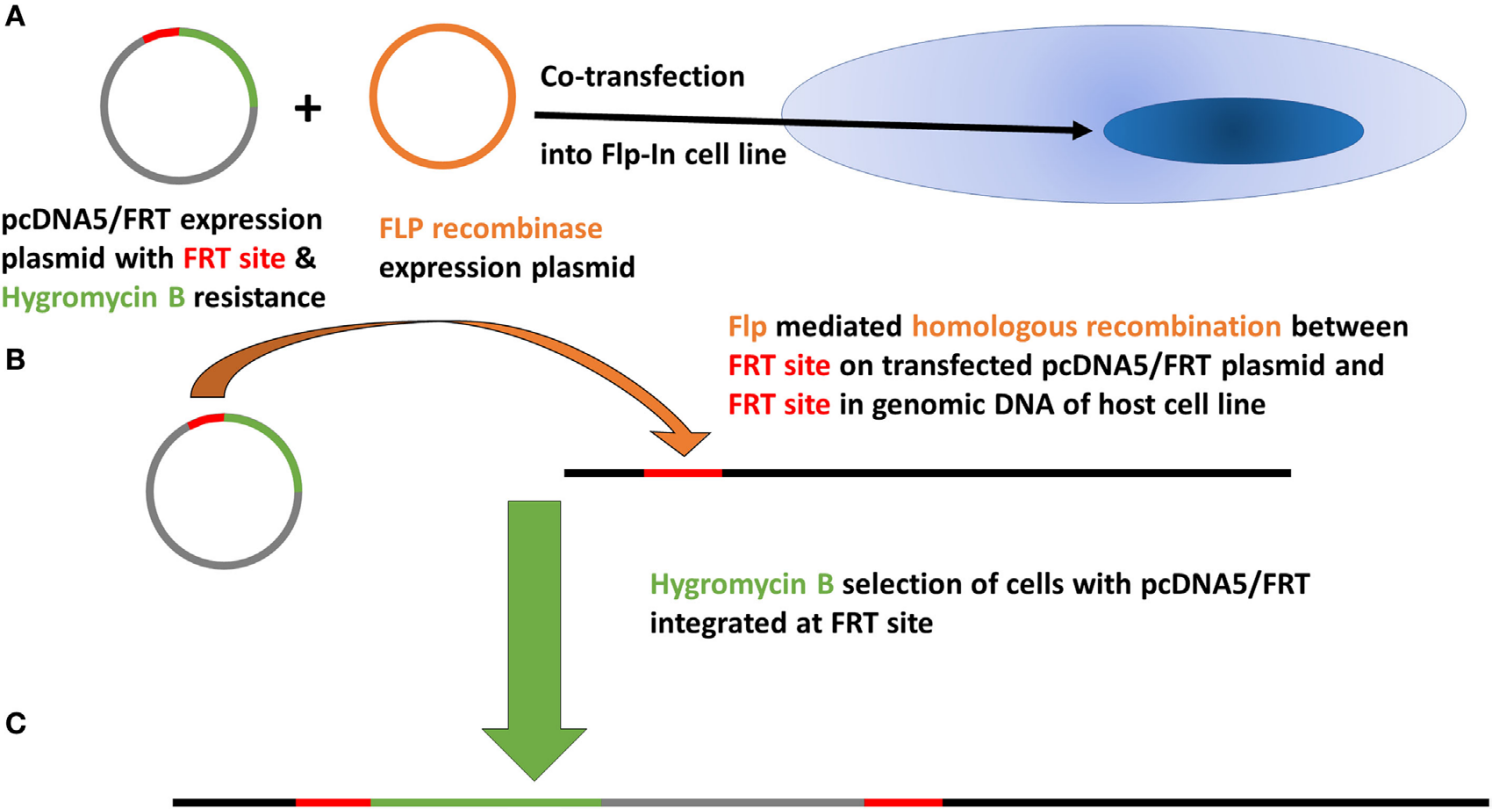
Basic principle of the Flp-In system of site-specific recombination to integrate a single copy of the pcDNA5/FRT expression vector containing autoimmune regulator cDNA into the genome of the target host cell line (TEC 1A3—FRT).

**Figure 2:**
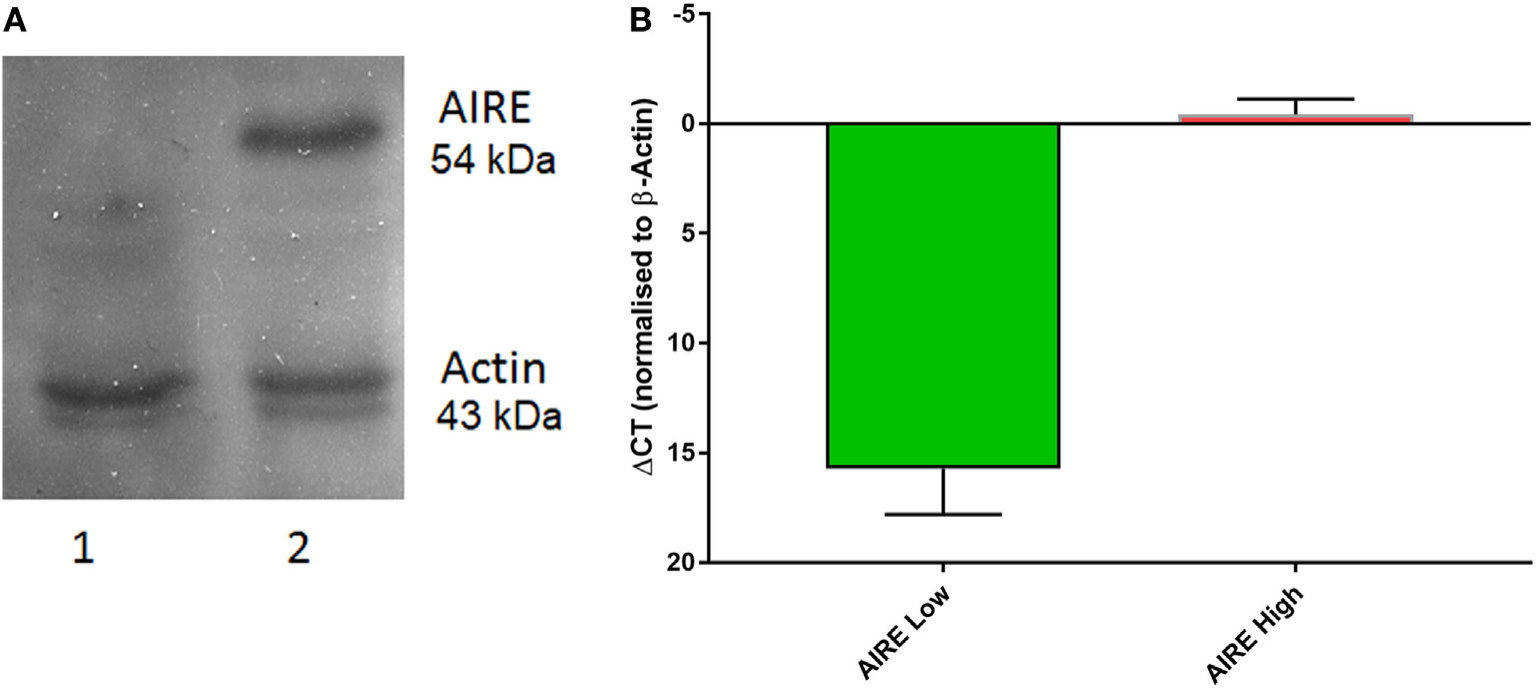
Autoimmune regulator (AIRE) expression in TEC 1A3. **(A)** Western blot of protein lysates from TEC 1A3 flp-in host cell line (lane 1) and TEC 1A3 recombinant AIRE overexpression cell line (lane 2). **(B)** QPCR determined relative expression levels of AIRE (γCt) after normalization against beta-actin in AIRE-low cells (1; green) and AIRE-high cells (10; red). *Y*-axis was switched to inverse (γCt) so that values go upwards.

Native AIRE expression in the TEC 1A3 cell line was found to be too low for reliable measurement when tested using western blotting and quantitative PCR (QPCR) (data not shown). Therefore, AIRE knockdown was excluded as an appropriate experimental option and a rAIRE expression variant of this cell line model was generated instead. The flp-in system (Invitrogen) of site-specific recombination was used to integrate a rAIRE expression vector into the genome of the TEC 1A3 cell line to minimize experimental variation, which could result from transient transfection (Figure 1). A major advantage of this method over those relying on random integration is that only a single copy of the expression vector is integrated into a single specific location (the Flp recombinase target site—FRT) of the host cell line’s genome. Southern blotting and β-galactosidase activity assays were used to identify a suitable TEC 1A3 flp-In host cell line variant from several isolated individual cells lines generated from the transfection of unmodified TEC 1A3 cells with the pFRT/*LacZeo* vector and subsequent antibiotic selection using zeocin of those which had the vector integrated into their genome (data not shown) (47). This final isogenic TEC 1A3 flp-in host cell line, containing only a single copy of the pFRT/*LacZeo* vector integrated into a transcriptionally active region of the genome, was used in the second stage to generate our cell line model. These cells were co-transfected with the rAIRE expression vector (pcDNA5/FRT-rAIRE) and a Flp recombinase expression vector (pOG44). Antibiotic selection conditions were again used to select for successful recombinants, using hygromycin B instead of zeocin, with initial AIRE expression in these cell lines determined by western blotting (Figure 2A) and QPCR (Figure 2B). Subsequent analysis of AIRE expression by QPCR showed that expression had increased significantly in the rAIRE expression cell lines compared to the level found in the TEC 1A3 flp-in host cell line. Sequencing across the FRT integration site confirmed that pcDNA5/FRT-rAIRE was integrated into the genome at the FRT site (data not shown). Our human thymic cell line model system to identify downstream human gene targets of AIRE consisted of the TEC 1A3 flp-in host cell line (AIRE^lo^) and the rAIRE expression TEC 1A3 cell line (AIRE^hi^).

### AIRE-Regulated Gene Expression in Human Cells

The transcriptional profile of the TEC 1A3 AIRE^lo^ and TEC 1A3 AIRE^hi^ cell lines was determined using the Affymetrix Human Genome U133 Plus 2.0 array. To compare our data with previously published murine data, we applied stringent conditions with Benjamini–Hochberg correction, FDR < 5%, and fold-change ≥ 1.2 to determine gene-differentially expressed with *P* ≤ 0.05. There were 555 probes differentially expressed (Table S1 in Supplementary Material), with 398 probes (including one specific for AIRE) displaying upregulated levels of expression in the AIRE^hi^ compared to the AIRE^lo^ cell line. There were 21 probes (9 upregulated, 16 downregulated) that did not have a corresponding gene annotated. Differential expression was identified on several other probes that corresponded to the same gene. Overall, this data set indicates differential expression for 482 different genes, with 353 upregulated and 129 downregulated in response to AIRE expression. This represents a larger repertoire, with a higher stringency of ARGs validation than has been previously reported using a human model (Table 1). The list of AIRE-regulated genes determined in our model was briefly compared with published lists of genes reported to be AIRE- or Aire-regulated in other studies, which identified a number of genes found to be similarly regulated in both our study and in one or more prior studies (7, 36, 39, 48, 49). Our model was validated by QPCR (Table 2), confirming differential expression of the most upregulated gene (*CCL5*) and downregulated gene (*MAGEB2*), alongside AIRE and a selection of the genes found to be expressed in an AIRE/AIRE-dependent manner in this study and at least one other study. Thymic expression was also confirmed for most of these genes by RT-PCR using commercially available human thymic cDNA (Table 2). To determine whether the increase of AIRE expression in AIRE^hi^ cells regulates the expression of genes encoding tissue-specific autoantigens in APS-1, we analyzed microarray data but before applying Benjamini–Hochberg correction for multiple testing. Six genes encoding APS-1 antigens were differentially expressed with *P* < 0.05 including CaSR, CYP21A, CYP1A2, SOX9, TGM4, and TDRD6 of which CaRS and CYP21A (TEC 1A3 cell line dataset: GSE35244) were also differentially expressed in two murine data sets (GSE8564, GSE12073).

**Table 1:** Comparison of different human cell line models of autoimmune regulator (AIRE)/AIRE-regulated gene expression.

Study	Cell line	AIRE expression	Expression analysis array	No. AIRE-regulated genes	
				*P* ≤ 0.05	*P* ≤ 0.05 (after correction^a^)
Current	TEC1A3 (thymus)	Site-specific recombination recombinant AIRE (rAIRE) expression	Affymetrix human genome U133 Plus 2.0 array (over 47,000 transcripts)	5,950	512

	U937 (MoDCs)	Stable transfection rAIRE expression	Atlas array human hematology/immunology nylon membranes and custom cDNA chip (1,972 separate transcripts)	72	n/a

	HEK293 (Kidney)	Stable transfection rAIRE expression	Illumina Human WG-6_V2_0_R2 BeadChip (over 48,000 transcripts)	294	n/a

	HEK293 (Kidney)	Transient transfection rAIRE expression	Affymetrix Human Genome U133 Plus 2.0 array (over 47,000 transcripts)	1,914	0

*^a^Benjamini–Hochberg correction for multiple testing, with false discovery rate <5%*.

**Table 2:** List of genes identified to be regulated in an autoimmune regulator (AIRE)-dependent manner in this study and showing at least one other study and analyzed in validation by QPCR and RT-PCR.

Gene symbol	Entrez Gene I.D.	Observed change in expression (microarray)	Validation QPCR	Thymus RT-PCR	Studies determining Aire/autoimmune regulator (AIRE)-regulated status
**Upregulated genes**					
AIRE	326	20.66	72214	+	
CAMK2B^a^	816	1.78 and 2.05	1.83	+	(7)
CCL3	414062	3.92	9.32	+	(12, 15, 30)
CCL5^a^	6352	14.98 and 16.70	38.32	+	(12, 15, 29)
CEACAM1	634	2.94	5.10	+	(15)
IL6	3569	4.02	4.94	+	(30)
KRT14	3861	1.52	5.21	+	(8)
KRT17	3872	7.88	102	+	(7, 15)
LEFTY2	7044	15.63	14395	+	(7, 15)
OAS3	4940	1.64	1.54	+	(7)

**Downregulated genes**					
DUSP4	1846	−1.59	0.32	+	(7)
MAGEB2	4113	−22.33	0.0004	–	
TCEA1	6917	−1.48	0.55	+	(30)

*Change in expression calculated as 2^n^, where n = difference between mean normalized expression values*.

*^a^Significant change in expression observed for two probes corresponding to this gene*.

### AIRE Preferentially Upregulates Genes With Low Levels of Expression in the Absence of AIRE

**Figure 3:**
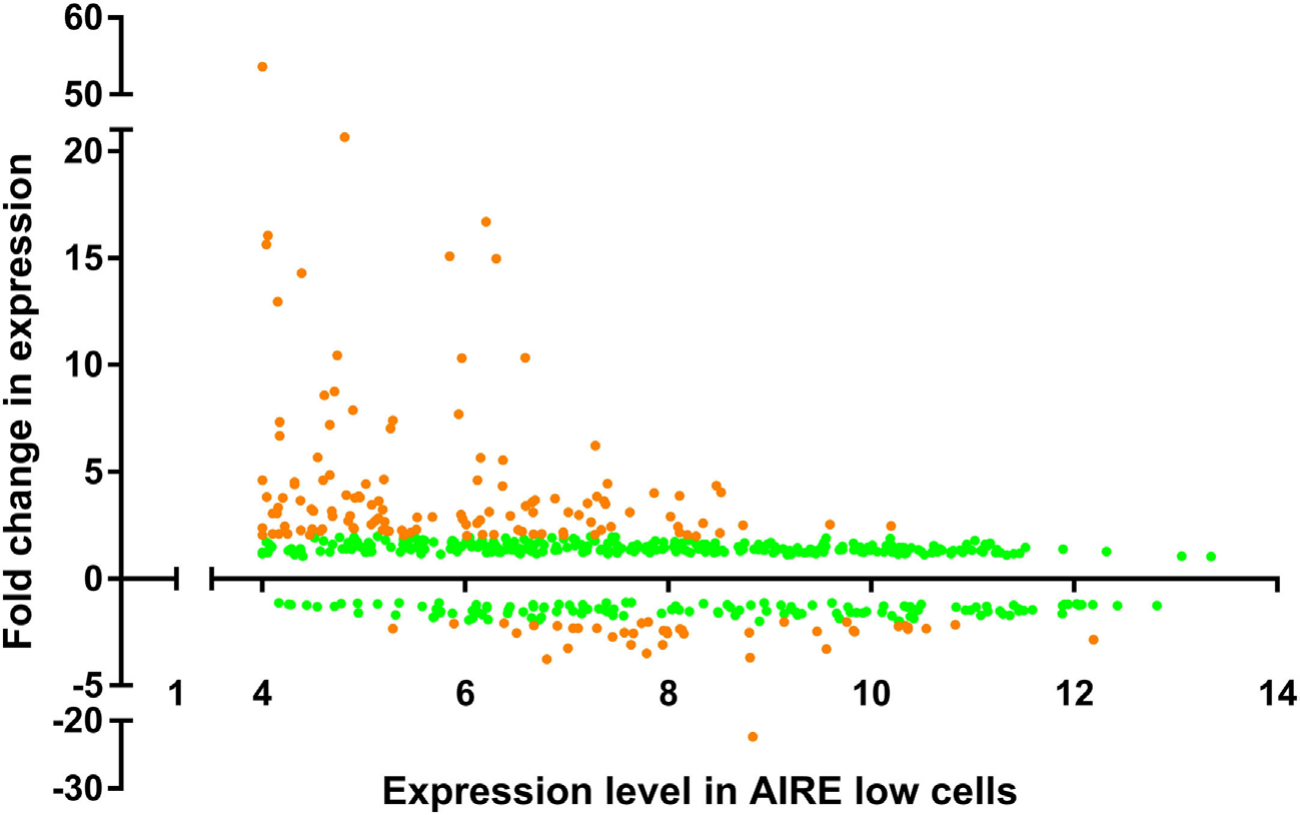
Gene expression profile of TEC 1A3 AIRE^lo^ cells vs relative change in expression between AIRE^hi^ and AIRE^lo^ cells. Gene expression values are averaged from three replicates and relative change in expression values calculated for all probes on the Human Genome U133 Plus 2.0 gene array. All dots show probes with a significant change in expression (*P* ≤ 0.05 with Benjamini–Hochberg correction), and orange dots show probes with significant change in expression ≥ 2.

The relative fold change in expression for our dataset, when compared against the expression levels in the AIRE^lo^ cell line variant (Figure 3), shows that the upregulated genes are not normally distributed (*P* < 0.0001) and, ignoring outliers, the expression values are positively skewed (skewness = 4.25), whereas the downregulated genes were not skewed as heavily (skewness = −1.492). The positively skewed distribution of expression values for AIRE upregulated genes in the AIRE^lo^ cell line suggests that AIRE preferentially upregulates expression for genes that would otherwise be expressed at more basal levels (“inactive” genes), rather than those whose expression is already elevated (“active” genes). Subsequent analysis using the DAVID bioinformatics resource (43) highlighted a number of Kyoto encyclopedia of genes and genomes pathways containing AIRE/Aire-regulated genes from the conserved list of genes, although none of these pathways displayed an abundance of AIRE/Aire-regulated genes (4–8 genes per pathway), and none had significant *P*-values after correction for multiple testing (*P* > 0.05 with Benjamini–Hochberg correction), suggesting that specific pathways are not targeted by AIRE/ARGE.

### ARGE in Human Thymic Cells Maps to Genes Identified in Previous Murine Studies

To extend our preliminary comparison, we used Genespring XI to analyze the microarray data obtained by Guerau-de-Arellano et al. (38) and Venanzi et al. (39) from mTECS obtained from wild-type and *Aire* knockout mice on the B6, Balb/c, and NOD genetic backgrounds. These four different murine data sets (*P* ≤ 0.05 for each individual data set) were then individually compared to the repertoire of probes showing AIRE-dependent differential expression in our TEC 1A3 cell line model (also *P* ≤ 0.05). This enabled us to determine which of the identified human probes had at least one murine probe corresponding to a homologous gene also showing Aire-dependent differential expression. The four separate comparisons were then compiled, revealing over 2,000 human probes with a corresponding murine probe in at least one of the murine data-sets (*P* < 0.0095), but more importantly, we identified a conserved list of 428 different human genes, which were found to have AIRE-dependent differential expression in our TEC 1A3 data-set, had a homologous gene in at least three of the murine data sets and fold-change ≥ 1.2 for probes from at least three of the data-sets(*P* < 0.00005, FDR < 1%, Table S2 in Supplementary Material). This indicates that there are certain gene targets of AIRE/Aire-regulated expression that remain constant across different species and genetic backgrounds. These gene targets can be used as signposts to elucidate the mechanisms used by AIRE/Aire to determine its specificity of interaction.

### Conserved AIRE/Aire-Regulated Genes and Transcription Regulation Interaction

**Figure 4:**
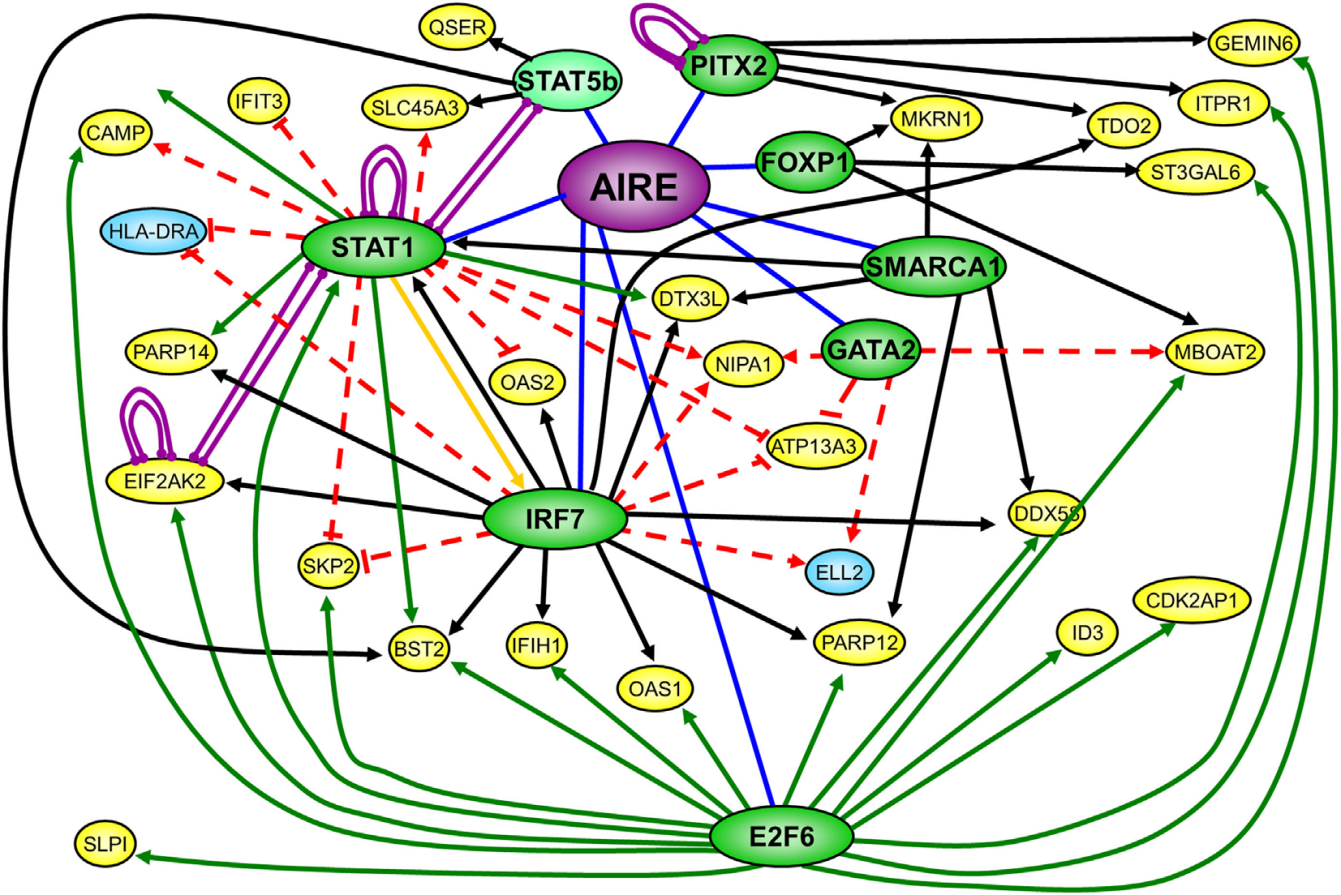
Summary of EdgeExpress DB output diagram of conserved list of autoimmune regulator (AIRE)/Aire-regulated genes showing key nodes with multiple edges and a selection of interacting nodes. Green nodes correspond to nodes reported with many edges, with dark green corresponding to AIRE/Aire upregulated genes, and light green to downregulated genes. Yellow and blue nodes correspond to the interacting nodes of AIRE/Aire upregulated and downregulated genes, respectively, with each node having three edges or less. Purple edges denote protein–protein interactions, black edges denote transcription factor-binding sites edges, green edges denote ChIP interaction edges, and red edges denote siRNA/miRNA perturbation edges.

We finally used FANTOM4 EdgeExpressDB (44) to identify any transcription regulatory interactions that might be present within our list of conserved ARGs. This analysis highlighted eight nodes present in our list of conserved genes, which had reported interactions (edges) to over 200 genes also present in our list of conserved ARGs. The nodes for E2F6, IRF7, and STAT1 each had over 30 edges with other ARGs, with a number of genes sharing edges with at least two nodes (Summary: Figure 4. Full: Figure S2 in Supplementary Material). It has previously been suggested that promiscuous expression in mTECs may operate *via* conserved transcriptional hierarchies (50), which is further supported by the EdgeExpressDB analysis of our conserved list of AIRE/Aire regulated genes. E2F6 was the node with the largest number of edges, the majority of these being ChIP interactions, corresponding to the reported binding of E2F6 (46) to the promoter of the gene with which it shares an edge. IRF7 and STAT1 were both nodes with a large number of perturbation edges corresponding to data obtained from siRNA/miRNA knockdown experiments, with IRF7 also having a number of edges corresponding to predictions of evolutionarily conserved transcription factor-binding sites (TFBS). A number of the conserved ARGs returned by the EdgeExpress analysis have been studied in investigations exploring autoimmune disorders associated with APS-1. These include OAS1, C4A, and IFIH1 for Type 1 diabetes (51–53) and ID3 and C4 for Sjögren’s syndrome (54, 55).

### AIRE Regulates Unphosphorylated STAT1 (U-STAT1)

Results from the EdgeExpress analysis suggest that for the identified gene network, AIRE may act *via* a hierarchical transcription factor model. We decided to examine this further and test a new hypothesis, which AIRE acts directly to regulate the expression of each node and indirectly *via* the nodes to affect the differential expression of the other genes in the network (see Figure 4; Figure S2 in Supplementary Material). As patients with *STAT1* gain-of-function (GOF) mutations also develop CMC and autoimmunity (56, 57), we focused on the STAT1 node, which is involved in the type I IFN system in antiviral defense, development of mTECs, and maintenance of thymic architecture (58, 59).

STAT1 has been shown to modulate the expression of a large number of genes when phosphorylated to its active form, primarily by Janus kinases as part of the JAK–STAT pathway. However, STAT1 in its unphosphorylated form (U-STAT1) still has transcriptional regulatory activity, albeit affecting a lower number of genes than its phosphorylated form. We, therefore, tested for STAT1 phosphorylation in our model and whether inhibition of STAT1 phosphorylation or knock down of STAT1 expression affected the expression of conserved ARGs that were linked to the STAT1 node in the EdgeExpress analysis (STAT1 edges). Most of the STAT1 molecules detected in TEC1 A3 were unphosphorylated. Cells treated with inhibitors of STAT1 phosphorylation, fludarabine, or LLL3, showed no difference in STAT1 between treated and untreated cells, nor in intracellular STAT1 phosphorylation status, as most STAT1 detected was unphosphorylated. The absence of phosphorylated STAT1 (p-STAT1) in TEC 1A3 cells was also observed in HaCat and Jurkat cells as well as in normal skin (RTA, unpublished), suggesting that p-STAT1 is not present in normal physiological conditions and the phosphorylation of STAT1 is associated with the reaction to inflammation.

### AIRE Simultaneously Regulates STAT1 and U-STAT1-Regulated Genes

**Figure 5:**
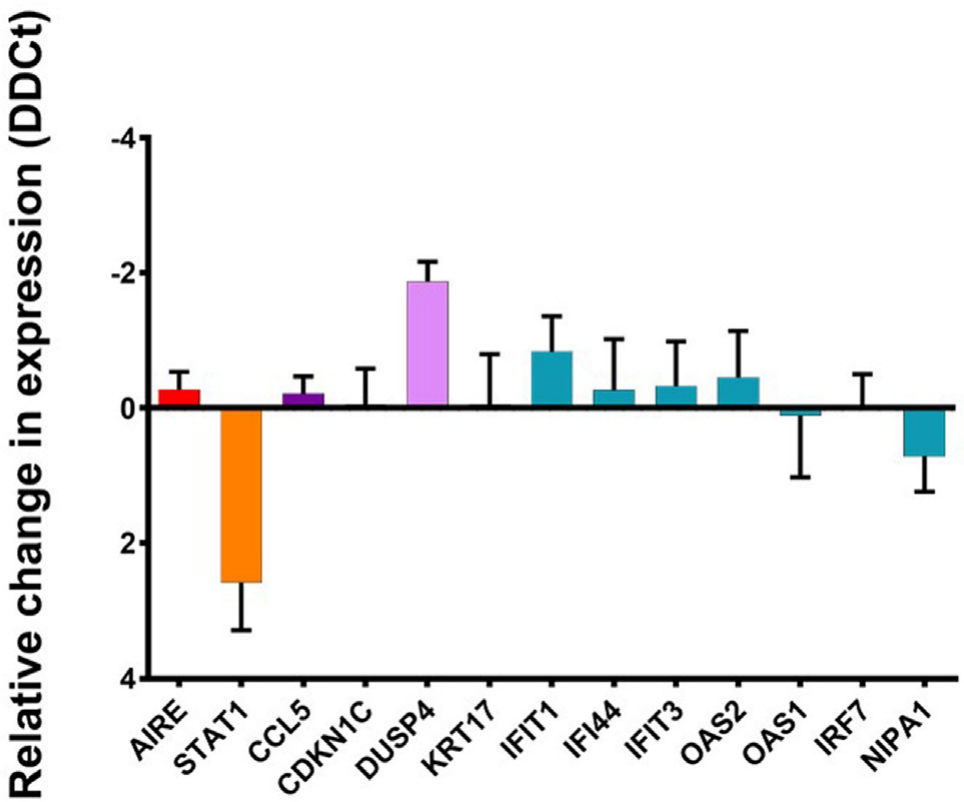
Change in relative expression levels of STAT1, autoimmune regulator (AIRE), and AIRE-dependent genes in TEC 1A3 AIRE^hi^ cells treated with either anti-STAT1 or scrambled shRNA. The expression of STAT1, AIRE, and AIRE-dependent genes were normalized to β-Actin, compared against values from the appropriate untreated cells after introducing lentiviral vectors that produce either a control “scrambled” shRNA or an anti-STAT1 shRNA. Genes tested split into the following groups: AIRE and STAT1 expression are indicated in red and orange, respectively. Genes upregulated by AIRE that are U-STAT1 independent: CCL5 and CDKN1C (dark purple). Genes downregulated by AIRE and previously reported as STAT1 regulated: KRT17 (gray) (61). Conserved genes upregulated by AIRE that were previously reported to be U-STAT regulated: IFIT1, IFI44, IFIT3, OAS2, OAS1, IRF7, and NIPA1 (blue) (60). DUSP4 (purple) is a conserved gene downregulated by AIRE that has been reported to interact functionally with STAT1 (62).

We knocked down the expression of STAT1 using two different lentivirus delivered anti-STAT1 shRNAs (shRNAc48 and shRNAc660) as well as scrambled shRNA as a negative control. We used QPCR to determine the change in relative expression level of STAT1, and when normalized to β-Actin expression, STAT1 shRNAc660 reduced the expression of STAT1 by more than fourfold with a lesser reduction in STAT1 expression detected when cells were transduced with STAT1 shRNAc48. Western blot analysis confirmed this reduction in STAT1 expression with both anti-STAT1 shRNAs whereas scrambled STAT1 shRNA showed no reduction of STAT1 protein compared to untreated cells (data not shown). Thus, we used STAT1 shRNAc660 to analyze the regulation of STAT1 downstream genes. Knowing that U-STAT1 is the main form of STAT1 present in our TEC 1A3 model, we analyzed the expression of a set of genes previously shown to be regulated by U-STAT1, including the AIRE-upregulated IFIT1, IFIT3, IFI44, IRF7, NIPA1, OAS1, and OAS2 (37, 60) as well as AIRE-regulated but U-STAT1-independent genes such as CCL5, CDKN1C, and KRT17. CCL5 and KRT17 expression are induced by p-STAT1 (38, 61) and, therefore, should not be affected by STAT1 knockdown. As expected, there was no significant change in the expression of AIRE, suggesting the addition of the viral vectors and the reduction of STAT1 does not affect the expression of AIRE. In addition, all the tested ARGs, which have been reported as U-STAT1 regulated, were unaffected by the reduction in the expression of STAT1 (Figure 5). Similar results were obtained with AIRE-dependent genes/U-STAT1 independent. A slight increase was observed in the case of IFIT1, but this was not significant. These results suggest that U-STAT1 is not involved in the regulation of these ARGs and, therefore, the observed increase in their expression was more directly mediated by AIRE, and not in an indirect manner *via* upregulated expression of an intermediate transcription factor. Surprisingly, DUSP4, which is a conserved gene downregulated by AIRE and reported to interact functionally with STAT1 (62), showed an increase in expression by twofold, suggesting that for downregulated ARGs, STAT1 may have a role in reducing expression of genes that have been reported elsewhere as being STAT1 regulated.

### Are AIRE-Downregulated Genes Dependent on U-STAT1?

**Figure 6:**
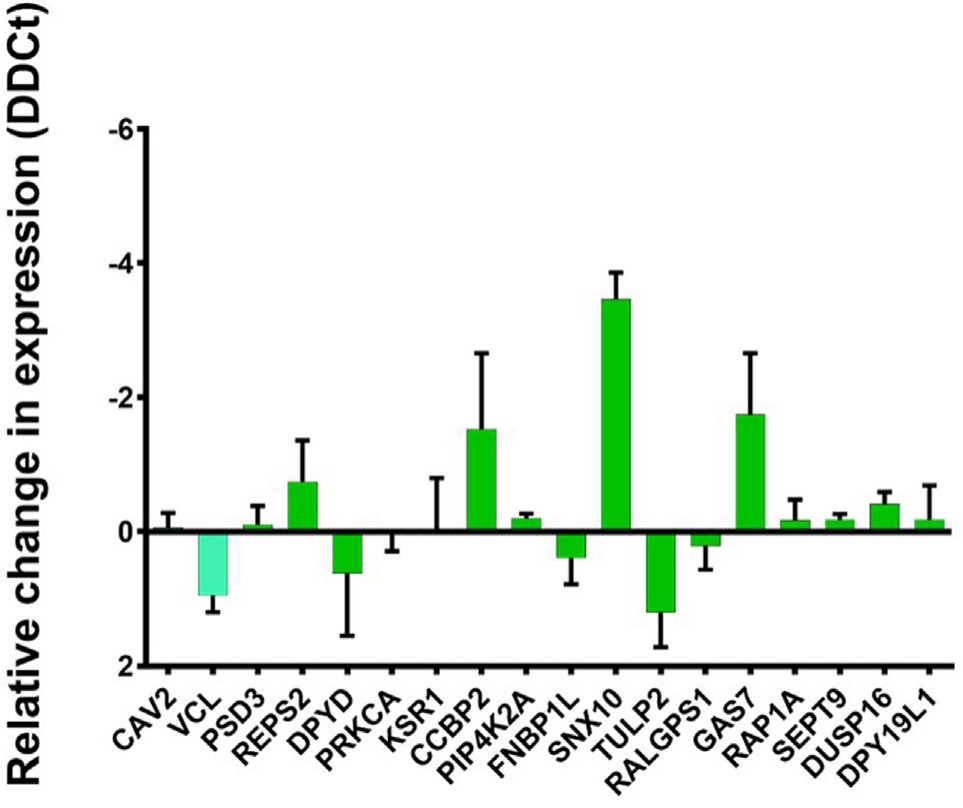
Change in relative expression levels of autoimmune regulator (AIRE) downregulated genes, previously reported to be U-STAT1 regulated, but not IFN-regulated. TEC 1A3 AIRE^hi^ cells were treated with either anti-STAT1 or scrambled shRNA, then the expression of 18 genes known to be regulated by AIRE and STAT1 were analyzed including 16 genes conserved ARGs (green barre) (59) and two genes conserved in ARG in TEC1 A3 only (58) (light blue barre). The knockdown of STAT1 revealed significant variation in relative change in expression (DDCt) of CC chemokine-binding protein 2 (CCBP2), growth arrest-specific protein 7 (GAS7), and sorting nexin 10 (SNX10), slightly variation (REPS2, VCL, DPYD, TULP2) or unchanged (CAV2, PSD3, PRKCA, KSR1, PIP4K2A, FNBP1L, RALGPS1, RAP1A, SETT9, DUSP6, and DPY19L1). DDCt values are averaged from three replicates.

To test this hypothesis, we selected 18 genes known to be downregulated by AIRE and STAT1. All these were among the conserved ARGs except CAV2 and VCL which were found among ARG in TEC1 A3 only. The knockdown of STAT1 revealed a mixed pattern. There were genes whose expression had been greatly affected and, therefore, seem dependent on U-STAT1, such as CC chemokine-binding protein 2 (CCBP2), growth arrest-specific protein 7 (GAS7), and sorting nexin 10 (SNX10) with an increase of 1.8, 2.0, and 3.8 fold, respectively. In contrast, expression of the other genes tested were either unchanged (CAV2, PSD3, PRKCA, KSR1, PIP4K2A, FNBP1L, RALGPS1, RAP1A, SETT9, DUSP6, and DPY19L1); slightly increased (REPS2); or slightly decreased (VCL, DPYD, TULP2). This suggests that the regulation of these genes is either completely or relatively independent of U-STAT1 (Figure 6).

## Discussion

Our cell line model has shown that AIRE up- and downregulates the expression of a number of genes in a ratio of approximately 2:1, as previously observed analysis of ARGE using microarrays (5, 36, 38, 39). As also reported in other studies, AIRE appears preferentially to upregulate the expression of genes with low levels of expression. This is in keeping with the suggested epigenetic mechanism of AIRE-dependent upregulation of expression, thus the binding of AIRE to unmethylated H3K4 could play a critical role in determining AIRE’s specificity for its targets. The targets of AIRE/Aire-regulated gene expression have an element of conservation, as AIRE-dependent differentially expressed genes identified in this study have been reported in other model systems that differ not only in genetic background, but also in either species or cell line origin. Genes identified as being AIRE/Aire-regulated across several different experimental models form an ideal list of candidate genes to be studied for elucidating the mechanism determining AIRE’s specificity mechanism for its downstream target genes. We were able to identify a larger number of AIRE-dependent differentially expressed genes than have been reported in other studies using human cell lines, while maintaining a FDR of less than 5%. The development of a cell line model facilitates experiments with inhibitors, activators, and RNAi, to explore specific elements of any potential mechanism. In addition, the use of the Flp-In system for this model means that it is possible to generate different recombinant cell line variants that differ by the changes introduced into the integrated expression vector, but not differ by the number of expression vectors integrated into the cell line genome nor by their integration/recombination site, thus reducing the scope for experimental variation between the different variants. Comparison of AIRE-dependent differential expression in our model with murine datasets identified 447 conserved genes, which represent only a portion of AIRE-regulated genes thought to be more than two thousands (2, 39), suggesting significant variation in the set of AIRE/Aire-regulated genes between humans and mice. Our model is a good one for studying AIRE as a transcription factor and its mechanism of action but less developed for analysis of APS-1. For instance, only CaSR, CYP21A, CYP1A2, SOX9, and TGM4 out of at least 20 TSA’s were regulated by AIRE in TEC 1A3 AIRE^hi^. This is not surprising; like in TEC 1A3, most of genes encoding APS-1 antigens were not differentially expressed in APS-1 mouse models (5, 34–39). Pontynen et al.’s findings were in keeping with these observations by showing that Aire-deficient mice had no reactivity against any of 11 out of the 17 known APS-1 autoantigens (63). This variation also depends on mouse background where B6 background is the least autoimmune model (63).

Another limitation of our model is the number of AIRE-regulated genes. More than 2,300 genes have been shown to be upregulated over twofold in mTEC^hi^ cells (64), but only 353 genes were upregulated in our model, which makes it less inclusive than the mouse models.

Patients with *STAT1* GOF mutations also develop CMC and autoimmunity (56, 57). These patients show increased STAT1 protein levels as well as an abnormal increase of p-STAT1 on stimulation with INF-g (57, 65). In contrast, APS-1 patients show decreased total STAT1, as well as lower peak levels of p-STAT1 after INF-γ stimulation (66). Therefore, it was of particular interest that our data showed that STAT1 is possibly regulated by AIRE. In our model, STAT1 was not found to be classically phosphorylated and the expression profile of our test panel of ARGs linked to STAT1 was not consistently affected when these cells were treated with STAT1 phosphorylation inhibitors. Similarly, on silencing STAT1 in our cell models, we did not observe any consistent change in the expression profile of our test panel of ARGs for either cell type or for either shRNA used. Based on these findings, we could not confirm the hypothesis that AIRE acts *via* the STAT1 node indirectly to affect the differential expression of the non–node genes downstream in the proposed network. This concurs with the results of Danso-Abeam et al. (50) who also explored the hierarchical transcription factor model of ARGE. They focused on the transcription factor Pdx1 in murine mTECs, whose promiscuous expression has been shown to be regulated in an Aire-dependent manner, and found that Pdx1 deficiency did not lead to a reduction inexpression of the expected downstream genes. Gucy2d (45) has been proposed as an intermediate transcription factor with Aire operating as part of a network. While there is strong bioinformatic evidence that Gucy2d is a node in a network of ARGs, it has unfortunately not been verified whether it acts as an intermediate transcription factor in keeping with the hierarchical transcription factor model based on a cascade-like transcriptional control of promiscuous gene expression in mTEC (45). It is important to mention here that genes not showing altered expression after adding STAT1 shRNA may actually be STAT1 dependent. There was no variation in the expression of these genes because STAT1 knockdown was incomplete. Therefore, these experiments should be performed using a different set of shRNAs to completely switch off STAT1.

Evidence is beginning to accumulate, that when looking at global ARGE patterns from pooled cells, AIRE does not operate *via* intermediate transcription factors according to the hierarchical transcription factor model to regulate the thousands of genes promiscuously expressed as a result of AIRE activity. However, as the range of ARGs has been found to differ across individual mTECs (42), it is possible that promiscuously expressed transcription factors may modulate the expression of their downstream target genes at a local level, but not sufficiently or consistently enough across all cells to produce a significant global effect when the transcriptomes of many cells are pooled. On the other hand, relatively little is known about the mechanism by which AIRE downregulates genes. In the case of U-STAT1, a least some genes do not seem to be directly regulated by AIRE, for which the hierarchical transcription factor model may apply, whereas others seem to be completely independent of U-STAT1. For instance, the dual-specific phosphatase (DUPS4), which is involved in blocking the activation of the extracellular regulated MAP kinase, has previously been shown to interact functionally with STAT1 in mediating sensitivity to erlotinib in human glioblastoma multiforme cell lines (62). This is an excellent example of genes downregulated by AIRE *via* another functionally related molecule. Such a relationship is not clear for other genes, including SNX10 with expression levels approximately fourfold greater when STAT1 was knocked down. SNX10 is believed to be a regulator of macrophage polarization (67). SNX10 has also been shown to have potential role in liver cancer by suppressing microRNA-30d (68). CCBP2 and GAS7 are two other genes inhibited by U-STAT1. CBBP2 is a chemokine decoy receptor and has been shown to be associated with metastatic breast cancer (69) whereas GAS7 has been shown to regulate neuronal cell morphology (70). Little is known about regulation of SNX10, CBBP2, and GAS7 expression, and it is unclear what the exact functional relationship is between U-STAT1 and these genes. Finally, systematic screening of AIRE downregulated genes, under each of the nodes shown in Figure S2 in Supplementary Material, is necessary to establish the full picture with details of genes that are directly regulated by AIRE and those indirectly regulated, i.e., *via* an intermediate transcription factor node in the proposed network. Interestingly, a recent study showed that APS-1 patients have 20% decrease of total STAT1 protein in human monocytes (66). Our findings, showing a moderate activation of STAT1 by AIRE in TEC1A3 cells, are in keeping with the study by Zimmerman et al., suggesting that STAT1 activation is at least in part AIRE-dependent. Such activation could have significant impact on several physiological processes including thymic negative selection leading to deletion of autoreactive T-cells and presentation of STAT1 antigens by mTEC (5), as well as maintaining STAT1 expression in monocytes to levels that allow these cells to have an adequate response to pathophysiological changes such as INF-γ stimulation (66).

Despite the relatively modest number of AIRE-regulated genes detected in TEC 1A3 AIRE^hi^ compared to APS-1 mouse models, the system we have presented here is robust and offers a new tool to dissect the molecular mechanism underlying ARGE.

## Author Contributions

Conceived and designed the experiments: TL, MA, and RT-A. Performed the experiments: TL. Analyzed the data: TL and RT-A. Contributed reagents/materials/analysis tools and wrote the paper: TL, AGM, AJM, MA, and RT-A. Designed the study and put experimental plan for it: TL and RT-A.

## Conflict of Interest Statement

The authors declare that the research was conducted in the absence of any commercial or financial relationships that could be construed as a potential conflict of interest.

## Footnotes

^1^http://david.abcc.ncifcrf.gov/ (Accessed: March, 2013).

^2^http://fantom.gsc.riken.jp/4/edgeexpress/about/ (Accessed: March, 2013).

## Supplementary Material

Figure S1 Gene expression profile of TEC 1A3 AIRE^lo^ cells vs relative change in expression between AIRE^hi^ and AIRE^lo^ cells. Gene expression values are averaged from three replicates and calculated relative change in expression values for all probes on the Human Genome U133 Plus 2.0 gene array. All dots show probes with a significant change in expression (*P* ≤ 0.05) with differential expression found to be conserved in at least three of the murine microarray analyses used in the comparison.

Figure S2 Full EdgeExpress DB output figure generated from list of human Entrez Gene IDs from conserved list of Autoimmune Regulator/Aire regulated genes using default settings. Node and edge colors and styles are as determined by EdgeExpress db.

Table S1 List of differentially expressed genes identified in *t*-test comparison of microarray data (*P* ≤ 0.05 with Benjamini–Hochberg correction) generated from TEC 1A3 AIRE^lo^ cells and TEC 1A3 AIRE^hi^ cells (GSE35244).

Table S2 List of autoimmune regulator (AIRE)/Aire-regulated genes with genes showing conservation in TEC 1A3 data set and at least three murine data sets. Data compiled from repertoires of AIRE and Aire-regulated genes determined from *t*-test analysis of microarray data generated from TEC 1A3 cells (GSE35244), and medullary thymic cells from C57BL/6 mice, BALB/c mice and NOD mice for two datasets [GSE12073 (38) and GSE8563 (39)]. All possible permutations of microarray probes (when more than one for a particular conserved gene) are shown.

Table S3 List of gene-specific primer sequences used for QPCR assays, with PrimerBank (49) reference number where appropriate.
