# Supplementary figures and images for: Meta-Analysis of Autoimmune Regulator-Regulated Genes in Human and Murine Models: A Novel Human Model Provides Insights on the Role of Autoimmune Regulator in Regulating STAT1 and STAT1-Regulated Genes
Source: Front Immunol. 2018 Jun 28;9:1380. doi: 10.3389/fimmu.2018.01380 (PMC6031710; doi:10.3389/fimmu.2018.01380)

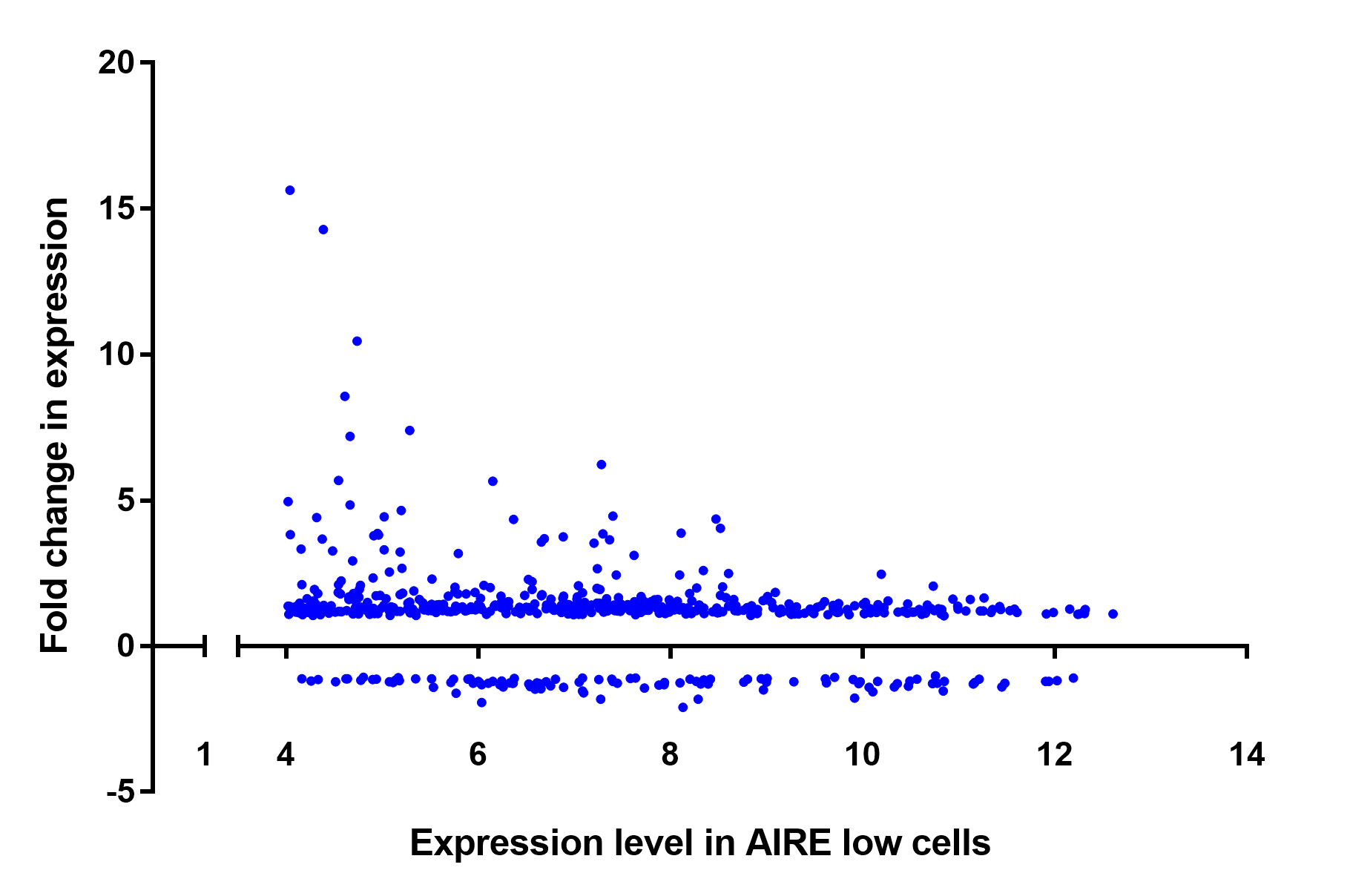

Supplement: Figure S1 — Gene expression profile of TEC 1A3 AIRElo cells vs relative change in expression between AIREhi and AIRElo cells. Gene expression values are averaged from three replicates and calculated relative change in expression values for all probes on the Human Genome U133 Plus 2.0 gene array. All dots show probes with a significant change in expression (P ≤ 0.05) with differential expression found to be conserved in at least three of the murine microarray analyses used in the comparison. [file image_1.tif]
