# Supplementary material for: Meta-Analysis of Autoimmune Regulator-Regulated Genes in Human and Murine Models: A Novel Human Model Provides Insights on the Role of Autoimmune Regulator in Regulating STAT1 and STAT1-Regulated Genes
Source: Front Immunol. 2018 Jun 28;9:1380. doi: 10.3389/fimmu.2018.01380 (PMC6031710; doi:10.3389/fimmu.2018.01380)
Supplement: Table S2 — List of autoimmune regulator (AIRE)/Aire-regulated genes with genes showing conservation in TEC 1A3 data set and at least three murine data sets. Data compiled from repertoires of AIRE and Aire-regulated genes determined from t-test analysis of microarray data generated from TEC 1A3 cells (GSE35244), and medullary thymic cells from C57BL/6 mice, BALB/c mice and NOD mice for two datasets [GSE12073 (38) and GSE8563 (39)]. All possible permutations of microarray probes (when more than one for a particular conserved gene) are shown. [file table_2.PDF]

Human Probe Set ID	Murine Probe Set ID	Regulation (TEC 1A3)	Fold Change Absolute (TEC 1A3)	Regulation (B6)	Fold Change Absolute (B6)	Regulation (BALB/c)	Fold Change Absolute (BALB/c)	Regulation (NOD #1)	Fold Change Absolute (NOD #1)	Regulation (NOD #2)	Fold Change Absolute (NOD #2)	Entrez Gene (Human)	Gene Symbol (Human)	Gene Title (Human)	UniGene ID (Human)
209460_at	1433855_at	up	1.271	up	1.703	up	1.434			up	1.699	18	ABAT	4-aminobutyrate aminotransferase	Hs.336768
209459 s at	1433855 at	up	1.788	up	1.703	up	1.434			up	1.699	18	ABAT	4-aminobutyrate aminotransferase	Hs.336768
1553604 at	1440370 at	up	1.307	up	2.566					up	1.883	154664	ABCA13	ATP-binding cassette, sub-family A (ABC1), r	Hs.226568
1553605 a at	1440370 at	up	1.404	up	2.566					up	1.883	154664	ABCA13	ATP-binding cassette, sub-family A (ABC1), r	Hs.226568
1553604 at	1446715 at	up	1.307	up	3.057			up	1.601			154664	ABCA13	ATP-binding cassette, sub-family A (ABC1), r	Hs.226568
1553605 a at	1446715 at	up	1.404	up	3.057			up	1.601			154664	ABCA13	ATP-binding cassette, sub-family A (ABC1), r	Hs.226568
226925 at	1456735 x at	up	1.279	up	1.631	up	1.715	up	1.642	up	1.748	92370	ACPL2	acid phosphatase-like 2	Hs.657887
226925 at	1429390 at	up	1.279	up	1.174							92370	ACPL2	acid phosphatase-like 2	Hs.657887
209765 at	1418402 at	down	1.246	down	2.641	down	1.991	down	2.237	down	1.960	8728	ADAM19	ADAM metallopeptidase domain 19 (meltrin b	Hs.483944
209765 at	1418403 at	down	1.246	down	1.949							8728	ADAM19	ADAM metallopeptidase domain 19 (meltrin b	Hs.483944
231703 s at	1422070 at	up	1.203	up	2.602	up	1.841	up	1.761	up	2.016	127	ADH4	Alcohol dehydrogenase 4 (class II), pi polypep	Hs.1219
217761 at	1438759 x at	up	1.563			up	1.630			up	1.322	55256	ADI1	acireductone dioxygenase 1	Hs.502773
222400 s at	1438759 x at	up	1.702			up	1.630			up	1.322	55256	ADI1	acireductone dioxygenase 1	Hs.502773
217761 at	1449076 x at	up	1.563					up	1.306	up	1.697	55256	ADI1	acireductone dioxygenase 1	Hs.502773
222400 s at	1449076 x at	up	1.702					up	1.306	up	1.697	55256	ADI1	acireductone dioxygenase 1	Hs.502773
217761 at	1438758 at	up	1.563	up	1.491	up	1.516					55256	ADI1	acireductone dioxygenase 1	Hs.502773
222400 s at	1438758 at	up	1.702	up	1.491	up	1.516					55256	ADI1	acireductone dioxygenase 1	Hs.502773
237281 at	1457541 at	up	1.241	up	1.792	up	2.669	up	1.779	up	1.654	158798	AKAP14	A kinase (PRKA) anchor protein 14	Hs.592245
204151 x at	1451030 at	up	2.013	up	3.058	up	1.255	up	1.993	up	1.854	1645	AKR1C1	aldo-keto reductase family 1, member C1 (dih	Hs.460260
216594 x at	1451030 at	up	2.298	up	3.058	up	1.255	up	1.993	up	1.854	1645	AKR1C1	aldo-keto reductase family 1, member C1 (dih	Hs.460260
211653 x at	1451030 at	up	2.549	up	3.058	up	1.255	up	1.993	up	1.854	1646	AKR1C2	aldo-keto reductase family 1, member C2 (dih	Hs.567256
209699 x at	1451030 at	up	2.670	up	3.058	up	1.255	up	1.993	up	1.854	1646	AKR1C2	aldo-keto reductase family 1, member C2 (dih	Hs.567256
209160 at	1419136 at	up	4.434	up	14.105	up	7.043	up	8.653	up	21.837	8644	AKR1C3	aldo-keto reductase family 1, member C3 (3-a	Hs.78183
212224 at	1416468 at	up	1.205	up	8.936	up	8.489	up	10.408	up	10.117	216	ALDH1A1	aldehyde dehydrogenase 1 family, member A1	Hs.76392
217238 s at	1451194 at	up	1.090	up	4.925			up	5.338	up	4.701	229	ALDOB	aldolase B, fructose-bisphosphate	Hs.530274
207328 at	1420338 at	up	1.227	up	1.973	up	1.628	up	1.770	up	1.795	246	ALOX15	arachidonate 15-lipoxygenase	Hs.73809
211036 x at	1453307 a at	up	1.175					up	1.269	up	1.343	51433	ANAPC5	anaphase promoting complex subunit 5	Hs.7101
211036 x at	1456695 x at	up	1.175					up	1.263	up	1.475	51433	ANAPC5	anaphase promoting complex subunit 5	Hs.7101
211036 x at	1435178 x at	up	1.175					up	1.302	up	1.488	51433	ANAPC5	anaphase promoting complex subunit 5	Hs.7101
211036 x at	1416906 at	up	1.175					up	1.374	up	1.562	51433	ANAPC5	anaphase promoting complex subunit 5	Hs.7101
211036 x at	1433822 x at	up	1.175	up	1.290			up	1.245			51433	ANAPC5	anaphase promoting complex subunit 5	Hs.7101
209442 x at	1451628 a at	up	1.123	up	1.389	up	1.632			up	1.442	288	ANK3		Hs.499725
206385 s at	1451628 a at	up	1.267	up	1.389	up	1.632			up	1.442	288	ANK3	ankyrin 3, node of Ranvier (ankyrin G)	Hs.499725
209442 x at	1452872 at	up	1.123	up	1.196	up	1.465					288	ANK3		Hs.499725
206385 s at	1452872 at	up	1.267	up	1.196	up	1.465					288	ANK3	ankyrin 3, node of Ranvier (ankyrin G)	Hs.499725
209442 x at	1452124 at	up	1.123			up	1.694					288	ANK3		Hs.499725
206385 s at	1452124 at	up	1.267			up	1.694					288	ANK3	ankyrin 3, node of Ranvier (ankyrin G)	Hs.499725
222608 s at	1433543 at	up	1.407	up	1.925			up	1.904	up	2.246	54443	ANLN	anillin, actin binding protein	Hs.62180
205083 at	1419435 at	up	1.264	up	2.738	up	2.490	up	2.777	up	2.445	316	AOX1	aldehyde oxidase 1	Hs.406238
1555731 a at	1455735 at	up	1.358	up	2.617	up	1.727	up	1.540			130340	AP1S3	adaptor-related protein complex 1, sigma 3 su	Hs.632555
201525 at	1416371 at	up	1.352	up	2.646	up	4.296	up	4.161	up	3.266	347	APOD	apolipoprotein D	Hs.522555
209047 at	1416203 at	up	2.923	up	4.618	up	4.039	up	4.407	up	4.256	358	AQP1	aquaporin 1 (Colton blood group)	Hs.76152
39248 at	1450460 at	up	7.396	up	4.604			up	3.149	up	2.397	360	AQP3	aquaporin 3 (Gill blood group)	Hs.234642
39248 at	1422008 a at	up	7.396					up	1.234			360	AQP3	aquaporin 3 (Gill blood group)	Hs.234642
225166 at	1431133 at	down	1.253					down	1.376	down	1.480	93663	ARHGAP18	Rho GTPase activating protein 18	Hs.486458
225171 at	1431133 at	down	1.279					down	1.376	down	1.480	93663	ARHGAP18	Rho GTPase activating protein 18	Hs.486458
225166 at	1426952 at	down	1.253	down	2.855	down	1.753					93663	ARHGAP18	Rho GTPase activating protein 18	Hs.486458
225171 at	1426952 at	down	1.279	down	2.855	down	1.753					93663	ARHGAP18	Rho GTPase activating protein 18	Hs.486458
224996 at	1436455 at	up	1.176							up	1.169	444	ASPH	aspartate beta-hydroxylase	Hs.591874
209135 at	1436455 at	up	1.298							up	1.169	444	ASPH	aspartate beta-hydroxylase	Hs.591874
224996 at	1420959 at	up	1.176					up	1.338	up	1.290	444	ASPH	aspartate beta-hydroxylase	Hs.591874
209135 at	1420959 at	up	1.298					up	1.338	up	1.290	444	ASPH	aspartate beta-hydroxylase	Hs.591874
224996 at	1426015 s at	up	1.176	up	1.966							444	ASPH	aspartate beta-hydroxylase	Hs.591874
209135 at	1426015 s at	up	1.298	up	1.966							444	ASPH	aspartate beta-hydroxylase	Hs.591874
218857 s at	1424395 at	up	1.112	up	1.782	up	1.607	up	1.431	up	1.622	80150	ASRGL1	asparaginase like 1	Hs.535326
218857 s at	1460360 at	up	1.112	up	2.575	up	1.809	up	1.611			80150	ASRGL1	asparaginase like 1	Hs.535326
218857 s at	1424396 a at	up	1.112	up	2.881	up	1.940	up	1.649			80150	ASRGL1	asparaginase like 1	Hs.535326
222740 at	1436174 at	up	1.588	up	1.408			up	1.524	up	1.696	29028	ATAD2	ATPase family, AAA domain containing 2	Hs.370834
218782 s at	1436174 at	up	1.632	up	1.408			up	1.524	up	1.696	29028	ATAD2	ATPase family, AAA domain containing 2	Hs.370834
228401 at	1436174 at	up	1.666	up	1.408			up	1.524	up	1.696	29028	ATAD2	ATPase family, AAA domain containing 2	Hs.370834
212297 at	1442145 at	up	1.259			up	1.199	up	1.318	up	1.300	79572	ATP13A3	ATPase type 13A3	Hs.529609
209186 at	1427251 at	up	1.287					up	1.056	up	1.092	488	ATP2A2	ATPase, Ca++ transporting, cardiac muscle,	Hs.506759
209186 at	1427250 at	up	1.287			up	1.358			up	1.366	488	ATP2A2	ATPase, Ca++ transporting, cardiac muscle,	Hs.506759
209186 at	1437797 at	up	1.287					up	1.310	up	1.474	488	ATP2A2	ATPase, Ca++ transporting, cardiac muscle,	Hs.506759
209186 at	1416551 at	up	1.287			up	1.505			up	1.594	488	ATP2A2	ATPase, Ca++ transporting, cardiac muscle,	Hs.506759
209186 at	1452363 a at	up	1.287			up	1.513			up	1.638	488	ATP2A2	ATPase, Ca++ transporting, cardiac muscle,	Hs.506759
216120 s at	1433888 at	up	1.359	up	2.305	up	1.729			up	1.542	491	ATP2B2	ATPase, Ca++ transporting, plasma membrar	Hs.268942
204092 s at	1424511 at	up	1.406	up	1.351	up	1.738			up	1.531	6790	AURKA	aurora kinase A	Hs.250822
208079 s at	1424511 at	up	1.430	up	1.351	up	1.738			up	1.531	6790	AURKA	aurora kinase A	Hs.250822
210535 at	1417596 at	up	1.313	up	1.208	up	1.472	up	1.119	up	1.127	27077	B9D1	B9 protein domain 1	Hs.462445
222446 s at	1438645 x at	up	1.437	up	2.983			up	2.197	up	1.964	25825	BACE2	beta-site APP-cleaving enzyme 2	Hs.529408
222446 s at	1435581 at	up	1.437	up	1.448	up	1.458					25825	BACE2	beta-site APP-cleaving enzyme 2	Hs.529408
222446 s at	1416673 at	up	1.437	up	1.895	up	1.627					25825	BACE2	beta-site APP-cleaving enzyme 2	Hs.529408
222446 s at	1437846 x at	up	1.437	up	3.607	up	1.866					25825	BACE2	beta-site APP-cleaving enzyme 2	Hs.529408
203304 at	1423753 at	up	2.002	up	2.164	up	2.122	up	1.700	up	1.905	25805	BAMBI	BMP and activin membrane-bound inhibitor h	Hs.533336
219497 s at	1456632 at	up	1.222	up	1.415	up	1.232	up	1.409			53335	BCL11A	B-cell CLL/lymphoma 11A (zinc finger protein	Hs.370549
219497 s at	1419406 a at	up	1.222	up	1.655							53335	BCL11A	B-cell CLL/lymphoma 11A (zinc finger protein	Hs.370549
205681 at	1419004 s at	up	1.336	up	1.902	up	1.464	up	1.660	up	1.619	597	BCL2A1	BCL2-related protein A1	Hs.227817
205681 at	1450812 at	up	1.336	up	1.180							597	BCL2A1	BCL2-related protein A1	Hs.227817
205681 at	1437913 at	up	1.336	up	1.638							597	BCL2A1		Hs.227817
204493 at	1417045 at	up	1.259	up	1.847	up	1.397	up	1.575	up	1.655	637	BID	BH3 interacting domain death agonist	Hs.591054
211725 s at	1417045 at	up	1.391	up	1.847	up	1.397	up	1.575	up	1.655	637	BID	BH3 interacting domain death agonist	Hs.591054
205733 at	1448953 at	up	1.529	up	1.386	up	1.899	up	1.518	up	2.305	641	BLM	Bloom syndrome, RecQ helicase-like	Hs.716515
226530 at	1454880 s at	down	1.615			down	1.466	down	1.274	down	1.358	90427	BMF	Bcl2 modifying factor	Hs.591104
201641 at	1424921 at	up	1.771	up	2.579	up	2.419					684	BST2	bone marrow stromal cell antigen 2	Hs.118110
229801 at	1456210 at	up	1.246	up	1.951	up	1.267			up	1.606	254427	C10orf47	chromosome 10 open reading frame 47	Hs.435775
219544 at	1433807 at	up	1.342					up	1.579	up	1.967	79866	C13orf34	chromosome 13 open reading frame 34	Hs.714340
219544 at	1429478 at	up	1.342	up	1.292							79866	C13orf34	chromosome 13 open reading frame 34	Hs.714340
1557755 at	1441867 x at	up	1.118	up	1.730	up	1.988	up	1.242			145508	C14orf145	chromosome 14 open reading frame 145	Hs.162889
1557756 a at	1441867 x at	up	1.309	up	1.730	up	1.988	up	1.242			145508	C14orf145	chromosome 14 open reading frame 145	Hs.162889
1557755 at	1429228 at	up	1.118			up	1.432					145508	C14orf145	chromosome 14 open reading frame 145	Hs.162889
1557756 a at	1429228 at	up	1.309			up	1.432					145508	C14orf145	chromosome 14 open reading frame 145	Hs.162889
1557755 at	1429229 s at	up	1.118	up	1.775	up	1.747					145508	C14orf145	chromosome 14 open reading frame 145	Hs.162889
1557756 a at	1429229 s at	up	1.309	up	1.775	up	1.747					145508	C14orf145	chromosome 14 open reading frame 145	Hs.162889
225300 at	1423464 at	up	1.498							up	1.207	90417	C15orf23	chromosome 15 open reading frame 23	Hs.525796
225300 at	1423463 a at	up	1.498					up	1.347	up	1.468	90417	C15orf23	chromosome 15 open reading frame 23	Hs.525796
225300 at	1442280 at	up	1.498			up	1.162	up	1.441	up	1.710	90417	C15orf23	chromosome 15 open reading frame 23	Hs.525796
225300 at	1420081 s at	up	1.498					up	1.241			90417	C15orf23	chromosome 15 open reading frame 23	Hs.525796
220840 s at	1437186 at	up	1.504					up	1.258	up	1.319	55732	C1orf112	chromosome 1 open reading frame 112	Hs.443551
220840 s at	1434365 a at	up	1.504	up	1.544			up	1.420	up	1.731	55732	C1orf112	chromosome 1 open reading frame 112	Hs.443551
223127 s at	1444235 at	down	1.226			down	1.187	down	1.332	down	1.268	81563	C1orf21	chromosome 1 open reading frame 21	Hs.497159
221272 s at	1444235 at	down	1.261			down	1.187	down	1.332	down	1.268	81563	C1orf21	chromosome 1 open reading frame 21	Hs.497159
223127 s at	1436431 at	down	1.226	down	1.341							81563	C1orf21	chromosome 1 open reading frame 21	Hs.497159
221272 s at	1436431 at	down	1.261	down	1.341							81563	C1orf21	chromosome 1 open reading frame 21	Hs.497159
218123 at	1437682 x at	up	1.145	up	1.890	up	2.759	up	1.627	up	1.687	56683	C21orf59	chromosome 21 open reading frame 59	Hs.5811
218123 at	1424315 at	up	1.145	up	1.745	up	2.927	up	1.787	up	1.829	56683	C21orf59	chromosome 21 open reading frame 59	Hs.5811
1559036 at	1418021 at	up	1.122			up	1.234	up	1.364	up	1.626	720 /// 721	C4A /// C4B	complement component 4A (Rodgers blood g	Hs.534847
226946 at	1437285 at	up	1.094	up	1.442			up	1.289	up	1.401	133686	C5orf33	chromosome 5 open reading frame 33	Hs.81907
226946 at	1437286 x at	up	1.094	up	1.731			up	1.478			133686	C5orf33	chromosome 5 open reading frame 33	Hs.81907
226946 at	1437287 at	up	1.094			up	1.377					133686	C5orf33	chromosome 5 open reading frame 33	Hs.81907
229070 at	1427957 at	down	1.269	down	1.710	down	1.605	down	2.517	down	2.541	84830	C6orf105	chromosome 6 open reading frame 105	Hs.126409
232038 at	1440928 at	up	1.246	up	1.243			up	1.231	up	1.248	221322	C6orf170	chromosome 6 open reading frame 170	Hs.121396
231270 at	1421308 at	up	1.496	up	1.734	up	1.513	up	1.623	up	1.668	377677	CA13	carbonic anhydrase XIII	Hs.127189
231270 at	1439620 at	up	1.496	up	2.423	up	1.518	up	1.802	up	2.197	377677	CA13	carbonic anhydrase XIII	Hs.127189
231270 at	1421307 at	up	1.496	up	2.377	up	1.852					377677	CA13	carbonic anhydrase XIII	Hs.127189
206208 at	1418094 s at	up	3.335	up	5.097	up	3.060	up	4.316	up	1.985	762	CA4	carbonic anhydrase IV	Hs.89485
206209 s at	1418094 s at	up	4.417	up	5.097	up	3.060	up	4.316	up	1.985	762	CA4	carbonic anhydrase IV	Hs.89485
206208 at	1448949 at	up	3.335	up	5.365	up	3.413	up	4.048	up	3.973	762	CA4	carbonic anhydrase IV	Hs.89485
206209 s at	1448949 at	up	4.417	up	5.365	up	3.413	up	4.048	up	3.973	762	CA4	carbonic anhydrase IV	Hs.89485
225531 at	1441938 x at	down	1.318			down	2.186	down	1.602			91768	CABLES1	Cdk5 and Abl enzyme substrate 1	Hs.11108
225532 at	1441938 x at	down	1.498			down	2.186	down	1.602			91768	CABLES1	Cdk5 and Abl enzyme substrate 1	Hs.11108
225531 at	1422477 at	down	1.318			down	1.628					91768	CABLES1	Cdk5 and Abl enzyme substrate 1	Hs.11108
225532 at	1422477 at	down	1.498			down	1.628					91768	CABLES1	Cdk5 and Abl enzyme substrate 1	Hs.11108
225531 at	1439778 at	down	1.318	down	1.219							91768	CABLES1	Cdk5 and Abl enzyme substrate 1	Hs.11108
225532 at	1439778 at	down	1.498	down	1.219							91768	CABLES1	Cdk5 and Abl enzyme substrate 1	Hs.11108
204811 s at	1450754 at	up	14.289			up	2.885	up	4.708	up	5.889	9254	CACNA2D2	calcium channel, voltage-dependent, alpha 2/	Hs.476273
221401 at	1434785 at	up	1.398	up	2.913	up	4.448	up	4.195	up	3.171	27091	CACNG5	calcium channel, voltage-dependent, gamma	Hs.278907
209030 s at	1431611 a at	down	1.379			down	1.134	down	1.356	down	1.327	23705	CADM1	cell adhesion molecule 1	Hs.370510
209032 s at	1431611 a at	down	1.407			down	1.134	down	1.356	down	1.327	23705	CADM1	cell adhesion molecule 1	Hs.370510
209031 at	1431611 a at	down	1.474			down	1.134	down	1.356	down	1.327	23705	CADM1	cell adhesion molecule 1	Hs.370510
209030 s at	1417376 a at	down	1.379							down	1.355	23705	CADM1	cell adhesion molecule 1	Hs.370510
209032 s at	1417376 a at	down	1.407							down	1.355	23705	CADM1	cell adhesion molecule 1	Hs.370510
209031 at	1417376 a at	down	1.474							down	1.355	23705	CADM1	cell adhesion molecule 1	Hs.370510
209030 s at	1417378 at	down	1.379					down	1.218			23705	CADM1	cell adhesion molecule 1	Hs.370510
209032 s at	1417378 at	down	1.407					down	1.218			23705	CADM1	cell adhesion molecule 1	Hs.370510
209031 at	1417378 at	down	1.474					down	1.218			23705	CADM1	cell adhesion molecule 1	Hs.370510
210349 at	1452572 at	down	1.217					down	1.157	down	1.147	814	CAMK4	calcium/calmodulin-dependent protein kinase	Hs.591269
210349 at	1421941 at	down	1.217	down	1.162			down	1.235			814	CAMK4	calcium/calmodulin-dependent protein kinase	Hs.591269

210349 at	1426167 a at	down	1.217	down	1.535							814	CAMK4	calcium/calmodulin-dependent protein kinase	Hs.591269
210244 at	1419691 at	up	1.252	up	5.932	up	7.000	up	6.083	up	4.795	820	CAMP	cathelicidin antimicrobial peptide	Hs.51120
201432 at	1416429 a at	down	1.201			down	1.611	down	1.437			847	CAT	catalase	Hs.502302
211922 s at	1416429 a at	down	1.201			down	1.611	down	1.437			847	CAT	catalase	Hs.502302
201432 at	1416430 at	down	1.201	down	1.450	down	1.742	down	1.509			847	CAT	catalase	Hs.502302
211922 s at	1416430 at	down	1.201	down	1.450	down	1.742	down	1.509			847	CAT	catalase	Hs.502302
1568850 at	1437937 at	down	1.250	down	1.867			down	1.385	down	1.262	1238	CCBP2	Chemokine binding protein 2	Hs.146346
1568850 at	1422112 at	down	1.250	down	1.457							1238	CCBP2	Chemokine binding protein 2	Hs.146346
232362 at	1454121 x at	up	1.210					up	1.299	up	1.474	343099	CCDC18	coiled-coil domain containing 18	Hs.716682
232362 at	1432113 at	up	1.210	up	1.114							343099	CCDC18	coiled-coil domain containing 18	Hs.716682
234995 at	1427031 s at	up	1.512	up	2.109	up	1.557	up	1.380	up	1.678	152185	CCDC52	coiled-coil domain containing 52	Hs.477144
213226 at	1417910 at	up	1.389	up	1.443			up	1.724	up	1.912	890	CCNA2	cyclin A2	Hs.58974
203418 at	1417910 at	up	1.606	up	1.443			up	1.724	up	1.912	890	CCNA2	cyclin A2	Hs.58974
213226 at	1417911 at	up	1.389	up	1.328							890	CCNA2	cyclin A2	Hs.58974
203418 at	1417911 at	up	1.606	up	1.328							890	CCNA2	cyclin A2	Hs.58974
1554283 at	1425837 a at	up	1.715	up	2.059	up	1.832	up	1.456	up	1.477	25819	CCRN4L	CCR4 carbon catabolite repression 4-like (S.	Hs.639842
209288 s at	1422642 at	down	1.398	down	1.920			down	1.879	down	1.764	10602	CDC42EP3	CDC42 effector protein (Rho GTPase binding	Hs.369574
209286 at	1422642 at	down	1.403	down	1.920			down	1.879	down	1.764	10602	CDC42EP3	CDC42 effector protein (Rho GTPase binding	Hs.369574
209288 s at	1450700 at	down	1.398					down	1.987	down	1.881	10602	CDC42EP3	CDC42 effector protein (Rho GTPase binding	Hs.369574
209286 at	1450700 at	down	1.403					down	1.987	down	1.881	10602	CDC42EP3	CDC42 effector protein (Rho GTPase binding	Hs.369574
224753 at	1416802 a at	up	1.307	up	1.551			up	1.300	up	1.579	113130	CDCA5	cell division cycle associated 5	Hs.434886
224753 at	1448466 at	up	1.307	up	1.341					up	1.731	113130	CDCA5	cell division cycle associated 5	Hs.434886
221520 s at	1428480 at	up	1.253	up	1.306					up	1.713	55143	CDCA8	cell division cycle associated 8	Hs.524571
221520 s at	1436847 s at	up	1.253					up	1.634	up	2.003	55143	CDCA8	cell division cycle associated 8	Hs.524571
221520 s at	1428481 s at	up	1.253	up	1.785							55143	CDCA8	cell division cycle associated 8	Hs.524571
204677 at	1433956 at	up	1.366			up	1.636	up	1.871	up	1.350	1003	CDH5	cadherin 5, type 2 (vascular endothelium)	Hs.76206
201938 at	1426885 a at	up	1.211					up	1.305	up	1.261	8099	CDK2AP1	cyclin-dependent kinase 2 associated protein	Hs.719107
213183 s at	1417649 at	up	3.183	up	2.577	up	2.159	up	3.458			1028	CDKN1C	Cyclin-dependent kinase inhibitor 1C (p57, Ki	Hs.106070
213348 at	1417649 at	up	3.536	up	2.577	up	2.159	up	3.458			1028	CDKN1C	cyclin-dependent kinase inhibitor 1C (p57, Kip	Hs.106070
213182 x at	1417649 at	up	3.569	up	2.577	up	2.159	up	3.458			1028	CDKN1C	cyclin-dependent kinase inhibitor 1C (p57, Kip	Hs.106070
219534 x at	1417649 at	up	3.647	up	2.577	up	2.159	up	3.458			1028	CDKN1C	cyclin-dependent kinase inhibitor 1C (p57, Kip	Hs.106070
216894 x at	1417649 at	up	3.849	up	2.577	up	2.159	up	3.458			1028	CDKN1C	cyclin-dependent kinase inhibitor 1C (p57, Kip	Hs.106070
209172 s at	1427161 at	up	1.163	up	1.724			up	1.537	up	1.564	1063	CENPF	centromere protein F, 350/400ka (mitosin)	Hs.497741
207828 s at	1427161 at	up	1.287	up	1.724			up	1.537	up	1.564	1063	CENPF	centromere protein F, 350/400ka (mitosin)	Hs.497741
239413 at	1427496 at	up	1.203	up	1.332	up	1.607	up	1.384			22995	CEP152	centrosomal protein 152kDa	Hs.597323
210416 s at	1422747 at	up	1.395	up	1.575	up	1.359	up	1.551	up	1.722	11200	CHEK2	CHK2 checkpoint homolog (S. pombe)	Hs.291363
206227 at	1457296 at	up	2.085	up	28.293	up	19.173	up	36.611	up	28.925	8483	CILP	cartilage intermediate layer protein, nucleotide	Hs.442180
204170 s at	1417457 at	up	1.211	up	1.784	up	1.581			up	1.676	1164	CKS2	CDC28 protein kinase regulatory subunit 2	Hs.83758
204170 s at	1417458 s at	up	1.211	up	1.681			up	1.525	up	1.687	1164	CKS2	CDC28 protein kinase regulatory subunit 2	Hs.83758
206165 s at	1438109 at	up	1.148	up	1.632	up	1.535	up	1.583			9635	CLCA2	chloride channel accessory 2	Hs.241551
205830 at	1418617 x at	up	1.327	up	2.021	up	1.763	up	1.895	up	1.733	1047	CLGN	calmegin	Hs.86368
205830 at	1457829 at	up	1.327					up	1.189			1047	CLGN	calmegin	Hs.86368
222874 s at	1448455 at	up	1.225					up	1.479	up	1.711	2055	CLN8	ceroid-lipofuscinosis, neuronal 8 (epilepsy, pr	Hs.127675
222874 s at	1448456 at	up	1.225	up	1.270							2055	CLN8	ceroid-lipofuscinosis, neuronal 8 (epilepsy, pr	Hs.127675
222874 s at	1459992 x at	up	1.225	up	1.439							2055	CLN8	ceroid-lipofuscinosis, neuronal 8 (epilepsy, pr	Hs.127675
218111 s at	1426662 at	up	1.306	up	1.602	up	1.279			up	1.581	55907	CMAS	cytidine monophosphate N-acetylneuraminic a	Hs.311346
230045 at	1435165 at	up	1.238	up	1.919	up	1.237	up	1.296	up	1.621	6900	CNTN2	contactin 2 (axonal)	Hs.519220
216865 at	1428455 at	down	1.297			down	2.258	down	1.778	down	1.710	7373	COL14A1	collagen, type XIV, alpha 1	Hs.409662
216865 at	1427168 a at	down	1.297	down	1.995	down	2.515					7373	COL14A1	collagen, type XIV, alpha 1	Hs.409662
202311 s at	1455494 at	up	2.106	up	3.250	up	4.231	up	4.923	up	5.090	1277	COL1A1	collagen, type I, alpha 1	Hs.172928
202310 s at	1455494 at	up	4.648	up	3.250	up	4.231	up	4.923	up	5.090	1277	COL1A1	collagen, type I, alpha 1	Hs.172928
1556499 s at	1455494 at	up	5.663	up	3.250	up	4.231	up	4.923	up	5.090	1277	COL1A1	collagen, type I, alpha 1	Hs.172928
213428 s at	1448590 at	up	1.327	up	2.190	up	1.444	up	1.504			1291	COL6A1	collagen, type VI, alpha 1	Hs.474053
204724 s at	1460693 a at	up	1.376	up	2.024			up	2.065	up	2.672	1299	COL9A3	collagen, type IX, alpha 3	Hs.716639
205713 s at	1419527 at	up	1.296	up	4.122	up	4.415	up	3.604	up	1.955	1311	COMP	cartilage oligomeric matrix protein	Hs.1584
213379 at	1452791 at	up	1.189	up	1.701			up	1.416	up	1.534	27235	COQ2	coenzyme Q2 homolog, prenyltransferase (ye	Hs.144304
206100 at	1429413 at	up	1.143	up	2.758			up	2.520	up	1.485	1368	CPM		Hs.654387
206100 at	1453009 at	up	1.143	up	2.980	up	1.567	up	1.565	up	1.533	1368	CPM		Hs.654387
225129 at	1424831 at	down	1.230			down	2.084	down	2.105	down	2.054	221184	CPNE2	copine II	Hs.339809
217564 s at	1455540 at	up	1.282	up	2.891	up	2.314	up	1.984	up	1.725	1373	CPS1	carbamoyl-phosphate synthetase 1, mitochon	Hs.149252
204920 at	1455540 at	up	1.298	up	2.891	up	2.314	up	1.984	up	1.725	1373	CPS1	carbamoyl-phosphate synthetase 1, mitochon	Hs.149252
221204 s at	1459147 at	down	1.361					down	1.189	down	1.254	55118	CRTAC1	cartilage acidic protein 1	Hs.500736
221204 s at	1459858 x at	down	1.361					down	1.250			55118	CRTAC1	cartilage acidic protein 1	Hs.500736
221204 s at	1452936 at	down	1.361	down	1.432							55118	CRTAC1	cartilage acidic protein 1	Hs.500736
209283 at	1416455 a at	up	1.503	up	1.542	up	1.848	up	1.560	up	1.578	1410	CRYAB	crystallin, alpha B	Hs.408767
209283 at	1434369 a at	up	1.503			up	1.919	up	1.625	up	1.949	1410	CRYAB	crystallin, alpha B	Hs.408767
209101 at	1416953 at	up	1.605	up	3.143	up	2.174	up	2.573	up	3.799	1490	CTGF	connective tissue growth factor	Hs.591346
206085 s at	1426243 at	up	1.118	up	3.129	up	3.421	up	2.868	up	3.358	1491	CTH	cystathionase (cystathionine gamma-lyase)	Hs.19904
209774 x at	1441855 x at	up	1.424	up	2.191					up	1.470	2920	CXCL2	chemokine (C-X-C motif) ligand 2	Hs.75765
209774 x at	1419209 at	up	1.424	up	1.916			up	1.228	up	1.501	2920	CXCL2	chemokine (C-X-C motif) ligand 2	Hs.75765
209774 x at	1457644 s at	up	1.424	up	2.171							2920	CXCL2	chemokine (C-X-C motif) ligand 2	Hs.75765
202436 s at	1416612 at	up	1.789	up	2.182	up	1.484	up	1.287			1545	CYP1B1	cytochrome P450, family 1, subfamily B, polyp	Hs.154654
202435 s at	1416612 at	up	1.873	up	2.182	up	1.484	up	1.287			1545	CYP1B1	cytochrome P450, family 1, subfamily B, polyp	Hs.154654
202437 s at	1416612 at	up	2.022	up	2.182	up	1.484	up	1.287			1545	CYP1B1	cytochrome P450, family 1, subfamily B, polyp	Hs.154654
202436 s at	1416613 at	up	1.789	up	1.661							1545	CYP1B1	cytochrome P450, family 1, subfamily B, polyp	Hs.154654
202435 s at	1416613 at	up	1.873	up	1.661							1545	CYP1B1	cytochrome P450, family 1, subfamily B, polyp	Hs.154654
202437 s at	1416613 at	up	2.022	up	1.661							1545	CYP1B1	cytochrome P450, family 1, subfamily B, polyp	Hs.154654
205417 s at	1426778 at	up	1.197	up	1.553	up	1.334	up	1.580	up	1.699	1605	DAG1	dystroglycan 1 (dystrophin-associated glycopr	Hs.76111
224911 s at	1437635 at	up	1.155			up	1.741	up	1.546	up	1.633	131566	DCBLD2	discoidin, CUB and LCCL domain containing	Hs.203691
224911 s at	1449891 a at	up	1.155	up	1.317			up	1.142			131566	DCBLD2	discoidin, CUB and LCCL domain containing	Hs.203691
209094 at	1454995 at	up	1.613	up	2.038	up	2.078	up	1.495	up	1.476	23576	DDAH1	dimethylarginine dimethylaminohydrolase 1	Hs.379858
243711 at	1454995 at	up	2.287	up	2.038	up	2.078	up	1.495	up	1.476	23576	DDAH1	dimethylarginine dimethylaminohydrolase 1	Hs.379858
209094 at	1429298 at	up	1.613	up	2.017	up	2.333	up	1.528	up	1.676	23576	DDAH1	dimethylarginine dimethylaminohydrolase 1	Hs.379858
243711 at	1429298 at	up	2.287	up	2.017	up	2.333	up	1.528	up	1.676	23576	DDAH1	dimethylarginine dimethylaminohydrolase 1	Hs.379858
242961 x at	1436562 at	up	3.119	up	2.114	up	1.645	up	2.101	up	2.250	23586	DDX58	DEAD (Asp-Glu-Ala-Asp) box polypeptide 58	Hs.190622
222793 at	1436562 at	up	3.688	up	2.114	up	1.645	up	2.101	up	2.250	23586	DDX58	DEAD (Asp-Glu-Ala-Asp) box polypeptide 58	Hs.190622
218943 s at	1436562 at	up	4.345	up	2.114	up	1.645	up	2.101	up	2.250	23586	DDX58	DEAD (Asp-Glu-Ala-Asp) box polypeptide 58	Hs.190622
242961 x at	1456890 at	up	3.119			up	1.315					23586	DDX58	DEAD (Asp-Glu-Ala-Asp) box polypeptide 58	Hs.190622
222793 at	1456890 at	up	3.688			up	1.315					23586	DDX58	DEAD (Asp-Glu-Ala-Asp) box polypeptide 58	Hs.190622
218943 s at	1456890 at	up	4.345			up	1.315					23586	DDX58	DEAD (Asp-Glu-Ala-Asp) box polypeptide 58	Hs.190622
218986 s at	1451777 at	up	2.438	up	4.589	up	3.050	up	3.939	up	4.752	55601	DDX60	DEAD (Asp-Glu-Ala-Asp) box polypeptide 60	Hs.591710
218986 s at	1439114 at	up	2.438	up	3.426			up	4.144	up	4.764	55601	DDX60	DEAD (Asp-Glu-Ala-Asp) box polypeptide 60	Hs.591710
238917 s at	1455340 at	up	1.082	up	2.585	up	1.948	up	2.171	up	2.256	160518	DENND5B	DENN/MADD domain containing 5B	Hs.118166
218858 at	1451348 at	up	1.746	up	3.015	up	2.115	up	1.665	up	1.694	64798	DEPDC6	DEP domain containing 6	Hs.112981
218858 at	1428622 at	up	1.746	up	1.598	up	1.646	up	1.781			64798	DEPDC6	DEP domain containing 6	Hs.112981
218858 at	1453571 at	up	1.746	up	1.645	up	1.616					64798	DEPDC6	DEP domain containing 6	Hs.112981
218858 at	1443579 s at	up	1.746	up	1.745	up	1.789					64798	DEPDC6	DEP domain containing 6	Hs.112981
209560 s at	1449939 s at	up	1.297	up	1.498			up	1.514	up	1.722	8788	DLK1	delta-like 1 homolog (Drosophila)	Hs.533717
224215 s at	1419204 at	up	1.744			up	1.260	up	1.239	up	1.247	28514	DLL1	delta-like 1 (Drosophila)	Hs.379912
213647 at	1457909 at	up	1.318					up	1.334	up	1.522	1763	DNA2	DNA replication helicase 2 homolog (yeast)	Hs.532446
213647 at	1452210 at	up	1.318	up	1.529			up	1.614	up	1.758	1763	DNA2	DNA replication helicase 2 homolog (yeast)	Hs.532446
242591 at	1434594 at	up	1.198			up	2.081	up	1.707	up	1.893	25981	DNAH1	Dynein, axonemal, heavy chain 1	Hs.655469
215840 at	1438763 at	up	1.163	up	3.166			up	2.363	up	2.936	146754	DNAH2	dynein, axonemal, heavy chain 2	Hs.367649
1554334 a at	1418591 at	up	1.490	up	1.767	up	1.531	up	1.509	up	1.532	55466	DNAJA4	DnaJ (Hsp40) homolog, subfamily A, member	Hs.513053
1554334 a at	1418592 at	up	1.490			up	1.278					55466	DNAJA4	DnaJ (Hsp40) homolog, subfamily A, member	Hs.513053
1554334 a at	1434196 at	up	1.490	up	1.655	up	1.623					55466	DNAJA4	DnaJ (Hsp40) homolog, subfamily A, member	Hs.513053
215116 s at	1460365 a at	up	1.346	up	1.443			up	1.581	up	1.563	1759	DNM1	dynamin 1	Hs.522413
220668 s at	1418351 a at	up	1.369	up	1.360	up	1.335			up	1.433	1789	DNMT3B	DNA (cytosine-5-)-methyltransferase 3 beta	Hs.643024
220668 s at	1449052 a at	up	1.369	up	1.589	up	1.480					1789	DNMT3B	DNA (cytosine-5-)-methyltransferase 3 beta	Hs.643024
215433 at	1433759 at	down	1.072					down	1.886	down	1.935	23333	DPY19L1	Dpy-19-like 1 (C. elegans)	Hs.408623
215433 at	1456533 at	down	1.072	down	1.336							23333	DPY19L1	Dpy-19-like 1 (C. elegans)	Hs.408623
225633 at	1455468 at	up	1.326	up	1.515	up	1.572	up	1.291			147991	DPY19L3	dpy-19-like 3 (C. elegans)	Hs.194392
225633 at	1437858 at	up	1.326			up	1.267					147991	DPY19L3	dpy-19-like 3 (C. elegans)	Hs.194392
1554536 at	1427945 at	down	1.204	down	3.880	down	1.421	down	1.820	down	2.451	1806	DPYD	dihydropyrimidine dehydrogenase	Hs.335034
204646 at	1427945 at	down	1.219	down	3.880	down	1.421	down	1.820	down	2.451	1806	DPYD	dihydropyrimidine dehydrogenase	Hs.335034
1554534 at	1427945 at	down	1.287	down	3.880	down	1.421	down	1.820	down	2.451	1806	DPYD	dihydropyrimidine dehydrogenase	Hs.335034
1554536 at	1427946 s at	down	1.204	down	3.530			down	1.963	down	2.636	1806	DPYD	dihydropyrimidine dehydrogenase	Hs.335034
204646 at	1427946 s at	down	1.219	down	3.530			down	1.963	down	2.636	1806	DPYD	dihydropyrimidine dehydrogenase	Hs.335034
1554534 at	1427946 s at	down	1.287	down	3.530			down	1.963	down	2.636	1806	DPYD	dihydropyrimidine dehydrogenase	Hs.335034
219000 s at	1452912 at	up	1.612	up	1.966	up	1.585	up	1.527			79075	DSCC1	defective in sister chromatid cohesion 1 homo	Hs.315167
218585 s at	1430228 at	up	1.276							up	1.466	51514	DTL	denticleless homolog (Drosophila)	Hs.656473
222680 s at	1430228 at	up	1.407							up	1.466	51514	DTL	denticleless homolog (Drosophila)	Hs.656473
218585 s at	1456652 at	up	1.276			up	1.448	up	1.392	up	1.533	51514	DTL	denticleless homolog (Drosophila)	Hs.656473
222680 s at	1456652 at	up	1.407			up	1.448	up	1.392	up	1.533	51514	DTL	denticleless homolog (Drosophila)	Hs.656473
218585 s at	1434695 at	up	1.276	up	1.818	up	1.999	up	1.929	up	2.119	51514	DTL	denticleless homolog (Drosophila)	Hs.656473
222680 s at	1434695 at	up	1.407	up	1.818	up	1.999	up	1.929	up	2.119	51514	DTL	denticleless homolog (Drosophila)	Hs.656473
225415 at	1439825 at	up	1.516			up	1.527	up	1.628	up	1.703	151636	DTX3L	deltex 3-like (Drosophila)	Hs.518201
224832 at	1440615 at	down	1.092			down	1.536	down	1.610	down	1.784	80824	DUSP16	dual specificity phosphatase 16	Hs.536535
224832 at	1418401 a at	down	1.092			down	1.462					80824	DUSP16	dual specificity phosphatase 16	Hs.536535
205777 at	1454737 at	up	1.601	up	1.541			up	1.230	up	1.600	1852	DUSP9	dual specificity phosphatase 9	Hs.719154
229106 at	1418258 s at	up	1.382					up	1.180	up	1.254	140735	DYNLL2	dynein, light chain, LC8-type 2	Hs.514368
229106 at	1418371 at	up	1.382			up	1.293					140735	DYNLL2	dynein, light chain, LC8-type 2	Hs.514368
207232 s at	1454878 at	up	1.264	up	1.599	up	1.445			up	1.782	9666	DZIP3	DAZ interacting protein 3, zinc finger	Hs.409210
213186 at	1454878 at	up	1.373	up	1.599	up	1.445			up	1.782	9666	DZIP3	DAZ interacting protein 3, zinc finger	Hs.409210
207231 at	1454878 at	up	1.530	up	1.599	up	1.445			up	1.782	9666	DZIP3	DAZ interacting protein 3, zinc finger	Hs.409210
203957 at	1448835 at	up	1.102	up	1.343			up	1.340	up	1.710	1876	E2F6	E2F transcription factor 6	Hs.603093
227242 s at	1452751 at	up	1.174	up	1.772	up	1.448			up	1.551	253738	EBF3	early B-cell factor 3	Hs.591374
209365 s at	1448613 at	up	1.803	up	3.465	up	2.100	up	1.900			1893	ECM1	extracellular matrix protein 1	Hs.81071
204211 x at	1422005 at	up	1.319	up	1.149					up	1.404	5610	EIF2AK2	eukaryotic translation initiation factor 2-alpha k	Hs.131431
204211 x at	1440866 at	up	1.319					up	1.599	up	1.711	5610	EIF2AK2	eukaryotic translation initiation factor 2-alpha k	Hs.131431
226099 at	1450744 at	down	1.241			down	1.345	down	1.373	down	1.537	22936	ELL2	elongation factor, RNA polymerase II, 2	Hs.192221
226982 at	1450744 at	down	1.281			down	1.345	down	1.373	down	1.537	22936	ELL2	elongation factor, RNA polymerase II, 2	Hs.192221

238500 at	1425272 at	up	1.192	up	1.815	up	1.637			up	1.523	2013	EMP2	epithelial membrane protein 2	Hs.531561
225078 at	1425272 at	up	1.295	up	1.815	up	1.637			up	1.523	2013	EMP2	epithelial membrane protein 2	Hs.531561
225079 at	1425272 at	up	1.305	up	1.815	up	1.637			up	1.523	2013	EMP2	epithelial membrane protein 2	Hs.531561
204975 at	1425272 at	up	1.557	up	1.815	up	1.637			up	1.523	2013	EMP2	epithelial membrane protein 2	Hs.531561
238500 at	1433670 at	up	1.192	up	2.528	up	1.749	up	1.441			2013	EMP2	epithelial membrane protein 2	Hs.531561
225078 at	1433670 at	up	1.295	up	2.528	up	1.749	up	1.441			2013	EMP2	epithelial membrane protein 2	Hs.531561
225079 at	1433670 at	up	1.305	up	2.528	up	1.749	up	1.441			2013	EMP2	epithelial membrane protein 2	Hs.531561
204975 at	1433670 at	up	1.557	up	2.528	up	1.749	up	1.441			2013	EMP2	epithelial membrane protein 2	Hs.531561
209392 at	1415894 at	up	1.324	up	4.215	up	1.768	up	2.855	up	2.771	5168	ENPP2	ectonucleotide pyrophosphatase/phosphodies	Hs.190977
209392 at	1448136 at	up	1.324	up	4.694	up	1.957	up	3.570	up	3.808	5168	ENPP2	ectonucleotide pyrophosphatase/phosphodies	Hs.190977
223253 at	1450380 at	up	1.466	up	1.402	up	1.172	up	1.423	up	1.655	54749	EPDR1	ependymin related protein 1 (zebrafish)	Hs.563491
223253 at	1460074 x at	up	1.466					up	1.389	up	1.785	54749	EPDR1	ependymin related protein 1 (zebrafish)	Hs.563491
217886 at	1419252 at	down	1.256	down	1.559	down	1.453	down	1.515	down	1.712	2060	EPS15	epidermal growth factor receptor pathway sub	Hs.83722
217887 s at	1419252 at	down	1.279	down	1.559	down	1.453	down	1.515	down	1.712	2060	EPS15	epidermal growth factor receptor pathway sub	Hs.83722
217886 at	1419251 at	down	1.256			down	1.868					2060	EPS15	epidermal growth factor receptor pathway sub	Hs.83722
217887 s at	1419251 at	down	1.279			down	1.868					2060	EPS15	epidermal growth factor receptor pathway sub	Hs.83722
205767 at	1419431 at	up	1.159	up	6.228	up	2.570	up	5.316	up	9.069	2069	EREG	epiregulin	Hs.115263
213365 at	1438172 x at	up	1.251			up	1.589	up	1.363	up	1.452	112479	ERI2	exoribonuclease 2	Hs.248437
222603 at	1433702 at	up	1.170	up	1.973	up	1.502			up	1.247	79956	ERMP1	endoplasmic reticulum metallopeptidase 1	Hs.591078
208394 x at	1449280 at	up	3.670	up	2.855	up	1.880	up	1.775	up	2.085	11082	ESM1	endothelial cell-specific molecule 1	Hs.129944
222262 s at	1439972 at	up	1.220			up	1.401	up	1.443	up	1.622	55500	ETNK1	ethanolamine kinase 1	Hs.29464
219017 at	1439972 at	up	1.262			up	1.401	up	1.443	up	1.622	55500	ETNK1	ethanolamine kinase 1	Hs.29464
204603 at	1418026 at	up	1.524	up	1.469			up	1.294	up	1.662	9156	EXO1	exonuclease 1	Hs.498248
226259 at	1424231 s at	up	1.158					up	1.152	up	1.179	54536	EXOC6	exocyst complex component 6	Hs.655657
226259 at	1460576 at	up	1.158	up	1.622	up	1.405	up	1.329	up	1.328	54536	EXOC6	exocyst complex component 6	Hs.655657
240834 at	1427966 at	down	1.326			down	1.681	down	1.776	down	1.749	90268	FAM105B	family with sequence similarity 105, member E	Hs.406335
240834 at	1443099 at	down	1.326	down	1.645	down	1.523					90268	FAM105B	family with sequence similarity 105, member E	Hs.406335
223058 at	1416893 at	down	1.432	down	1.640	down	1.365	down	1.678	down	1.572	83641	FAM107B	family with sequence similarity 107, member E	Hs.446315
223058 at	1416892 s at	down	1.432	down	1.738	down	1.336	down	1.697	down	1.695	83641	FAM107B	family with sequence similarity 107, member E	Hs.446315
223058 at	1448509 at	down	1.432					down	2.078	down	1.961	83641	FAM107B	family with sequence similarity 107, member E	Hs.446315
214890 s at	1437950 at	up	1.172			up	1.685	up	1.419	up	1.386	25854	FAM149A	family with sequence similarity 149, member A	Hs.357025
214890 s at	1455793 at	up	1.172	up	1.688			up	1.677	up	1.806	25854	FAM149A	family with sequence similarity 149, member A	Hs.357025
227410 at	1426734 at	up	1.830	up	1.823			up	1.363	up	1.430	131583	FAM43A	family with sequence similarity 43, member A	Hs.708232
218898 at	1435076 at	up	1.058			up	1.396	up	1.367	up	1.486	79850	FAM57A	family with sequence similarity 57, member A	Hs.154396
1568891 x at	1442092 at	up	1.292					up	1.263	up	1.300	2177	FANCD2	Fanconi anemia, complementation group D2	Hs.208388
242560 at	1442092 at	up	1.472					up	1.263	up	1.300	2177	FANCD2	Fanconi anemia, complementation group D2	Hs.208388
1568891 x at	1439091 at	up	1.292	up	1.500	up	1.618	up	1.294	up	1.350	2177	FANCD2	Fanconi anemia, complementation group D2	Hs.208388
242560 at	1439091 at	up	1.472	up	1.500	up	1.618	up	1.294	up	1.350	2177	FANCD2	Fanconi anemia, complementation group D2	Hs.208388
1568891 x at	1438339 at	up	1.292	up	2.160	up	1.706					2177	FANCD2	Fanconi anemia, complementation group D2	Hs.208388
242560 at	1438339 at	up	1.472	up	2.160	up	1.706					2177	FANCD2	Fanconi anemia, complementation group D2	Hs.208388
221345 at	1425216 at	up	1.191			up	1.609	up	1.403	up	3.330	2867	FFAR2	free fatty acid receptor 2	Hs.248056
221345 at	1425215 at	up	1.191			up	1.269					2867	FFAR2	free fatty acid receptor 2	Hs.248056
208240 s at	1450869 at	up	1.242			up	1.305	up	1.141	up	1.162	2246	FGF1	fibroblast growth factor 1 (acidic)	Hs.483635
1552721 a at	1450869 at	up	1.256			up	1.305	up	1.141	up	1.162	2246	FGF1	fibroblast growth factor 1 (acidic)	Hs.483635
208240 s at	1423136 at	up	1.242			up	1.690			up	1.307	2246	FGF1	fibroblast growth factor 1 (acidic)	Hs.483635
1552721 a at	1423136 at	up	1.256			up	1.690			up	1.307	2246	FGF1	fibroblast growth factor 1 (acidic)	Hs.483635
207501 s at	1440270 at	up	1.221	up	1.832			up	1.446	up	1.486	2257	FGF12	fibroblast growth factor 12	Hs.584758
214170 x at	1424828 a at	up	1.049	up	1.477			up	1.445	up	1.599	2271	FH	fumarate hydratase	Hs.592490
212169 at	1437687 x at	up	1.106			up	1.336	up	1.518	up	1.737	11328	FKBP9	FK506 binding protein 9, 63 kDa	Hs.103934
212169 at	1423677 at	up	1.106			up	1.422			up	1.797	11328	FKBP9	FK506 binding protein 9, 63 kDa	Hs.103934
210316 at	1444052 at	up	1.436			up	1.533	up	1.561	up	1.697	2324	FLT4	FMS-like tyrosine kinase 4	Hs.646917
223936 s at	1421142 s at	up	1.215					up	1.248	up	1.240	27086	FOXP1	forkhead box P1	Hs.431498
223936 s at	1421141 a at	up	1.215					up	1.126			27086	FOXP1	forkhead box P1	Hs.431498
223936 s at	1438802 at	up	1.215	up	1.397	up	1.302					27086	FOXP1	forkhead box P1	Hs.431498
223936 s at	1435221 at	up	1.215			up	1.314					27086	FOXP1	forkhead box P1	Hs.431498
223936 s at	1435222 at	up	1.215			up	1.629					27086	FOXP1	forkhead box P1	Hs.431498
223936 s at	1443258 at	up	1.215	up	1.122							27086	FOXP1	forkhead box P1	Hs.431498
223936 s at	1455242 at	up	1.215	up	1.155							27086	FOXP1	forkhead box P1	Hs.431498
223936 s at	1421140 a at	up	1.215	up	1.530							27086	FOXP1	forkhead box P1	Hs.431498
213524 s at	1448700 at	up	1.204	up	2.428	up	1.478	up	2.906			50486	G0S2	G0/G1switch 2	Hs.432132
208868 s at	1416418 at	up	1.792	up	1.654	up	1.571	up	1.823	up	1.771	23710	GABARAPL1	GABA(A) receptor-associated protein like 1	Hs.524250
208869 s at	1416418 at	up	2.440	up	1.654	up	1.571	up	1.823	up	1.771	23710	GABARAPL1	GABA(A) receptor-associated protein like 1	Hs.524250
208868 s at	1416419 s at	up	1.792	up	2.181	up	1.514	up	1.790	up	1.835	23710	GABARAPL1	GABA(A) receptor-associated protein like 1	Hs.524250
208869 s at	1416419 s at	up	2.440	up	2.181	up	1.514	up	1.790	up	1.835	23710	GABARAPL1	GABA(A) receptor-associated protein like 1	Hs.524250
211458 s at	1416418 at	up	2.591	up	1.654	up	1.571	up	1.823	up	1.771	23710 /// 23766	GABARAPL1 //	GABA(A) receptor-associated protein like 1 //	Hs.524250
211458 s at	1416419 s at	up	2.591	up	2.181	up	1.514	up	1.790	up	1.835	23710 /// 23766	GABARAPL1 //	GABA(A) receptor-associated protein like 1 //	Hs.524250
205890 s at	1455021 at	up	1.278	up	2.322	up	1.636	up	2.264	up	2.204	10537 /// 2550	GABBR1 /// UB	gamma-aminobutyric acid (GABA) B receptor	Hs.719331
205890 s at	1425595 at	up	1.278	up	1.519			up	1.361			10537 /// 2550	GABBR1 /// UB	gamma-aminobutyric acid (GABA) B receptor	Hs.719331
229724 at	1435021 at	up	1.103			up	1.646	up	1.313	up	2.541	2562	GABRB3	gamma-aminobutyric acid (GABA) A receptor	Hs.302352
229724 at	1421190 at	up	1.103					up	1.137			2562	GABRB3	gamma-aminobutyric acid (GABA) A receptor	Hs.302352
218313 s at	1452232 at	down	1.269			down	2.085	down	1.723	down	1.835	51809	GALNT7	UDP-N-acetyl-alpha-D-galactosamine:polyper	Hs.548088
218313 s at	1425581 s at	down	1.269	down	1.931	down	1.793					51809	GALNT7	UDP-N-acetyl-alpha-D-galactosamine:polyper	Hs.548088
218313 s at	1426908 at	down	1.269	down	2.290	down	2.035					51809	GALNT7	UDP-N-acetyl-alpha-D-galactosamine:polyper	Hs.548088
213280 at	1434754 at	up	1.247	up	1.844	up	1.760	up	1.229			23108	GARNL4	GTPase activating Rap/RanGAP domain-like	Hs.499659
202191 s at	1445685 at	down	1.146	down	1.265					down	1.267	8522	GAS7	growth arrest-specific 7	Hs.462214
202191 s at	1457270 at	down	1.146			down	1.845	down	2.611	down	2.755	8522	GAS7	growth arrest-specific 7	Hs.462214
202191 s at	1417859 at	down	1.146			down	1.577					8522	GAS7	growth arrest-specific 7	Hs.462214
209710 at	1428816 a at	up	1.471	up	2.695	up	2.504	up	2.578			2624	GATA2	GATA binding protein 2	Hs.367725
209710 at	1450333 a at	up	1.471	up	1.397							2624	GATA2	GATA binding protein 2	Hs.367725
235175 at	1418392 a at	up	2.113	up	3.986	up	2.502	up	2.817	up	2.845	115361	GBP4	guanylate binding protein 4	Hs.409925
219539 at	1424300 at	up	1.739	up	1.305	up	1.390			up	1.547	79833	GEMIN6	gem (nuclear organelle) associated protein 6	Hs.143818
200681 at	1424109 a at	up	1.101	up	1.588	up	1.610	up	1.442	up	1.392	2739	GLO1	glyoxalase I	Hs.268849
200681 at	1424108 at	up	1.101	up	1.869	up	1.615	up	1.305			2739	GLO1	glyoxalase I	Hs.268849
200681 at	1451240 a at	up	1.101	up	2.148	up	1.636	up	1.316			2739	GLO1	glyoxalase I	Hs.268849
212959 s at	1435335 a at	up	1.340			up	1.852	up	1.557	up	1.829	79158	GNPTAB	N-acetylglucosamine-1-phosphate transferase	Hs.46850
200708 at	1417715 a at	up	1.117	up	1.377			up	1.326	up	1.468	2806	GOT2	glutamic-oxaloacetic transaminase 2, mitocho	Hs.599470
214324 at	1449452 a at	up	1.101	up	4.623	up	5.977	up	3.757			2813	GP2	glycoprotein 2 (zymogen granule membrane)	Hs.53985
225463 x at	1424696 at	up	1.236	up	1.443			up	1.322	up	1.455	653519	GPR89A	G protein-coupled receptor 89A	Hs.559115
220642 x at	1424696 at	up	1.173	up	1.443			up	1.322	up	1.455	3 /// 653519 /// 7	GPR89A /// GP	G protein-coupled receptor 89A /// G protein-c	Hs.559115
223531 x at	1424696 at	up	1.228	up	1.443			up	1.322	up	1.455	3 /// 653519 /// 7	GPR89A /// GP	G protein-coupled receptor 89A /// G protein-c	Hs.559115
222140 s at	1424696 at	up	1.257	up	1.443			up	1.322	up	1.455	3 /// 653519 /// 7	GPR89A /// GP	G protein-coupled receptor 89A /// G protein-c	Hs.559115
227628 at	1424099 at	up	1.218	up	1.477	up	1.562	up	1.292	up	1.737	493869	GPX8	glutathione peroxidase 8 (putative)	Hs.289044
206204 at	1417673 at	up	1.195	up	3.388	up	2.994	up	2.577	up	3.144	2888	GRB14	growth factor receptor-bound protein 14	Hs.411881
205862 at	1439568 at	up	1.901	up	1.575	up	1.507	up	1.258	up	1.600	9687	GREB1	GREB1 protein	Hs.467733
202554 s at	1416842 at	up	1.347	up	4.325	up	2.786	up	1.765	up	2.418	2947	GSTM3	glutathione S-transferase mu 3 (brain)	Hs.2006
210912 x at	1424835 at	up	1.098	up	1.737	up	1.298	up	1.400	up	1.528	2948	GSTM4	glutathione S-transferase mu 4	Hs.348387
243228 at	1434320 at	up	1.138	up	1.570	up	1.557			up	1.649	9329	GTF3C4	General transcription factor IIIC, polypeptide 4	22302 /// Hs.656
227711 at	1441836 x at	up	7.196	up	1.979	up	1.572	up	2.583	up	2.703	121355	GTSF1	gametocyte specific factor 1	Hs.524476
211555 s at	1420872 at	up	1.209	up	2.743			up	3.039	up	3.138	2983	GUCY1B3	guanylate cyclase 1, soluble, beta 3	Hs.77890
203817 at	1420872 at	up	1.430	up	2.743			up	3.039	up	3.138	2983	GUCY1B3	guanylate cyclase 1, soluble, beta 3	Hs.77890
214414 x at	1417714 x at	up	1.385	up	1.520	up	1.924	up	1.268	up	1.303	3039 /// 3040	HBA1 /// HBA2	hemoglobin, alpha 1 /// hemoglobin, alpha 2	Hs.449630
214414 x at	1428361 x at	up	1.385	up	1.631	up	1.887	up	1.167	up	1.554	3039 /// 3040	HBA1 /// HBA2	hemoglobin, alpha 1 /// hemoglobin, alpha 2	Hs.449630
214414 x at	1452757 s at	up	1.385	up	1.324	up	1.575					3039 /// 3040	HBA1 /// HBA2	hemoglobin, alpha 1 /// hemoglobin, alpha 2	Hs.449630
205919 at	1450621 a at	up	1.318	up	4.195	up	3.263	up	3.716	up	5.467	3046	HBE1	hemoglobin, epsilon 1	Hs.655195
205919 at	1436717 x at	up	1.318	up	50.685	up	43.684	up	67.836	up	85.536	3046	HBE1	hemoglobin, epsilon 1	Hs.655195
205919 at	1436823 x at	up	1.318	up	53.080	up	42.445	up	66.658	up	91.790	3046	HBE1	hemoglobin, epsilon 1	Hs.655195
203821 at	1418349 at	up	1.108	up	1.498			up	1.630	up	1.449	1839	HBEGF	heparin-binding EGF-like growth factor	Hs.799
203821 at	1418350 at	up	1.108					up	1.837	up	1.680	1839	HBEGF	heparin-binding EGF-like growth factor	Hs.799
213515 x at	1437990 x at	up	1.736	up	2.935	up	2.251	up	2.249	up	2.614	3047 /// 3048	HBG1 /// HBG2	hemoglobin, gamma A /// hemoglobin, gamma	Hs.712539
213515 x at	1437810 a at	up	1.736	up	1.641	up	1.900					3047 /// 3048	HBG1 /// HBG2	hemoglobin, gamma A /// hemoglobin, gamma	Hs.712539
229406 at	1436450 at	up	1.554			up	2.302	up	2.053	up	2.183	146713	hCG 1776007	hexaribonucleotide binding protein 3	Hs.135229
235468 at	1436450 at	up	1.623			up	2.302	up	2.053	up	2.183	146713	hCG 1776007	hexaribonucleotide binding protein 3	Hs.135229
206313 at	1419297 at	down	1.115			down	1.655	down	1.528	down	1.726	3111	HLA-DOA	major histocompatibility complex, class II, DO	Hs.631991
211654 x at	1425477 x at	down	1.192			down	1.306	down	1.294	down	1.280	3119	HLA-DQB1	major histocompatibility complex, class II, DQ	Hs.409934
211654 x at	1450648 s at	down	1.192			down	1.286	down	1.241	down	1.317	3119	HLA-DQB1	major histocompatibility complex, class II, DQ	Hs.409934
211654 x at	1451721 a at	down	1.192					down	1.271			3119	HLA-DQB1	major histocompatibility complex, class II, DQ	Hs.409934
210982 s at	1422891 at	down	1.217	down	1.218	down	1.441	down	1.241			3122	HLA-DRA	major histocompatibility complex, class II, DR	Hs.520048
209709 s at	1427541 x at	up	1.370	up	1.376			up	1.482	up	1.710	3161	HMMR	hyaluronan-mediated motility receptor (RHAM	Hs.72550
207165 at	1427541 x at	up	1.384	up	1.376			up	1.482	up	1.710	3161	HMMR	hyaluronan-mediated motility receptor (RHAM	Hs.72550
209709 s at	1450156 a at	up	1.370					up	1.665	up	1.913	3161	HMMR	hyaluronan-mediated motility receptor (RHAM	Hs.72550
207165 at	1450156 a at	up	1.384					up	1.665	up	1.913	3161	HMMR	hyaluronan-mediated motility receptor (RHAM	Hs.72550
201277 s at	1415914 at	up	1.261	up	1.255			up	1.264	up	1.409	3182	HNRNPAB	heterogeneous nuclear ribonucleoprotein A/B	Hs.591731
201277 s at	1431349 at	up	1.261					up	1.336	up	1.458	3182	HNRNPAB	heterogeneous nuclear ribonucleoprotein A/B	Hs.591731
201277 s at	1453849 s at	up	1.261							up	1.585	3182	HNRNPAB	heterogeneous nuclear ribonucleoprotein A/B	Hs.591731
201277 s at	1448144 at	up	1.261					up	1.393	up	1.709	3182	HNRNPAB	heterogeneous nuclear ribonucleoprotein A/B	Hs.591731
201277 s at	1455855 x at	up	1.261	up	1.239							3182	HNRNPAB	heterogeneous nuclear ribonucleoprotein A/B	Hs.591731
214328 s at	1438902 a at	up	1.259			up	1.235	up	1.170	up	1.196	3320	HSP90AA1	heat shock protein 90kDa alpha (cytosolic), cl	Hs.525600
210211 s at	1438902 a at	up	1.267			up	1.235	up	1.170	up	1.196	3320	HSP90AA1	heat shock protein 90kDa alpha (cytosolic), cl	Hs.525600
214328 s at	1437497 a at	up	1.259			up	1.567			up	1.607	3320	HSP90AA1	heat shock protein 90kDa alpha (cytosolic), cl	Hs.525600
210211 s at	1437497 a at	up	1.267			up	1.567			up	1.607	3320	HSP90AA1	heat shock protein 90kDa alpha (cytosolic), cl	Hs.525600
214328 s at	1426645 at	up	1.259			up	1.872			up	1.717	3320	HSP90AA1	heat shock protein 90kDa alpha (cytosolic), cl	Hs.525600
210211 s at	1426645 at	up	1.267			up	1.872			up	1.717	3320	HSP90AA1	heat shock protein 90kDa alpha (cytosolic), cl	Hs.525600
214328 s at	1441827 x at	up	1.259			up	1.499					3320	HSP90AA1	heat shock protein 90kDa alpha (cytosolic), cl	Hs.525600
210211 s at	1441827 x at	up	1.267			up	1.499					3320	HSP90AA1	heat shock protein 90kDa alpha (cytosolic), cl	Hs.525600
207062 at	1437323 a at	up	1.424	up	7.159	up	5.340	up	7.919	up	7.077	3375	IAPP	islet amyloid polypeptide	Hs.46835
207062 at	1456004 x at	up	1.424	up	6.628	up	5.887	up	8.177	up	22.872	3375	IAPP	islet amyloid polypeptide	Hs.46835
207062 at	1423510 at	up	1.424	up	7.892	up	6.961	up	5.624			3375	IAPP	islet amyloid polypeptide	Hs.46835
207062 at	1423509 a at	up	1.424	up	6.752	up	5.216	up	12.271			3375	IAPP	islet amyloid polypeptide	Hs.46835
207826 s at	1416630 at	up	1.373	up	2.135			up	1.651	up	1.800	3399	ID3	inhibitor of DNA binding 3, dominant negative	Hs.76884
212221 x at	1434751 at	up	1.119	up	1.420			up	1.285			3423	IDS	iduronate 2-sulfatase	Hs.460960
206342 x at	1434751 at	up	1.287	up	1.420			up	1.285			3423	IDS	iduronate 2-sulfatase	Hs.460960
202439 s at	1434751 at	up	1.338	up	1.420			up	1.285			3423	IDS	iduronate 2-sulfatase	Hs.460960

202438 x at	1434751 at	up	1.351	up	1.420			up	1.285			3423	IDS	iduronate 2-sulfatase	Hs.460960
212221 x at	1455481 at	up	1.119			up	1.278					3423	IDS	iduronate 2-sulfatase	Hs.460960
206342 x at	1455481 at	up	1.287			up	1.278					3423	IDS	iduronate 2-sulfatase	Hs.460960
202439 s at	1455481 at	up	1.338			up	1.278					3423	IDS	iduronate 2-sulfatase	Hs.460960
202438 x at	1455481 at	up	1.351			up	1.278					3423	IDS	iduronate 2-sulfatase	Hs.460960
202411 at	1452956 a at	up	4.847			up	1.419	up	1.320	up	1.418	3429	IFI27	interferon, alpha-inducible protein 27	Hs.532634
202411 at	1454757 s at	up	4.847	up	1.810	up	1.501	up	1.421	up	1.619	3429	IFI27	interferon, alpha-inducible protein 27	Hs.532634
214059 at	1423555 a at	up	1.774	up	3.783	up	2.222	up	3.121	up	4.075	10561	IFI44	Interferon-induced protein 44	Hs.82316
214453 s at	1423555 a at	up	4.457	up	3.783	up	2.222	up	3.121	up	4.075	10561	IFI44	interferon-induced protein 44	Hs.82316
1555464 at	1426276 at	up	1.532	up	1.980	up	1.406	up	1.849	up	1.894	64135	IFIH1	interferon induced with helicase C domain 1	Hs.163173
216020 at	1426276 at	up	1.685	up	1.980	up	1.406	up	1.849	up	1.894	64135	IFIH1	Interferon induced with helicase C domain 1	Hs.163173
219209 at	1426276 at	up	2.036	up	1.980	up	1.406	up	1.849	up	1.894	64135	IFIH1	interferon induced with helicase C domain 1	Hs.163173
203153 at	1435529 at	up	6.224	up	22.074	up	8.523	up	22.330	up	11.862	3434	IFIT1	interferon-induced protein with tetratricopeptid	Hs.20315
203153 at	1457976 at	up	6.224	up	2.581	up	3.243	up	2.663			3434	IFIT1	interferon-induced protein with tetratricopeptid	Hs.20315
226757 at	1418293 at	up	4.046	up	4.435	up	2.289	up	6.574	up	6.828	3433	IFIT2	interferon-induced protein with tetratricopeptid	Hs.437609
204747 at	1449025 at	up	3.750	up	5.389	up	3.326	up	5.530	up	13.963	3437	IFIT3	interferon-induced protein with tetratricopeptid	Hs.714337
229450 at	1449025 at	up	4.355	up	5.389	up	3.326	up	5.530	up	13.963	3437	IFIT3	interferon-induced protein with tetratricopeptid	Hs.714337
202410 x at	1448152 at	up	1.395	up	2.550	up	2.617	up	1.761	up	1.511	3481 /// 723961	IGF2 /// INS-IG	insulin-like growth factor 2 (somatomedin A) /	Hs.272259
205302 at	1418918 at	up	1.392	up	3.232	up	3.814	up	3.314	up	3.436	3484	IGFBP1	insulin-like growth factor binding protein 1	Hs.642938
210095 s at	1458268 s at	up	2.465	up	3.086	up	1.482	up	1.738	up	1.770	3486	IGFBP3	insulin-like growth factor binding protein 3	Hs.450230
212143 s at	1458268 s at	up	2.496	up	3.086	up	1.482	up	1.738	up	1.770	3486	IGFBP3	insulin-like growth factor binding protein 3	Hs.450230
210095 s at	1423062 at	up	2.465	up	9.189	up	3.434	up	3.033	up	4.124	3486	IGFBP3	insulin-like growth factor binding protein 3	Hs.450230
212143 s at	1423062 at	up	2.496	up	9.189	up	3.434	up	3.033	up	4.124	3486	IGFBP3	insulin-like growth factor binding protein 3	Hs.450230
203426 s at	1422313 a at	down	1.405	down	1.353			down	1.164	down	1.253	3488	IGFBP5	insulin-like growth factor binding protein 5	Hs.607212
203851 at	1417933 at	up	1.839	up	4.558	up	2.851	up	3.689	up	3.546	3489	IGFBP6	insulin-like growth factor binding protein 6	Hs.274313
219971 at	1450456 at	down	1.930			down	2.110	down	2.024	down	2.032	50615	IL21R	interleukin 21 receptor	Hs.210546
212196 at	1452843 at	up	1.353	up	1.984	up	1.555	up	1.855	up	1.949	3572	IL6ST	interleukin 6 signal transducer (gp130, oncost	Hs.532082
212196 at	1437303 at	up	1.353							up	2.963	3572	IL6ST	interleukin 6 signal transducer (gp130, oncost	Hs.532082
222654 at	1437288 at	up	1.240			up	1.560			up	1.334	54928	IMPAD1	inositol monophosphatase domain containing	Hs.716471
218516 s at	1437288 at	up	1.299			up	1.560			up	1.334	54928	IMPAD1	inositol monophosphatase domain containing	Hs.716471
224744 at	1437288 at	up	1.309			up	1.560			up	1.334	54928	IMPAD1	inositol monophosphatase domain containing	Hs.716471
222654 at	1437290 at	up	1.240			up	1.570	up	1.250			54928	IMPAD1	inositol monophosphatase domain containing	Hs.716471
218516 s at	1437290 at	up	1.299			up	1.570	up	1.250			54928	IMPAD1	inositol monophosphatase domain containing	Hs.716471
224744 at	1437290 at	up	1.309			up	1.570	up	1.250			54928	IMPAD1	inositol monophosphatase domain containing	Hs.716471
222654 at	1437289 at	up	1.240			up	1.626					54928	IMPAD1	inositol monophosphatase domain containing	Hs.716471
218516 s at	1437289 at	up	1.299			up	1.626					54928	IMPAD1	inositol monophosphatase domain containing	Hs.716471
224744 at	1437289 at	up	1.309			up	1.626					54928	IMPAD1	inositol monophosphatase domain containing	Hs.716471
222659 at	1428096 at	up	1.195	up	1.213					up	1.497	51194	IPO11	importin 11	Hs.482269
222659 at	1429717 at	up	1.195			up	1.303					51194	IPO11	importin 11	Hs.482269
217885 at	1439481 at	up	1.142			up	1.312			up	1.689	55705	IPO9	importin 9	Hs.596014
217885 at	1441645 s at	up	1.142	up	1.386			up	1.340			55705	IPO9	importin 9	Hs.596014
203906 at	1452327 at	down	1.269	down	1.510	down	1.447	down	1.634	down	1.756	9922	IQSEC1	IQ motif and Sec7 domain 1	Hs.475506
203906 at	1427145 at	down	1.269					down	1.236			9922	IQSEC1	IQ motif and Sec7 domain 1	Hs.475506
224570 s at	1433633 at	down	1.410	down	1.581			down	1.202	down	1.248	359948	IRF2BP2	interferon regulatory factor 2 binding protein 2	Hs.693837
224569 s at	1433633 at	down	1.536	down	1.581			down	1.202	down	1.248	359948	IRF2BP2	interferon regulatory factor 2 binding protein 2	Hs.693837
224571 at	1433633 at	down	1.783	down	1.581			down	1.202	down	1.248	359948	IRF2BP2	interferon regulatory factor 2 binding protein 2	Hs.693837
224570 s at	1433634 at	down	1.410	down	1.283							359948	IRF2BP2	interferon regulatory factor 2 binding protein 2	Hs.693837
224569 s at	1433634 at	down	1.536	down	1.283							359948	IRF2BP2	interferon regulatory factor 2 binding protein 2	Hs.693837
224571 at	1433634 at	down	1.783	down	1.283							359948	IRF2BP2	interferon regulatory factor 2 binding protein 2	Hs.693837
208436 s at	1417244 a at	up	1.697	up	2.241	up	1.881	up	2.837	up	2.748	3665	IRF7	interferon regulatory factor 7	Hs.166120
205483 s at	1431591 s at	up	3.880			up	1.840	up	3.192	up	3.259	9636	ISG15	ISG15 ubiquitin-like modifier	Hs.458485
206104 at	1450723 at	up	1.431	up	2.317			up	1.722	up	1.602	3670	ISL1	ISL LIM homeobox 1	Hs.505
206104 at	1422720 at	up	1.431			up	1.230	up	1.445			3670	ISL1	ISL LIM homeobox 1	Hs.505
205755 at	1449123 at	up	1.632	up	12.002	up	8.167	up	10.098	up	24.253	3699	ITIH3	inter-alpha (globulin) inhibitor H3	Hs.76716
203710 at	1460203 at	up	1.257	up	1.666			up	1.722	up	1.737	3708	ITPR1	inositol 1,4,5-triphosphate receptor, type 1	Hs.567295
203710 at	1417279 at	up	1.257					up	1.369			3708	ITPR1	inositol 1,4,5-triphosphate receptor, type 1	Hs.567295
205889 s at	1436552 at	up	1.232	up	2.256			up	2.194	up	2.210	9832	JAKMIP2	Janus kinase and microtubule interacting prot	Hs.719177
227750 at	1436066 at	up	1.352			up	1.801	up	1.464	up	1.495	8997	KALRN	kalirin, RhoGEF kinase	Hs.8004
227750 at	1448023 at	up	1.352	up	1.466	up	1.504	up	1.631	up	1.499	8997	KALRN	kalirin, RhoGEF kinase	Hs.8004
227750 at	1429796 at	up	1.352			up	1.211					8997	KALRN	kalirin, RhoGEF kinase	Hs.8004
227750 at	1442341 at	up	1.352			up	1.223					8997	KALRN	kalirin, RhoGEF kinase	Hs.8004
213832 at	1435720 at	up	1.307	up	1.965	up	1.504	up	1.429	up	1.711	3752	KCND3	potassium voltage-gated channel, Shal-relatec	Hs.666367
220294 at	1453273 at	up	1.436			up	1.434	up	1.308	up	1.294	27012	KCNV1	potassium channel, subfamily V, member 1	Hs.13285
201549 x at	1427143 at	up	1.180	up	1.558	up	1.727	up	1.388	up	1.636	10765	KDM5B	lysine (K)-specific demethylase 5B	Hs.443650
211202 s at	1427143 at	up	1.184	up	1.558	up	1.727	up	1.388	up	1.636	10765	KDM5B	lysine (K)-specific demethylase 5B	Hs.443650
201548 s at	1427143 at	up	1.220	up	1.558	up	1.727	up	1.388	up	1.636	10765	KDM5B	lysine (K)-specific demethylase 5B	Hs.443650
201549 x at	1427142 s at	up	1.180			up	1.149					10765	KDM5B	lysine (K)-specific demethylase 5B	Hs.443650
211202 s at	1427142 s at	up	1.184			up	1.149					10765	KDM5B	lysine (K)-specific demethylase 5B	Hs.443650
201548 s at	1427142 s at	up	1.220			up	1.149					10765	KDM5B	lysine (K)-specific demethylase 5B	Hs.443650
201549 x at	1457482 at	up	1.180			up	1.212					10765	KDM5B	lysine (K)-specific demethylase 5B	Hs.443650
211202 s at	1457482 at	up	1.184			up	1.212					10765	KDM5B	lysine (K)-specific demethylase 5B	Hs.443650
201548 s at	1457482 at	up	1.220			up	1.212					10765	KDM5B	lysine (K)-specific demethylase 5B	Hs.443650
200851 s at	1423837 at	up	1.077					up	1.275	up	1.258	9798	KIAA0174	KIAA0174	Hs.232194
200851 s at	1423838 s at	up	1.077	up	1.504			up	1.314	up	1.386	9798	KIAA0174	KIAA0174	Hs.232194
225623 at	1424913 at	up	1.118							up	1.293	85457	KIAA1737	KIAA1737	
225623 at	1424914 at	up	1.118					up	1.229			85457	KIAA1737	KIAA1737	
225623 at	1424915 s at	up	1.118	up	1.705			up	1.700			85457	KIAA1737	KIAA1737	
204444 at	1452314 at	up	1.586	up	1.585			up	1.562	up	1.516	3832	KIF11	kinesin family member 11	Hs.8878
204444 at	1435306 a at	up	1.586	up	1.770	up	1.840			up	2.121	3832	KIF11	kinesin family member 11	Hs.8878
205235 s at	1449612 x at	up	1.601	up	1.798			up	1.471	up	1.691	9585	KIF20B	kinesin family member 20B	Hs.240
205235 s at	1439695 a at	up	1.601	up	1.792			up	1.369			9585	KIF20B	kinesin family member 20B	Hs.240
203087 s at	1450053 at	up	1.126	up	1.350	up	1.553	up	1.382	up	1.486	3796	KIF2A	kinesin heavy chain member 2A	Hs.558351
203087 s at	1450052 at	up	1.126			up	1.292					3796	KIF2A	kinesin heavy chain member 2A	Hs.558351
225140 at	1429360 at	up	1.116	up	1.814			up	1.424	up	1.359	51274	KLF3	Kruppel-like factor 3 (basic)	Hs.298658
225140 at	1431630 a at	up	1.116					up	1.113			51274	KLF3	Kruppel-like factor 3 (basic)	Hs.298658
225140 at	1441200 at	up	1.116	up	1.125							51274	KLF3	Kruppel-like factor 3 (basic)	Hs.298658
209254 at	1433997 at	up	1.253	up	1.277	up	1.340	up	1.162	up	1.171	23008	KLHDC10	kelch domain containing 10	Hs.520710
209254 at	1428771 at	up	1.253			up	1.414	up	1.422	up	1.477	23008	KLHDC10	kelch domain containing 10	Hs.520710
209854 s at	1450222 x at	up	1.138	up	4.539	up	1.900	up	2.602	up	2.403	3817	KLK2	kallikrein-related peptidase 2	Hs.515560
209854 s at	1420770 at	up	1.138	up	2.723	up	1.735	up	3.516	up	2.632	3817	KLK2	kallikrein-related peptidase 2	Hs.515560
209854 s at	1427380 at	up	1.138	up	3.598	up	1.721	up	3.119	up	2.675	3817	KLK2	kallikrein-related peptidase 2	Hs.515560
209854 s at	1421372 at	up	1.138	up	5.032	up	2.276	up	3.990	up	2.867	3817	KLK2	kallikrein-related peptidase 2	Hs.515560
209854 s at	1449313 at	up	1.138	up	6.763	up	4.282	up	4.136	up	4.316	3817	KLK2	kallikrein-related peptidase 2	Hs.515560
209854 s at	1420701 at	up	1.138	up	5.835	up	2.996	up	6.546	up	4.618	3817	KLK2	kallikrein-related peptidase 2	Hs.515560
209854 s at	1425182 x at	up	1.138	up	4.080	up	2.215	up	8.228	up	4.712	3817	KLK2	kallikrein-related peptidase 2	Hs.515560
209854 s at	1419010 x at	up	1.138	up	7.025	up	5.158	up	4.634	up	5.235	3817	KLK2	kallikrein-related peptidase 2	Hs.515560
209854 s at	1449463 at	up	1.138	up	7.369	up	1.150			up	14.686	3817	KLK2	kallikrein-related peptidase 2	Hs.515560
209854 s at	1420490 at	up	1.138	up	6.190	up	1.421	up	2.764			3817	KLK2	kallikrein-related peptidase 2	Hs.515560
209854 s at	1425144 at	up	1.138	up	2.685			up	3.781			3817	KLK2	kallikrein-related peptidase 2	Hs.515560
209854 s at	1419090 x at	up	1.138	up	2.498			up	3.889			3817	KLK2	kallikrein-related peptidase 2	Hs.515560
209854 s at	1451607 at	up	1.138	up	3.836	up	1.710	up	4.226			3817	KLK2	kallikrein-related peptidase 2	Hs.515560
209854 s at	1421587 at	up	1.138	up	2.601	up	1.392					3817	KLK2	kallikrein-related peptidase 2	Hs.515560
222242 s at	1429230 at	up	1.417	up	10.611	up	2.733	up	7.051	up	7.540	25818	KLK5	kallikrein-related peptidase 5	Hs.50915
239381 at	1423542 at	up	1.603	up	3.450	up	2.270	up	2.159	up	4.113	5650	KLK7	kallikrein-related peptidase 7	Hs.151254
206316 s at	1435575 at	up	1.399	up	2.598	up	2.159	up	1.985	up	2.245	9735	KNTC1	kinetochore associated 1	Hs.300559
207935 s at	1437344 x at	up	1.314					up	1.235			3860	KRT13	keratin 13	Hs.654550
207935 s at	1422454 at	up	1.314	up	4.139	up	2.474					3860	KRT13	keratin 13	Hs.654550
207935 s at	1441863 x at	up	1.314	up	1.392							3860	KRT13	keratin 13	Hs.654550
209800 at	1448932 at	up	1.316			up	2.733	up	2.035	up	2.460	3868	KRT16	keratin 16	Hs.655160
215189 at	1427365 at	up	3.234	up	1.958	up	1.425	up	1.981	up	2.231	00134394 /// 38	KRT86 /// LOC	keratin 86 /// hypothetical protein LOC100134	Hs.278658
213767 at	1442581 at	down	1.260					down	1.193			8844	KSR1	kinase suppressor of ras 1	Hs.133534
213767 at	1419823 s at	down	1.260	down	1.568	down	1.463					8844	KSR1	kinase suppressor of ras 1	Hs.133534
219955 at	1457314 at	up	1.296	up	2.002			up	1.550	up	1.398	54596	L1TD1	LINE-1 type transposase domain containing 1	Hs.685462
229393 at	1443030 at	up	1.369	up	1.265			up	1.441	up	1.266	84456	L3MBTL3	l(3)mbt-like 3 (Drosophila)	Hs.658051
229393 at	1454882 at	up	1.369			up	1.395	up	1.571	up	1.537	84456	L3MBTL3	l(3)mbt-like 3 (Drosophila)	Hs.658051
229393 at	1434246 at	up	1.369	up	1.245	up	1.461	up	1.318			84456	L3MBTL3	l(3)mbt-like 3 (Drosophila)	Hs.658051
217933 s at	1450860 at	up	1.174	up	1.993	up	1.543	up	1.930	up	1.885	51056	LAP3	leucine aminopeptidase 3	Hs.570791
212531 at	1427747 a at	up	1.290	up	2.887	up	6.830	up	3.269	up	2.254	3934	LCN2	lipocalin 2	Hs.204238
206012 at	1436227 at	up	15.628	up	21.708	up	15.376	up	14.801	up	18.277	7044	LEFTY2	left-right determination factor 2	Hs.520187
235809 at	1435181 at	up	1.194			up	1.268	up	1.251	up	1.252	132660	LIN54	lin-54 homolog (C. elegans)	Hs.96952
213909 at	1436055 at	up	8.574	up	2.235	up	2.796	up	2.857	up	2.245	131578	LRRC15	leucine rich repeat containing 15	Hs.288467
228885 at	1453152 at	up	2.079	up	6.803	up	5.347	up	5.485	up	8.050	256691	MAMDC2	MAM domain contair	
226726 at	1425029 a at	up	1.185	up	1.594			up	1.304	up	2.332	129642	MBOAT2	membrane bound O-acyltransferase domain c	Hs.467634
213288 at	1425029 a at	up	1.221	up	1.594			up	1.304	up	2.332	129642	MBOAT2	membrane bound O-acyltransferase domain c	Hs.593538
201930 at	1438852 x at	up	1.131					up	1.544	up	1.868	4175	MCM6	minichromosome maintenance complex comp	Hs.444118
201930 at	1447756 x at	up	1.131	up	1.406			up	1.573			4175	MCM6	minichromosome maintenance complex comp	Hs.444118
212209 at	1453906 at	down	1.143	down	1.916	down	1.288	down	1.621	down	1.554	23389	MED13L	mediator complex subunit 13-like	Hs.603766
212209 at	1434603 at	down	1.143	down	2.061			down	1.815	down	1.765	23389	MED13L	mediator complex subunit 13-like	Hs.603766
212209 at	1434602 at	down	1.143					down	1.975	down	2.049	23389	MED13L	mediator complex subunit 13-like	Hs.603766
223723 at	1419755 at	up	1.480	up	2.399	up	2.135	up	2.149			4241	MFI2	antigen p97 (melanoma associated) identified	Hs.184727
231727 s at	1451523 a at	down	1.131	down	1.409			down	1.211	down	1.347	57409	MIF4GD	MIF4G domain containing	Hs.325631
212020 s at	1426817 at	up	1.373	up	1.752			up	1.939	up	2.366	4288	MKI67	antigen identified by monoclonal antibody Ki-6	Hs.80976
212022 s at	1426817 at	up	1.643	up	1.752			up	1.939	up	2.366	4288	MKI67	antigen identified by monoclonal antibody Ki-6	Hs.80976
204423 at	1416734 at	up	1.204	up	1.603	up	1.320					4289	MKLN1	muskelin 1, intracellular mediator containing k	Hs.44693
209845 at	1418435 at	up	1.246	up	1.189			up	1.292	up	1.272	23608	MKRN1	makorin ring finger protein 1	Hs.490347
238025 at	1429570 at	up	1.288	up	1.610			up	1.531	up	1.589	197259	MLKL	mixed lineage kinase domain-like	Hs.119878
203936 s at	1416298 at	up	1.334	up	2.477	up	1.845	up	1.692	up	2.245	4318	MMP9	matrix metallopeptidase 9 (gelatinase B, 92kD	Hs.297413
203936 s at	1448291 at	up	1.334	up	1.823	up	1.889	up	1.579			4318	MMP9	matrix metallopeptidase 9 (gelatinase B, 92kD	Hs.297413
205330 at	1454867 at	down	1.460	down	1.642	down	1.524			down	1.742	4330	MN1	meningioma (disrupted in balanced translocat	Hs.268515
206764 x at	1440880 at	up	1.206	up	1.660	up	1.440	up	1.415	up	1.426	65258	MPPE1	metallophosphoesterase 1	Hs.712666
213727 x at	1440880 at	up	1.298	up	1.660	up	1.440	up	1.415	up	1.426	65258	MPPE1	metallophosphoesterase 1	Hs.712666
209858 x at	1440880 at	up	1.331	up	1.660	up	1.440	up	1.415	up	1.426	65258	MPPE1	metallophosphoesterase 1	Hs.712666

201875 s at	1428168 at	up	1.114					up	1.253	up	1.354	9019	MPZL1	myelin protein zero-like 1	Hs.493919
210594 x at	1428168 at	up	1.441					up	1.253	up	1.354	9019	MPZL1	myelin protein zero-like 1	Hs.493919
210087 s at	1428168 at	up	1.455					up	1.253	up	1.354	9019	MPZL1	myelin protein zero-like 1	Hs.493919
201875 s at	1428167 a at	up	1.114	up	1.562			up	1.416			9019	MPZL1	myelin protein zero-like 1	Hs.493919
210594 x at	1428167 a at	up	1.441	up	1.562			up	1.416			9019	MPZL1	myelin protein zero-like 1	Hs.493919
210087 s at	1428167 a at	up	1.455	up	1.562			up	1.416			9019	MPZL1	myelin protein zero-like 1	Hs.493919
1554841 at	1429601 x at	up	1.279							up	1.204	441024	MTHFD2L	methylenetetrahydrofolate dehydrogenase (NA	Hs.479954
220346 at	1429601 x at	up	1.432							up	1.204	441024	MTHFD2L	methylenetetrahydrofolate dehydrogenase (NA	Hs.479954
1554841 at	1429599 a at	up	1.279			up	1.304	up	1.219	up	1.234	441024	MTHFD2L	methylenetetrahydrofolate dehydrogenase (NA	Hs.479954
220346 at	1429599 a at	up	1.432			up	1.304	up	1.219	up	1.234	441024	MTHFD2L	methylenetetrahydrofolate dehydrogenase (NA	Hs.479954
1554841 at	1429600 at	up	1.279			up	1.314					441024	MTHFD2L	methylenetetrahydrofolate dehydrogenase (NA	Hs.479954
220346 at	1429600 at	up	1.432			up	1.314					441024	MTHFD2L	methylenetetrahydrofolate dehydrogenase (NA	Hs.479954
1555397 at	1458550 at	up	1.218	up	1.619	up	1.341	up	1.131			4642	MYO1D	myosin ID	Hs.658000
1555397 at	1435463 s at	up	1.218	up	1.987							4642	MYO1D	myosin ID	Hs.658000
216672 s at	1436483 at	up	1.200	up	4.634	up	2.010	up	3.333	up	3.894	23040	MYT1L	myelin transcription factor 1-like	Hs.434418
216672 s at	1429738 at	up	1.200	up	1.217			up	1.201			23040	MYT1L	myelin transcription factor 1-like	Hs.434418
216672 s at	1421175 at	up	1.200	up	1.568	up	1.348	up	1.303			23040	MYT1L	myelin transcription factor 1-like	Hs.434418
222161 at	1422671 s at	up	1.481	up	1.680	up	1.481	up	1.461			10003	NAALAD2	N-acetylated alpha-linked acidic dipeptidase 2	Hs.503560
212789 at	1434426 at	up	1.223					up	1.434	up	1.356	23310	NCAPD3	non-SMC condensin II complex, subunit D3	Hs.438550
212789 at	1454952 s at	up	1.223	up	1.554	up	1.395					23310	NCAPD3	non-SMC condensin II complex, subunit D3	Hs.438550
204641 at	1417299 at	up	1.216	up	1.831	up	1.958	up	1.703	up	1.877	4751	NEK2	NIMA (never in mitosis gene a)-related kinase	Hs.153704
204641 at	1437580 s at	up	1.216	up	2.314	up	3.239	up	2.291	up	2.778	4751	NEK2	NIMA (never in mitosis gene a)-related kinase	Hs.153704
211466 at	1438244 at	up	1.156					up	1.451	up	1.541	4781	NFIB	nuclear factor I/B	Hs.644095
211466 at	1434102 at	up	1.156	up	1.409			up	1.531	up	1.628	4781	NFIB	nuclear factor I/B	Hs.644095
211466 at	1454834 at	up	1.156					up	1.453			4781	NFIB	nuclear factor I/B	Hs.644095
211466 at	1448288 at	up	1.156			up	1.544					4781	NFIB	nuclear factor I/B	Hs.644095
211466 at	1427680 a at	up	1.156	up	1.834							4781	NFIB	nuclear factor I/B	Hs.644095
205858 at	1454903 at	up	4.952	up	1.121	up	1.698	up	1.391			4804	NGFR	nerve growth factor receptor (TNFR superfam	Hs.415768
244320 at	1453048 at	up	1.420	up	1.265			up	1.260	up	1.221	374354	NHLRC2	NHL repeat containing 2	Hs.594372
244320 at	1429145 at	up	1.420					up	1.179			374354	NHLRC2	NHL repeat containing 2	Hs.594372
1552696 at	1434864 at	up	1.274			up	1.543	up	1.400	up	1.520	123606	NIPA1	non imprinted in Prader-Willi/Angelman syndr	Hs.511797
1552696 at	1452763 at	up	1.274	up	1.311							123606	NIPA1	non imprinted in Prader-Willi/Angelman syndr	Hs.511797
224666 at	1455927 x at	down	1.100	down	1.627			down	1.834	down	1.857	197370	NSMCE1	non-SMC element 1 homolog (S. cerevisiae)	Hs.284295
224666 at	1436121 a at	down	1.100					down	2.073	down	2.127	197370	NSMCE1	non-SMC element 1 homolog (S. cerevisiae)	Hs.284295
212247 at	1454743 at	up	1.205	up	1.669	up	1.403			up	1.609	23165	NUP205	nucleoporin 205kDa	Hs.632299
218039 at	1416309 at	up	1.371	up	1.485			up	1.732	up	2.070	51203	NUSAP1	nucleolar and spindle associated protein 1	Hs.615092
202869 at	1424775 at	up	2.663	up	1.879	up	1.660	up	1.826	up	1.953	4938	OAS1	2',5'-oligoadenylate synthetase 1, 40/46kDa	Hs.524760
204972 at	1425065 at	up	3.305	up	1.389			up	1.782	up	1.908	4939	OAS2	2'-5'-oligoadenylate synthetase 2, 69/71kDa	Hs.414332
228607 at	1425065 at	up	3.784	up	1.389			up	1.782	up	1.908	4939	OAS2	2'-5'-oligoadenylate synthetase 2, 69/71kDa	Hs.414332
213131 at	1455796 x at	up	1.412	up	8.807	up	4.353	up	3.806	up	4.633	10439	OLFM1	olfactomedin 1	Hs.522484
213131 at	1425784 a at	up	1.412	up	5.920	up	3.760	up	3.362	up	4.946	10439	OLFM1	olfactomedin 1	Hs.522484
213131 at	1426562 a at	up	1.412	up	1.445	up	1.528					10439	OLFM1	olfactomedin 1	Hs.522484
205041 s at	1420438 at	up	1.174	up	2.777	up	2.332	up	2.014	up	1.623	5004 /// 5005	ORM1 /// ORM:	orosomucoid 1 /// orosomucoid 2	Hs.522356
205041 s at	1451054 at	up	1.174	up	2.897	up	2.292	up	2.725			5004 /// 5005	ORM1 /// ORM:	orosomucoid 1 /// orosomucoid 2	Hs.522356
209791 at	1418252 at	up	1.719	up	2.118	up	1.294	up	1.484	up	1.718	11240	PADI2	peptidyl arginine deiminase, type II	Hs.33455
1554384 at	1418252 at	up	1.801	up	2.118	up	1.294	up	1.484	up	1.718	11240	PADI2	peptidyl arginine deiminase, type II	Hs.33455
220001 at	1422760 at	up	2.234	up	1.990	up	2.288	up	2.056	up	2.007	23569	PADI4	peptidyl arginine deiminase, type IV	Hs.522969
218543 s at	1426774 at	up	1.264	up	3.180	up	2.214	up	2.492	up	2.732	64761	PARP12	poly (ADP-ribose) polymerase family, member	Hs.12646
224701 at	1451564 at	up	1.708	up	2.733	up	1.515	up	1.936	up	2.484	54625	PARP14	poly (ADP-ribose) polymerase family, member	Hs.518203
224701 at	1432548 at	up	1.708			up	1.294					54625	PARP14	poly (ADP-ribose) polymerase family, member	Hs.518203
206594 at	1450993 at	up	1.201	up	3.011	up	1.767	up	1.629	up	1.741	23178	PASK	PAS domain containing serine/threonine kinas	Hs.397891
213534 s at	1450993 at	up	1.379	up	3.011	up	1.767	up	1.629	up	1.741	23178	PASK	PAS domain containing serine/threonine kinas	Hs.397891
216945 x at	1450993 at	up	1.411	up	3.011	up	1.767	up	1.629	up	1.741	23178	PASK	PAS domain containing serine/threonine kinas	Hs.397891
219148 at	1448627 s at	up	1.505	up	1.434			up	1.459	up	1.821	55872	PBK	PDZ binding kinase	Hs.104741
212753 at	1428423 at	up	1.220	up	1.466	up	1.339			up	1.702	10336	PCGF3	polycomb group ring finger 3	Hs.144309
238792 at	1419047 at	up	1.102	up	1.704					up	1.717	22990	PCNX	pecanex homolog (Drosophila)	Hs.446559
213173 at	1419047 at	up	1.233	up	1.704					up	1.717	22990	PCNX	pecanex homolog (Drosophila)	Hs.446559
238792 at	1460580 at	up	1.102			up	1.215					22990	PCNX	pecanex homolog (Drosophila)	Hs.446559
213173 at	1460580 at	up	1.233			up	1.215					22990	PCNX	pecanex homolog (Drosophila)	Hs.446559
1552931 a at	1418407 at	up	1.157	up	1.049					up	1.344	5151	PDE8A	phosphodiesterase 8A	Hs.9333
1552931 a at	1418406 at	up	1.157	up	1.725			up	1.583	up	1.621	5151	PDE8A	phosphodiesterase 8A	Hs.9333
206803 at	1416266 at	up	1.143	up	1.316			up	1.434	up	1.550	5173	PDYN	prodynorphin	Hs.22584
221142 s at	1439167 at	up	1.098					up	1.256	up	1.283	55825	PECR	peroxisomal trans-2-enoyl-CoA reductase	Hs.281680
221142 s at	1448910 at	up	1.098	up	1.242	up	1.402	up	1.440	up	1.417	55825	PECR	peroxisomal trans-2-enoyl-CoA reductase	Hs.281680
1558163 at	1422472 at	down	1.212					down	1.323	down	1.302	5194	PEX13	peroxisomal biogenesis factor 13	Hs.161377
1558163 at	1422471 at	down	1.212	down	1.220							5194	PEX13	peroxisomal biogenesis factor 13	Hs.161377
201968 s at	1437240 at	down	1.189	down	2.197	down	2.101	down	2.487	down	2.084	5236	PGM1	phosphoglucomutase 1	Hs.1869
201968 s at	1451149 at	down	1.189	down	2.163	down	1.722	down	2.423			5236	PGM1	phosphoglucomutase 1	Hs.1869
227947 at	1452404 at	up	1.298	up	1.301			up	1.182	up	1.254	9749	PHACTR2	phosphatase and actin regulator 2	Hs.102471
225842 at	1418835 at	up	1.094			up	1.697	up	1.665	up	1.814	22822	PHLDA1	pleckstrin homology-like domain, family A, me	Hs.602085
209780 at	1437637 at	up	1.234							up	1.416	57157	PHTF2	putative homeodomain transcription factor 2	Hs.203965
209780 at	1444559 at	up	1.234	up	1.541			up	1.382	up	1.557	57157	PHTF2	putative homeodomain transcription factor 2	Hs.203965
209780 at	1437067 at	up	1.234	up	1.584							57157	PHTF2	putative homeodomain transcription factor 2	Hs.203965
212829 at	1442310 at	down	1.232					down	1.097	down	1.088	5305	PIP4K2A	phosphatidylinositol-5-phosphate 4-kinase, typ	Hs.57079
212829 at	1419279 at	down	1.232	down	1.739			down	1.319	down	1.265	5305	PIP4K2A	phosphatidylinositol-5-phosphate 4-kinase, typ	Hs.57079
212829 at	1419280 at	down	1.232	down	1.526							5305	PIP4K2A	phosphatidylinositol-5-phosphate 4-kinase, typ	Hs.57079
212829 at	1449404 at	down	1.232	down	1.787							5305	PIP4K2A	phosphatidylinositol-5-phosphate 4-kinase, typ	Hs.57079
207558 s at	1424797 a at	up	1.325	up	2.153			up	1.468	up	1.629	5308	PITX2	paired-like homeodomain 2	Hs.643588
213222 at	1435043 at	up	1.384	up	4.471	up	4.843	up	2.260	up	3.097	23236	PLCB1	phospholipase C, beta 1 (phosphoinositide-sp	Hs.431173
204887 s at	1426580 at	up	1.414							up	1.233	10733	PLK4	polo-like kinase 4 (Drosophila)	Hs.172052
204886 at	1426580 at	up	1.473							up	1.233	10733	PLK4	polo-like kinase 4 (Drosophila)	Hs.172052
204887 s at	1452115 a at	up	1.414			up	1.826	up	1.437	up	1.641	10733	PLK4	polo-like kinase 4 (Drosophila)	Hs.172052
204886 at	1452115 a at	up	1.473			up	1.826	up	1.437	up	1.641	10733	PLK4	polo-like kinase 4 (Drosophila)	Hs.172052
202620 s at	1416687 at	up	1.656	up	1.505			up	1.496	up	1.804	5352	PLOD2	procollagen-lysine, 2-oxoglutarate 5-dioxygena	Hs.477866
202619 s at	1416687 at	up	2.058	up	1.505			up	1.496	up	1.804	5352	PLOD2	procollagen-lysine, 2-oxoglutarate 5-dioxygena	Hs.477866
223177 s at	1418746 at	up	1.224			up	1.481	up	1.324	up	1.336	25953	PNKD	paroxysmal nonkinesigenic dyskinesia	Hs.98475
228383 at	1451361 a at	up	1.055	up	1.655			up	1.995	up	1.824	375775	PNPLA7	patatin-like phospholipase domain containing	Hs.294147
219756 s at	1427492 at	up	3.825	up	2.623			up	1.433	up	1.582	79983	POF1B	premature ovarian failure, 1B	Hs.267038
216026 s at	1448650 a at	up	1.161	up	1.628			up	1.565	up	1.652	5426	POLE	polymerase (DNA directed), epsilon	Hs.524871
218009 s at	1423775 s at	up	1.320	up	1.706	up	1.706	up	1.604	up	1.979	9055	PRC1	protein regulator of cytokinesis 1	Hs.567385
218009 s at	1423774 a at	up	1.320	up	1.676	up	1.578	up	1.765	up	2.107	9055	PRC1	protein regulator of cytokinesis 1	Hs.567385
208680 at	1436691 x at	up	1.210	up	1.324	up	1.146	up	1.228	up	1.301	5052	PRDX1	peroxiredoxin 1	Hs.180909
208680 at	1437014 x at	up	1.210	up	1.441			up	1.272	up	1.360	5052	PRDX1	peroxiredoxin 1	Hs.180909
208680 at	1434731 x at	up	1.210	up	1.476			up	1.259	up	1.394	5052	PRDX1	peroxiredoxin 1	Hs.180909
208680 at	1433866 x at	up	1.210	up	1.557			up	1.323	up	1.452	5052	PRDX1	peroxiredoxin 1	Hs.180909
208680 at	1416000 a at	up	1.210					up	1.419	up	1.537	5052	PRDX1	peroxiredoxin 1	Hs.180909
228224 at	1416321 s at	up	1.358	up	1.293			up	1.491	up	1.475	5549	PRELP	proline/arginine-rich end leucine-rich repeat pr	Hs.632481
213093 at	1437393 at	down	1.266	down	1.926	down	1.856	down	2.127	down	3.142	5578	PRKCA	protein kinase C, alpha	Hs.531704
213093 at	1450945 at	down	1.266	down	1.906	down	2.240					5578	PRKCA	protein kinase C, alpha	Hs.531704
203537 at	1452062 at	up	1.125			up	1.442	up	1.375	up	1.417	5636	PRPSAP2	phosphoribosyl pyrophosphate synthetase-as	Hs.632236
207638 at	1421510 at	down	1.297	down	1.703	down	1.429	down	2.387	down	1.555	5651	PRSS7	protease, serine, 7 (enterokinase)	Hs.149473
203355 s at	1418749 at	down	1.366	down	3.342	down	2.693	down	1.747			23362	PSD3	pleckstrin and Sec7 domain containing 3	Hs.434255
218613 at	1418749 at	down	1.568	down	3.342	down	2.693	down	1.747			23362	PSD3	pleckstrin and Sec7 domain containing 3	Hs.434255
208776 at	1456059 at	up	1.269					up	1.183	up	1.206	5717	PSMD11	proteasome (prosome, macropain) 26S subur	Hs.655396
208776 at	1440253 at	up	1.269			up	1.337	up	1.304	up	1.523	5717	PSMD11	proteasome (prosome, macropain) 26S subur	Hs.655396
208776 at	1429370 a at	up	1.269					up	1.167			5717	PSMD11	proteasome (prosome, macropain) 26S subur	Hs.655396
211711 s at	1454722 at	down	1.125	down	1.286	down	1.257	down	1.404	down	1.417	5728	PTEN	phosphatase and tensin homolog	Hs.500466
211711 s at	1422553 at	down	1.125	down	1.602							5728	PTEN	phosphatase and tensin homolog	Hs.500466
210834 s at	1450344 a at	down	1.265	down	2.010	down	1.476	down	2.141	down	2.177	5733	PTGER3	prostaglandin E receptor 3 (subtype EP3)	Hs.445000
210831 s at	1450344 a at	down	1.392	down	2.010	down	1.476	down	2.141	down	2.177	5733	PTGER3	prostaglandin E receptor 3 (subtype EP3)	Hs.445000
1555620 a at	1427313 at	down	1.258			down	1.589	down	1.213	down	1.218	5739	PTGIR	prostaglandin I2 (prostacyclin) receptor (IP)	Hs.458324
242775 at	1417777 at	up	1.525	up	6.427	up	2.621	up	3.872	up	4.316	22949	PTGR1	Prostaglandin reductase 1	Hs.584864
228825 at	1417777 at	up	1.548	up	6.427	up	2.621	up	3.872	up	4.316	22949	PTGR1	prostaglandin reductase 1	Hs.584864
230774 at	1443244 at	down	1.212	down	1.128					down	1.154	145482	PTGR2	prostaglandin reductase 2	Hs.632344
230774 at	1429474 at	down	1.212			down	1.439					145482	PTGR2	prostaglandin reductase 2	Hs.632344
218732 at	1455894 at	up	1.207			up	1.236	up	1.200	up	1.278	51651	PTRH2	peptidyl-tRNA hydrolase 2	Hs.12677
212012 at	1428259 at	up	1.186	up	1.745			up	1.509	up	1.533	7837	PXDN	peroxidasin homolog (Drosophila)	Hs.332197
212013 at	1428259 at	up	1.266	up	1.745			up	1.509	up	1.533	7837	PXDN	peroxidasin homolog (Drosophila)	Hs.332197
219705 at	1427152 at	up	1.258	up	2.159	up	1.578	up	1.623	up	1.761	79832	QSER1	glutamine and serine rich 1	Hs.369368
244563 at	1427152 at	up	1.261	up	2.159	up	1.578	up	1.623	up	1.761	79832	QSER1	glutamine and serine rich 1	Hs.369368
219705 at	1427151 at	up	1.258	up	2.177			up	1.742	up	1.970	79832	QSER1	glutamine and serine rich 1	Hs.369368
244563 at	1427151 at	up	1.261	up	2.177			up	1.742	up	1.970	79832	QSER1	glutamine and serine rich 1	Hs.369368
219705 at	1452331 s at	up	1.258	up	1.367	up	1.379	up	1.314			79832	QSER1	glutamine and serine rich 1	Hs.369368
244563 at	1452331 s at	up	1.261	up	1.367	up	1.379	up	1.314			79832	QSER1	glutamine and serine rich 1	Hs.369368
228613 at	1438022 at	up	1.367			up	1.330	up	1.477	up	1.492	9727	RAB11FIP3	RAB11 family interacting protein 3 (class II)	Hs.531642
223463 at	1452113 a at	up	1.218	up	1.262	up	1.319	up	1.295	up	1.290	51715	RAB23	RAB23, member RAS oncogene family	Hs.555016
223471 at	1434306 at	up	1.141	up	1.919	up	1.561	up	1.263	up	1.406	117177	RAB3IP	RAB3A interacting protein (rabin3)	Hs.258209
223471 at	1436103 at	up	1.141	up	1.587	up	1.450					117177	RAB3IP	RAB3A interacting protein (rabin3)	Hs.258209
213603 s at	1417620 at	down	1.019					down	1.699	down	1.673	5880	RAC2	ras-related C3 botulinum toxin substrate 2 (rh	Hs.517601
213603 s at	1440208 at	down	1.019			down	1.435	down	1.424			5880	RAC2	ras-related C3 botulinum toxin substrate 2 (rh	Hs.517601
242689 at	1434669 at	down	1.117	down	1.731	down	1.813	down	1.447	down	1.452	9649	RALGPS1	Ral GEF with PH domain and SH3 binding m	Hs.648175
204199 at	1434669 at	down	1.179	down	1.731	down	1.813	down	1.447	down	1.452	9649	RALGPS1	Ral GEF with PH domain and SH3 binding m	Hs.648175
206367 at	1448975 s at	up	1.480	up	3.950	up	2.906	up	8.905	up	1.743	5972	REN	renin	Hs.3210
227425 at	1435389 at	down	1.321			down	1.594	down	1.482	down	1.901	9185	REPS2	RALBP1 associated Eps domain containing 2	Hs.186810
202388 at	1447830 s at	up	1.637	up	1.942			up	1.611	up	1.577	5997	RGS2	regulator of G-protein signaling 2, 24kDa	Hs.78944
202388 at	1419248 at	up	1.637	up	1.759			up	1.663	up	1.658	5997	RGS2	regulator of G-protein signaling 2, 24kDa	Hs.78944
212651 at	1429206 at	down	1.540	down	1.855	down	1.833	down	2.064	down	1.942	9886	RHOBTB1	Rho-related BTB domain containing 1	Hs.148670
212651 at	1429656 at	down	1.540			down	1.785					9886	RHOBTB1	Rho-related BTB domain containing 1	Hs.148670
219446 at	1435096 at	up	1.373	up	1.600			up	1.344	up	1.494	55188	RIC8B	resistance to inhibitors of cholinesterase 8 ho	Hs.131306
225978 at	1429982 at	up	1.255	up	3.151	up	1.993	up	2.311	up	2.272	57494	RIMKLB	ribosomal modification protein rimK-like family	Hs.504670
229344 x at	1429982 at	up	1.281	up	3.151	up	1.993	up	2.311	up	2.272	57494	RIMKLB	ribosomal modification protein rimK-like family	Hs.504670
225999 at	1429982 at	up	1.296	up	3.151	up	1.993	up	2.311	up	2.272	57494	RIMKLB	ribosomal modification protein rimK-like family	Hs.504670

206851 at	1450616 at	up	1.091	up	10.962	up	7.788	up	4.545	up	8.577	6037	RNASE3	ribonuclease, RNase A family, 3 (eosinophil c	Hs.73839
218269 at	1438505 s at	up	1.075	up	1.591	up	1.478	up	1.268	up	1.315	29102	RNASEN	ribonuclease type III, nuclear	Hs.97997
232060 at	1429313 at	down	1.333	down	2.649	down	1.725	down	2.590	down	2.297	4919	ROR1	receptor tyrosine kinase-like orphan receptor 1	Hs.654491
220738 s at	1429760 at	up	1.246			up	1.969	up	1.553	up	1.779	27330	RPS6KA6	ribosomal protein S6 kinase, 90kDa, polypepti	Hs.368153
213797 at	1421008 at	up	7.168	up	2.048	up	1.391	up	1.676	up	1.515	91543	RSAD2	radical S-adenosyl methionine domain contair	Hs.17518
242625 at	1421008 at	up	10.457	up	2.048	up	1.391	up	1.676	up	1.515	91543	RSAD2	radical S-adenosyl methionine domain contair	Hs.17518
213797 at	1421009 at	up	7.168	up	10.487	up	2.876	up	4.973	up	5.789	91543	RSAD2	radical S-adenosyl methionine domain contair	Hs.17518
242625 at	1421009 at	up	10.457	up	10.487	up	2.876	up	4.973	up	5.789	91543	RSAD2	radical S-adenosyl methionine domain contair	Hs.17518
213797 at	1436058 at	up	7.168	up	8.609	up	2.830	up	5.535	up	7.368	91543	RSAD2	radical S-adenosyl methionine domain contair	Hs.17518
242625 at	1436058 at	up	10.457	up	8.609	up	2.830	up	5.535	up	7.368	91543	RSAD2	radical S-adenosyl methionine domain contair	Hs.17518
219684 at	1418580 at	up	2.731	up	5.535	up	2.619	up	3.693	up	4.210	64108	RTP4	receptor (chemosensory) transporter protein 4	Hs.43388
219684 at	1449143 at	up	2.731	up	1.481	up	1.333	up	1.636			64108	RTP4	receptor (chemosensory) transporter protein 4	Hs.43388
209686 at	1434342 at	up	1.083			up	1.192	up	1.356	up	1.662	6285	S100B	S100 calcium binding protein B	Hs.422181
209686 at	1419383 at	up	1.083	up	1.616			up	1.657	up	1.677	6285	S100B	S100 calcium binding protein B	Hs.422181
228176 at	1438658 a at	up	1.846	up	1.430			up	1.755	up	1.842	1903	S1PR3	sphingosine-1-phosphate receptor 3	
208607 s at	1450826 a at	up	3.263	up	5.011	up	3.875	up	5.870	up	4.068	6288 /// 6289	SAA1 /// SAA2	serum amyloid A1 /// serum amyloid A2	Hs.632144
214456 x at	1450826 a at	up	3.816	up	5.011	up	3.875	up	5.870	up	4.068	6288 /// 6289	SAA1 /// SAA2	serum amyloid A1 /// serum amyloid A2	Hs.632144
208607 s at	1450788 at	up	3.263	up	9.602	up	6.218	up	7.545	up	5.563	6288 /// 6289	SAA1 /// SAA2	serum amyloid A1 /// serum amyloid A2	Hs.632144
214456 x at	1450788 at	up	3.816	up	9.602	up	6.218	up	7.545	up	5.563	6288 /// 6289	SAA1 /// SAA2	serum amyloid A1 /// serum amyloid A2	Hs.632144
208607 s at	1419075 s at	up	3.263	up	12.253	up	7.860	up	9.508	up	7.319	6288 /// 6289	SAA1 /// SAA2	serum amyloid A1 /// serum amyloid A2	Hs.632144
214456 x at	1419075 s at	up	3.816	up	12.253	up	7.860	up	9.508	up	7.319	6288 /// 6289	SAA1 /// SAA2	serum amyloid A1 /// serum amyloid A2	Hs.632144
208607 s at	1449326 x at	up	3.263	up	7.927	up	5.390	up	7.126			6288 /// 6289	SAA1 /// SAA2	serum amyloid A1 /// serum amyloid A2	Hs.632144
214456 x at	1449326 x at	up	3.816	up	7.927	up	5.390	up	7.126			6288 /// 6289	SAA1 /// SAA2	serum amyloid A1 /// serum amyloid A2	Hs.632144
217946 s at	1430021 a at	up	1.175							up	1.152	10055	SAE1	SUMO1 activating enzyme subunit 1	Hs.515500
217946 s at	1440185 x at	up	1.175	up	1.294					up	1.386	10055	SAE1	SUMO1 activating enzyme subunit 1	Hs.515500
217946 s at	1416443 a at	up	1.175					up	1.341	up	1.401	10055	SAE1	SUMO1 activating enzyme subunit 1	Hs.515500
231895 at	1453490 at	up	1.408					up	1.219	up	1.298	163786	SASS6	spindle assembly 6 homolog (C. elegans)	Hs.591447
231895 at	1448016 at	up	1.408	up	2.171	up	2.493	up	1.894	up	2.027	163786	SASS6	spindle assembly 6 homolog (C. elegans)	Hs.591447
206950 at	1442809 at	down	1.269	down	1.841	down	2.090	down	2.063			6335	SCN9A	sodium channel, voltage-gated, type IX, alpha	Hs.439145
229199 at	1442809 at	down	1.811	down	1.841	down	2.090	down	2.063			6335	SCN9A	sodium channel, voltage-gated, type IX, alpha	Hs.439145
206950 at	1442810 x at	down	1.269	down	2.195	down	2.013	down	2.118			6335	SCN9A	sodium channel, voltage-gated, type IX, alpha	Hs.439145
229199 at	1442810 x at	down	1.811	down	2.195	down	2.013	down	2.118			6335	SCN9A	sodium channel, voltage-gated, type IX, alpha	Hs.439145
206950 at	1457819 at	down	1.269	down	2.142							6335	SCN9A	sodium channel, voltage-gated, type IX, alpha	Hs.439145
229199 at	1457819 at	down	1.811	down	2.142							6335	SCN9A	sodium channel, voltage-gated, type IX, alpha	Hs.439145
214433 s at	1417580 s at	up	1.803	up	2.930	up	2.370	up	1.606	up	1.935	8991	SELENBP1	selenium binding protein 1	Hs.632460
214433 s at	1450699 at	up	1.803	up	4.744	up	4.479	up	2.161	up	2.170	8991	SELENBP1	selenium binding protein 1	Hs.632460
210124 x at	1439768 x at	up	1.615	up	5.877	up	2.012	up	1.479			10505	SEMA4F	sema domain, immunoglobulin domain (Ig), tra	Hs.25887
228660 x at	1439768 x at	up	1.721	up	5.877	up	2.012	up	1.479			10505	SEMA4F	sema domain, immunoglobulin domain (Ig), tra	Hs.25887
210124 x at	1419328 at	up	1.615	up	1.967							10505	SEMA4F	sema domain, immunoglobulin domain (Ig), tra	Hs.25887
228660 x at	1419328 at	up	1.721	up	1.967							10505	SEMA4F	sema domain, immunoglobulin domain (Ig), tra	Hs.25887
208940 at	1433974 at	up	1.106					up	1.163	up	1.150	22929	SEPHS1	selenophosphate synthetase 1	Hs.124027
208941 s at	1433974 at	up	1.187					up	1.163	up	1.150	22929	SEPHS1	selenophosphate synthetase 1	Hs.124027
208940 at	1454792 s at	up	1.106	up	1.287			up	1.380	up	1.371	22929	SEPHS1	selenophosphate synthetase 1	Hs.124027
208941 s at	1454792 s at	up	1.187	up	1.287			up	1.380	up	1.371	22929	SEPHS1	selenophosphate synthetase 1	Hs.124027
208940 at	1433973 at	up	1.106					up	1.378			22929	SEPHS1	selenophosphate synthetase 1	Hs.124027
208941 s at	1433973 at	up	1.187					up	1.378			22929	SEPHS1	selenophosphate synthetase 1	Hs.124027
41220 at	1417038 at	down	1.128			down	1.452	down	1.682	down	1.611	10801	SEPT 9	septin 9	Hs.440932
206034 at	1458882 at	up	1.236	up	1.674	up	1.648	up	1.625	up	1.426	5271	SERPINB8	serpin peptidase inhibitor, clade B (ovalbumin	Hs.368077
206034 at	1422776 at	up	1.236	up	2.750	up	2.351	up	2.731	up	7.247	5271	SERPINB8	serpin peptidase inhibitor, clade B (ovalbumin	Hs.368077
214387 x at	1418639 at	up	1.081	up	2.840	up	2.059	up	2.193	up	2.261	6440	SFTPC	surfactant protein C	Hs.1074
242963 at	1428663 at	up	1.454	up	1.850	up	1.484	up	1.401			166929	SGMS2	sphingomyelin synthase 2	Hs.595423
242963 at	1429029 at	up	1.454	up	1.494			up	1.536			166929	SGMS2	sphingomyelin synthase 2	Hs.595423
230459 s at	1434153 at	down	1.124			down	1.245	down	1.234	down	1.226	6461	SHB	Src homology 2 domain containing adaptor pr	Hs.521482
203625 x at	1436000 a at	up	1.342	up	1.668					up	2.129	6502	SKP2	S-phase kinase-associated protein 2 (p45)	Hs.23348
210567 s at	1436000 a at	up	1.948	up	1.668					up	2.129	6502	SKP2	S-phase kinase-associated protein 2 (p45)	Hs.23348
203625 x at	1460247 a at	up	1.342	up	1.806	up	1.590			up	2.736	6502	SKP2	S-phase kinase-associated protein 2 (p45)	Hs.23348
210567 s at	1460247 a at	up	1.948	up	1.806	up	1.590			up	2.736	6502	SKP2	S-phase kinase-associated protein 2 (p45)	Hs.23348
203625 x at	1418969 at	up	1.342	up	1.692			up	2.005	up	3.051	6502	SKP2	S-phase kinase-associated protein 2 (p45)	Hs.23348
210567 s at	1418969 at	up	1.948	up	1.692			up	2.005	up	3.051	6502	SKP2	S-phase kinase-associated protein 2 (p45)	Hs.23348
203625 x at	1437033 a at	up	1.342	up	1.456							6502	SKP2	S-phase kinase-associated protein 2 (p45)	Hs.23348
210567 s at	1437033 a at	up	1.948	up	1.456							6502	SKP2	S-phase kinase-associated protein 2 (p45)	Hs.23348
1555626 a at	1425569 a at	down	1.247							down	1.194	6504	SLAMF1	signaling lymphocytic activation molecule fam	Hs.523660
1555626 a at	1425570 at	down	1.247			down	1.498	down	1.804			6504	SLAMF1	signaling lymphocytic activation molecule fam	Hs.523660
203124 s at	1426441 at	up	1.248					up	1.228	up	1.416	4891	SLC11A2	solute carrier family 11 (proton-coupled divale	Hs.505545
204404 at	1448780 at	up	1.357	up	1.971	up	2.360	up	1.865	up	1.804	6558	SLC12A2	solute carrier family 12 (sodium/potassium/ch	Hs.162585
204404 at	1417622 at	up	1.357	up	2.992	up	1.849	up	1.732	up	2.044	6558	SLC12A2	solute carrier family 12 (sodium/potassium/ch	Hs.162585
204404 at	1417623 at	up	1.357	up	1.695					up	2.167	6558	SLC12A2	solute carrier family 12 (sodium/potassium/ch	Hs.162585
213664 at	1425415 a at	up	1.817	up	3.234	up	2.126	up	2.429	up	1.264	6505	SLC1A1	solute carrier family 1 (neuronal/epithelial high	Hs.444915
213664 at	1448299 at	up	1.817	up	1.913	up	1.649	up	1.830	up	1.810	6505	SLC1A1	solute carrier family 1 (neuronal/epithelial high	Hs.444915
223304 at	1453915 a at	up	1.283	up	1.470	up	1.257	up	1.450	up	1.962	84255	SLC37A3	solute carrier family 37 (glycerol-3-phosphate	Hs.446021
223304 at	1424920 at	up	1.283	up	1.366	up	1.328	up	1.179			84255	SLC37A3	solute carrier family 37 (glycerol-3-phosphate	Hs.446021
243709 at	1436456 at	up	1.236	up	1.477			up	1.278	up	1.395	153129	SLC38A9	solute carrier family 38, member 9	Hs.649685
235241 at	1436456 at	up	1.303	up	1.477			up	1.278	up	1.395	153129	SLC38A9	solute carrier family 38, member 9	Hs.649685
209267 s at	1416832 at	up	1.664	up	5.528	up	2.647	up	2.867	up	2.711	64116	SLC39A8	solute carrier family 39 (zinc transporter), mer	Hs.288034
224931 at	1451707 s at	down	1.142	down	1.282	down	1.572	down	1.779	down	1.777	54946	SLC41A3	solute carrier family 41, member 3	Hs.573007
224931 at	1425439 a at	down	1.142					down	1.219			54946	SLC41A3	solute carrier family 41, member 3	Hs.573007
213113 s at	1422788 at	down	1.205					down	1.501	down	2.133	29015	SLC43A3	solute carrier family 43, member 3	Hs.99962
228696 at	1426664 x at	up	1.226	up	3.048	up	1.621	up	1.528			85414	SLC45A3	solute carrier family 45, member 3	Hs.278695
1552278 a at	1426714 at	up	1.276	up	1.264			up	1.237	up	1.433	113235	SLC46A1	solute carrier family 46 (folate transporter), me	Hs.446689
205592 at	1416464 at	up	1.187			up	1.297	up	1.382	up	1.435	6521	SLC4A1	solute carrier family 4, anion exchanger, member 1 (erythrocyt	
205592 at	1434502 x at	up	1.187	up	2.581	up	2.708	up	2.945	up	3.253	6521	SLC4A1	solute carrier family 4, anion exchanger, member 1 (erythrocyt	
215715 at	1460129 at	up	1.694	up	3.256	up	2.334	up	1.786			6530	SLC6A2	solute carrier family 6 (neurotransmitter transp	Hs.78036
234291 s at	1427221 at	up	1.188	up	1.966	up	1.393	up	1.861			54716	SLC6A20	solute carrier family 6 (proline IMINO transpor	Hs.413095
219885 at	1427102 at	up	1.537	up	5.579	up	3.586	up	6.803	up	9.255	55106	SLFN12	schlafen family member 12	Hs.663548
203021 at	1448377 at	up	1.326	up	14.225	up	7.001	up	7.177	up	6.612	6590	SLPI	secretory leukocyte peptidase inhibitor	Hs.517070
214536 at	1420562 at	up	1.204	up	2.240	up	2.627	up	1.532			57152	SLURP1	secreted LY6/PLAUR domain containing 1	Hs.103505
203874 s at	1460292 a at	up	1.117	up	4.613	up	3.384	up	4.919	up	5.613	6594	SMARCA1	SWI/SNF related, matrix associated, actin dep	Hs.152292
203875 at	1460292 a at	up	1.157	up	4.613	up	3.384	up	4.919	up	5.613	6594	SMARCA1	SWI/SNF related, matrix associated, actin dep	Hs.152292
228370 at	1441905 x at	down	1.291					down	1.477	down	1.588	6638	SNRPN	small nuclear ribonucleoprotein polypeptide N	Hs.564847
228370 at	1455890 x at	down	1.291	down	1.411	down	1.301	down	1.255			6638	SNRPN	small nuclear ribonucleoprotein polypeptide N	Hs.564847
228370 at	1435716 x at	down	1.291	down	1.564	down	1.480	down	1.500			6638	SNRPN	small nuclear ribonucleoprotein polypeptide N	Hs.564847
228370 at	1432096 at	down	1.291	down	1.371							6638	SNRPN	small nuclear ribonucleoprotein polypeptide N	Hs.564847
201522 x at	1441905 x at	down	1.066					down	1.477	down	1.588	6638 /// 8926	SNRPN /// SNU	small nuclear ribonucleoprotein polypeptide N	Hs.555970
201522 x at	1455890 x at	down	1.066	down	1.411	down	1.301	down	1.255			6638 /// 8926	SNRPN /// SNU	small nuclear ribonucleoprotein polypeptide N	Hs.555970
201522 x at	1435716 x at	down	1.066	down	1.564	down	1.480	down	1.500			6638 /// 8926	SNRPN /// SNU	small nuclear ribonucleoprotein polypeptide N	Hs.555970
201522 x at	1432096 at	down	1.066	down	1.371							6638 /// 8926	SNRPN /// SNU	small nuclear ribonucleoprotein polypeptide N	Hs.555970
218404 at	1431055 a at	down	1.218					down	1.696			29887	SNX10	sorting nexin 10	Hs.719227
218404 at	1459764 x at	down	1.218	down	1.876	down	1.900					29887	SNX10	sorting nexin 10	Hs.719227
222594 s at	1460677 at	up	1.235	up	1.886	up	1.630	up	1.456	up	1.532	65244	SPATS2	spermatogenesis associated, serine-rich 2	Hs.654826
209875 s at	1449254 at	up	2.342	up	5.818	up	3.734	up	4.394	up	3.909	6696	SPP1	secreted phosphoprotein 1	Hs.313
212071 s at	1444089 at	up	1.109	up	1.913			up	1.422	up	1.543	6711	SPTBN1	spectrin, beta, non-erythrocytic 1	Hs.705692
212071 s at	1452143 at	up	1.109	up	2.522	up	1.377	up	1.541	up	1.760	6711	SPTBN1	spectrin, beta, non-erythrocytic 1	Hs.705692
212071 s at	1419255 at	up	1.109					up	1.174			6711	SPTBN1	spectrin, beta, non-erythrocytic 1	Hs.705692
212071 s at	1441507 at	up	1.109	up	1.150							6711	SPTBN1	spectrin, beta, non-erythrocytic 1	Hs.705692
212071 s at	1419256 at	up	1.109	up	2.575							6711	SPTBN1	spectrin, beta, non-erythrocytic 1	Hs.705692
217995 at	1420641 a at	up	1.169	up	1.320	up	1.871	up	1.781			58472	SQRDL	sulfide quinone reductase-like (yeast)	Hs.511251
210942 s at	1447841 x at	up	1.195							up	1.234	10402	ST3GAL6	ST3 beta-galactoside alpha-2,3-sialyltransfera	Hs.148716
210942 s at	1449079 s at	up	1.195			up	1.677	up	1.619	up	1.564	10402	ST3GAL6	ST3 beta-galactoside alpha-2,3-sialyltransfera	Hs.148716
210942 s at	1449078 at	up	1.195					up	1.446			10402	ST3GAL6	ST3 beta-galactoside alpha-2,3-sialyltransfera	Hs.148716
210942 s at	1454246 at	up	1.195	up	1.312							10402	ST3GAL6	ST3 beta-galactoside alpha-2,3-sialyltransfera	Hs.148716
205743 at	1418957 at	down	2.099	down	2.597	down	2.333	down	1.686			6769	STAC	SH3 and cysteine rich domain	Hs.56045
AFFX-HUMISG	1420915 at	up	1.184					up	1.545	up	1.577	6772	STAT1	signal transducer and activator of transcriptior	Hs.715518
AFFX-HUMISG	1420915 at	up	1.533					up	1.545	up	1.577	6772	STAT1	signal transducer and activator of transcriptior	Hs.715518
200887 s at	1420915 at	up	1.547					up	1.545	up	1.577	6772	STAT1	signal transducer and activator of transcriptior	Hs.715518
209969 s at	1420915 at	up	2.210					up	1.545	up	1.577	6772	STAT1	signal transducer and activator of transcriptior	Hs.715518
AFFX-HUMISG	1450033 a at	up	1.184					up	1.602			6772	STAT1	signal transducer and activator of transcriptior	Hs.715518
AFFX-HUMISG	1450033 a at	up	1.533					up	1.602			6772	STAT1	signal transducer and activator of transcriptior	Hs.715518
200887 s at	1450033 a at	up	1.547					up	1.602			6772	STAT1	signal transducer and activator of transcriptior	Hs.715518
209969 s at	1450033 a at	up	2.210					up	1.602			6772	STAT1	signal transducer and activator of transcriptior	Hs.715518
AFFX-HUMISG	1450034 at	up	1.184	up	1.378			up	1.634			6772	STAT1	signal transducer and activator of transcriptior	Hs.715518
AFFX-HUMISG	1450034 at	up	1.533	up	1.378			up	1.634			6772	STAT1	signal transducer and activator of transcriptior	Hs.715518
200887 s at	1450034 at	up	1.547	up	1.378			up	1.634			6772	STAT1	signal transducer and activator of transcriptior	Hs.715518
209969 s at	1450034 at	up	2.210	up	1.378			up	1.634			6772	STAT1	signal transducer and activator of transcriptior	Hs.715518
1555086 at	1422103 a at	down	1.131	down	1.686	down	1.737	down	1.616			6777	STAT5B	signal transducer and activator of transcriptior	Hs.719121
1555086 at	1422102 a at	down	1.131			down	1.343					6777	STAT5B	signal transducer and activator of transcriptior	Hs.719121
205339 at	1427707 a at	up	1.278	up	2.437	up	2.658	up	1.850	up	1.953	6491	STIL	SCL/TAL1 interrupting locus	Hs.525198
225250 at	1434219 at	up	1.160	up	1.379	up	1.365	up	1.183	up	1.205	57620	STIM2	stromal interaction molecule 2	Hs.135763
200783 s at	1448113 at	up	1.218	up	2.118			up	1.408	up	1.707	3925	STMN1	stathmin 1	Hs.209983
1553794 at	1447847 x at	up	1.060	up	2.454	up	3.209	up	1.720	up	1.740	161003	STOML3	stomatin (EPB72)-like 3	Hs.327794
1553794 at	1441036 at	up	1.060	up	2.308	up	3.454	up	2.290			161003	STOML3	stomatin (EPB72)-like 3	Hs.327794
215299 x at	1427345 a at	up	1.948	up	2.761	up	1.802	up	2.433	up	1.851	6817	SULT1A1	sulfotransferase family, cytosolic, 1A, phenol-	Hs.567342
203615 x at	1427345 a at	up	1.981	up	2.761	up	1.802	up	2.433	up	1.851	6817	SULT1A1	sulfotransferase family, cytosolic, 1A, phenol-	Hs.567342
227480 at	1426511 at	up	1.946	up	2.100	up	1.517	up	1.267	up	2.227	56241	SUSD2	sushi domain containing 2	Hs.131819
1562372 at	1453715 at	up	1.179	up	2.362	up	1.431	up	1.794	up	2.612	22987	SV2C	synaptic vesicle glycoprotein 2C	Hs.663229
206546 at	1444122 at	up	1.211	up	2.085	up	2.016	up	2.864	up	2.895	10388	SYCP2	synaptonemal complex protein 2	Hs.202676
240197 at	1458105 at	down	1.082	down	1.534	down	1.383	down	1.326	down	1.350	6854	SYN2	synapsin II	Hs.445503
240197 at	1449030 at	down	1.082	down	2.857	down	2.029	down	2.391	down	2.087	6854	SYN2	synapsin II	Hs.445503
240197 at	1460230 at	down	1.082	down	3.133	down	3.120	down	2.954	down	2.785	6854	SYN2	synapsin II	Hs.445503
240197 at	1428460 at	down	1.082	down	4.262	down	3.531	down	3.665	down	3.390	6854	SYN2	synapsin II	Hs.445503
240197 at	1440762 at	down	1.082			down	2.821			down	3.488	6854	SYN2	synapsin II	Hs.445503
240197 at	1435511 at	down	1.082	down	4.080	down	3.304	down	3.828	down	3.623	6854	SYN2	synapsin II	Hs.445503
203999 at	1421990 at	down	1.106	down	1.456	down	1.820	down	1.777	down	1.704	6857	SYT1	synaptotagmin I	Hs.310545

203999 at	1433884 at	down	1.106			down	1.453					6857	SYT1	synaptotagmin I	Hs.310545
203999 at	1438282 at	down	1.106			down	1.499					6857	SYT1	synaptotagmin I	Hs.310545
226167 at	1441927 at	up	1.228					up	1.456	up	1.519	9066	SYT7	synaptotagmin VII	Hs.502730
226167 at	1460081 at	up	1.228			up	1.628	up	1.735	up	2.161	9066	SYT7	synaptotagmin VII	Hs.502730
226167 at	1439633 at	up	1.228	up	1.457	up	1.350	up	1.405			9066	SYT7	synaptotagmin VII	Hs.502730
227703 s at	1417336 a at	up	1.393	up	5.104	up	2.835	up	4.066	up	4.768	94121	SYTL4	synaptotagmin-like 4	Hs.592224
219992 at	1419411 at	up	3.864	up	29.637	up	14.971	up	18.700	up	23.755	6866	TAC3	tachykinin 3	Hs.9730
205943 at	1449337 at	up	1.248	up	3.057			up	7.212	up	4.266	6999	TDO2	tryptophan 2,3-dioxygenase	Hs.183671
205943 at	1419093 at	up	1.248					up	12.029			6999	TDO2	tryptophan 2,3-dioxygenase	Hs.183671
213361 at	1459216 at	up	1.402			up	1.666	up	1.423	up	1.461	23424	TDRD7	tudor domain containing 7	Hs.193842
213361 at	1426716 at	up	1.402							up	1.498	23424	TDRD7	tudor domain containing 7	Hs.193842
201506 at	1415871 at	up	1.945	up	2.016	up	1.789			up	2.093	7045	TGFBI	transforming growth factor, beta-induced, 68k	Hs.369397
201506 at	1448123 s at	up	1.945	up	3.753	up	2.349	up	2.301			7045	TGFBI	transforming growth factor, beta-induced, 68k	Hs.369397
201506 at	1437463 x at	up	1.945	up	3.826	up	2.258					7045	TGFBI	transforming growth factor, beta-induced, 68k	Hs.369397
201506 at	1456250 x at	up	1.945	up	4.213	up	2.271					7045	TGFBI	transforming growth factor, beta-induced, 68k	Hs.369397
229476 s at	1422973 a at	up	1.062					up	1.439	up	1.412	7069	THRSP	thyroid hormone responsive (SPOT14 homolo	Hs.591969
229476 s at	1424737 at	up	1.062			up	1.704	up	2.087			7069	THRSP	thyroid hormone responsive (SPOT14 homolo	Hs.591969
212665 at	1452160 at	up	1.196					up	1.505	up	1.605	25976	TIPARP	TCDD-inducible poly(ADP-ribose) polymerase	Hs.12813
212665 at	1426721 s at	up	1.196	up	1.640			up	1.509	up	1.771	25976	TIPARP	TCDD-inducible poly(ADP-ribose) polymerase	Hs.12813
215033 at	1450958 at	up	1.252	up	9.840	up	13.738	up	8.911	up	8.157	4071	TM4SF1	transmembrane 4 L six family member 1	Hs.715499
209387 s at	1450958 at	up	1.277	up	9.840	up	13.738	up	8.911	up	8.157	4071	TM4SF1	transmembrane 4 L six family member 1	Hs.715499
209386 at	1450958 at	up	1.357	up	9.840	up	13.738	up	8.911	up	8.157	4071	TM4SF1	transmembrane 4 L six family member 1	Hs.715499
215034 s at	1450958 at	up	1.445	up	9.840	up	13.738	up	8.911	up	8.157	4071	TM4SF1	transmembrane 4 L six family member 1	Hs.715499
226489 at	1451794 at	down	1.600	down	1.592	down	1.382	down	1.275	down	1.327	57458	TMCC3	transmembrane and coiled-coil domain family	Hs.370410
235146 at	1451794 at	down	1.813	down	1.592	down	1.382	down	1.275	down	1.327	57458	TMCC3	transmembrane and coiled-coil domain family	Hs.370410
226489 at	1434252 at	down	1.600	down	1.506	down	1.619	down	1.599	down	1.599	57458	TMCC3	transmembrane and coiled-coil domain family	Hs.370410
235146 at	1434252 at	down	1.813	down	1.506	down	1.619	down	1.599	down	1.599	57458	TMCC3	transmembrane and coiled-coil domain family	Hs.370410
226489 at	1435554 at	down	1.600	down	1.638	down	1.533					57458	TMCC3	transmembrane and coiled-coil domain family	Hs.370410
235146 at	1435554 at	down	1.813	down	1.638	down	1.533					57458	TMCC3	transmembrane and coiled-coil domain family	Hs.370410
226489 at	1434253 s at	down	1.600			down	1.659					57458	TMCC3	transmembrane and coiled-coil domain family	Hs.370410
235146 at	1434253 s at	down	1.813			down	1.659					57458	TMCC3	transmembrane and coiled-coil domain family	Hs.370410
226489 at	1454889 x at	down	1.600			down	1.702					57458	TMCC3	transmembrane and coiled-coil domain family	Hs.370410
235146 at	1454889 x at	down	1.813			down	1.702					57458	TMCC3	transmembrane and coiled-coil domain family	Hs.370410
205122 at	1426649 at	up	1.121	up	1.365			up	1.458	up	1.618	8577	TMEFF1	transmembrane protein with EGF-like and two	Hs.598100
205122 at	1441890 x at	up	1.121	up	1.569			up	1.511	up	1.722	8577	TMEFF1	transmembrane protein with EGF-like and two	Hs.598100
224496 s at	1429058 at	up	1.307	up	2.444	up	3.433	up	1.938	up	1.944	84314	TMEM107	transmembrane protein 107	Hs.513933
224495 at	1429058 at	up	1.380	up	2.444	up	3.433	up	1.938	up	1.944	84314	TMEM107	transmembrane protein 107	Hs.513933
224496 s at	1429059 s at	up	1.307	up	2.577	up	3.716	up	1.693			84314	TMEM107	transmembrane protein 107	Hs.513933
224495 at	1429059 s at	up	1.380	up	2.577	up	3.716	up	1.693			84314	TMEM107	transmembrane protein 107	Hs.513933
218834 s at	1416845 at	up	1.317			up	1.229	up	1.184	up	1.269	54972	TMEM132A	transmembrane protein 132A	Hs.118552
235245 at	1444142 at	up	1.718	up	2.834	up	2.199	up	2.353	up	2.106	162461	TMEM92	transmembrane protein 92	Hs.224630
201207 at	1417866 at	up	1.201					up	1.169	up	1.218	7126	TNFAIP1	tumor necrosis factor, alpha-induced protein 1	Hs.76090
201208 s at	1417866 at	up	1.434					up	1.169	up	1.218	7126	TNFAIP1	tumor necrosis factor, alpha-induced protein 1	Hs.76090
201207 at	1417865 at	up	1.201	up	1.542					up	1.454	7126	TNFAIP1	tumor necrosis factor, alpha-induced protein 1	Hs.76090
201208 s at	1417865 at	up	1.434	up	1.542					up	1.454	7126	TNFAIP1	tumor necrosis factor, alpha-induced protein 1	Hs.76090
209904 at	1418370 at	up	1.846	up	1.952	up	1.623	up	1.755			7134	TNNC1	troponin C type 1 (slow)	Hs.118845
203050 at	1435609 at	up	1.208					up	1.085	up	1.214	7158	TP53BP1	tumor protein p53 binding protein 1	Hs.440968
203050 at	1426957 at	up	1.208			up	1.252					7158	TP53BP1	tumor protein p53 binding protein 1	Hs.440968
203050 at	1426956 a at	up	1.208			up	1.424					7158	TP53BP1	tumor protein p53 binding protein 1	Hs.440968
1554001 at	1455012 s at	up	1.729	up	1.519	up	1.510			up	1.459	4591	TRIM37	tripartite motif-containing 37	Hs.579079
1554001 at	1434554 at	up	1.729			up	1.390					4591	TRIM37	tripartite motif-containing 37	Hs.579079
204033 at	1429295 s at	up	1.301	up	2.059			up	1.455	up	1.904	9319	TRIP13	thyroid hormone receptor interactor 13	Hs.436187
205028 at	1426016 a at	up	1.390			up	1.684	up	1.738	up	2.080	7216	TRO	trophinin	Hs.633653
211701 s at	1426016 a at	up	1.447			up	1.684	up	1.738	up	2.080	7216	TRO	trophinin	Hs.633653
205028 at	1421201 a at	up	1.390	up	1.263							7216	TRO	trophinin	Hs.633653
211701 s at	1421201 a at	up	1.447	up	1.263							7216	TRO	trophinin	Hs.633653
218693 at	1424653 at	up	1.412	up	2.009	up	1.646	up	1.300	up	1.245	23555	TSPAN15	tetraspanin 15	Hs.499941
1561449 at	1429856 at	down	1.271			down	2.113	down	2.977	down	3.177	90139	TSPAN18	tetraspanin 18	Hs.638940
1561449 at	1442174 at	down	1.271	down	1.621	down	1.440	down	1.245			90139	TSPAN18	tetraspanin 18	Hs.638940
1555307 at	1428522 at	up	1.135	up	1.480			up	1.407	up	1.544	8458	TTF2	transcription termination factor, RNA polymera	Hs.486818
204407 at	1428522 at	up	1.215	up	1.480			up	1.407	up	1.544	8458	TTF2	transcription termination factor, RNA polymera	Hs.486818
208977 x at	1456078 x at	up	1.254							up	1.490	10383	TUBB2C	tubulin, beta 2C	Hs.433615
213726 x at	1456078 x at	up	1.269							up	1.490	10383	TUBB2C	tubulin, beta 2C	Hs.433615
208977 x at	1423642 at	up	1.254			up	1.689	up	1.422	up	1.613	10383	TUBB2C	tubulin, beta 2C	Hs.433615
213726 x at	1423642 at	up	1.269			up	1.689	up	1.422	up	1.613	10383	TUBB2C	tubulin, beta 2C	Hs.433615
208977 x at	1439416 x at	up	1.254	up	1.693	up	1.557					10383	TUBB2C	tubulin, beta 2C	Hs.433615
213726 x at	1439416 x at	up	1.269	up	1.693	up	1.557					10383	TUBB2C	tubulin, beta 2C	Hs.433615
208977 x at	1438622 x at	up	1.254	up	1.533							10383	TUBB2C	tubulin, beta 2C	Hs.433615
213726 x at	1438622 x at	up	1.269	up	1.533							10383	TUBB2C	tubulin, beta 2C	Hs.433615
206733 at	1417276 at	down	1.214	down	2.865	down	1.849	down	2.032	down	2.018	7288	TULP2	tubby like protein 2	Hs.104636
205694 at	1415862 at	up	2.083			up	1.247			up	1.236	7306	TYRP1	tyrosinase-related protein 1	Hs.270279
205694 at	1439409 x at	up	2.083	up	1.732	up	1.438	up	1.291	up	1.627	7306	TYRP1	tyrosinase-related protein 1	Hs.270279
205694 at	1415861 at	up	2.083	up	1.907	up	1.841	up	1.678	up	1.682	7306	TYRP1	tyrosinase-related protein 1	Hs.270279
229587 at	1416280 at	up	1.297							up	1.351	10054	UBA2	ubiquitin-like modifier activating enzyme 2	Hs.631580
229587 at	1448283 a at	up	1.297	up	1.223			up	1.153			10054	UBA2	ubiquitin-like modifier activating enzyme 2	Hs.631580
229587 at	1433440 x at	up	1.297	up	1.234							10054	UBA2	ubiquitin-like modifier activating enzyme 2	Hs.631580
203343 at	1416308 at	up	1.261	up	1.272	up	1.433	up	1.346			7358	UGDH	UDP-glucose dehydrogenase	Hs.572518
1553183 at	1439211 at	up	1.360			up	1.178	up	1.258	up	1.240	89766	UMODL1	uromodulin-like 1	Hs.242520
213100 at	1453269 at	up	1.229					up	2.037	up	3.147	219699	UNC5B	Unc-5 homolog B (C. elegans)	Hs.522997
226899 at	1453269 at	up	5.688					up	2.037	up	3.147	219699	UNC5B	unc-5 homolog B (C. elegans)	Hs.522997
213100 at	1435110 at	up	1.229	up	5.314	up	2.180	up	2.188	up	3.455	219699	UNC5B	Unc-5 homolog B (C. elegans)	Hs.522997
226899 at	1435110 at	up	5.688	up	5.314	up	2.180	up	2.188	up	3.455	219699	UNC5B	unc-5 homolog B (C. elegans)	Hs.522997
225925 s at	1419278 at	up	1.146					up	1.226	up	1.237	84196	USP48	ubiquitin specific peptidase 48	Hs.467524
220078 at	1419278 at	up	1.182					up	1.226	up	1.237	84196	USP48	ubiquitin specific peptidase 48	Hs.467524
225925 s at	1424056 at	up	1.146	up	1.231							84196	USP48	ubiquitin specific peptidase 48	Hs.467524
220078 at	1424056 at	up	1.182	up	1.231							84196	USP48	ubiquitin specific peptidase 48	Hs.467524
204761 at	1455580 at	up	1.087	up	1.282	up	1.318	up	1.259	up	1.315	9712	USP6NL	USP6 N-terminal like	Hs.498661
204761 at	1440883 at	up	1.087	up	1.530					up	1.565	9712	USP6NL	USP6 N-terminal like	Hs.498661
220327 at	1430596 s at	up	1.289	up	1.451			up	1.470	up	1.470	389136	VGLL3	vestigial like 3 (Drosophila)	Hs.435013
227399 at	1430596 s at	up	1.291	up	1.451			up	1.470	up	1.470	389136	VGLL3	vestigial like 3 (Drosophila)	Hs.435013
220327 at	1453593 at	up	1.289	up	1.648							389136	VGLL3	vestigial like 3 (Drosophila)	Hs.435013
227399 at	1453593 at	up	1.291	up	1.648							389136	VGLL3	vestigial like 3 (Drosophila)	Hs.435013
205844 at	1418486 at	up	1.412	up	6.510	up	4.162	up	5.916	up	6.187	8876	VNN1	vanin 1	Hs.12114
205844 at	1447845 s at	up	1.412	up	8.072	up	4.907	up	6.628	up	6.835	8876	VNN1	vanin 1	Hs.12114
204165 at	1418545 at	up	1.099	up	1.732	up	1.390	up	1.368	up	1.436	8936	WASF1	WAS protein family, member 1	Hs.75850
216228 s at	1435114 at	up	1.302	up	1.306			up	1.328	up	1.527	11169	WDHD1	WD repeat and HMG-box DNA binding protei	Hs.385998
204727 at	1435114 at	up	1.310	up	1.306			up	1.328	up	1.527	11169	WDHD1	WD repeat and HMG-box DNA binding protei	Hs.385998
204728 s at	1435114 at	up	1.415	up	1.306			up	1.328	up	1.527	11169	WDHD1	WD repeat and HMG-box DNA binding protei	Hs.385998
226890 at	1454790 at	up	1.211	up	1.282	up	1.480	up	1.145	up	1.190	57539	WDR35	WD repeat domain 35	Hs.205427
223308 s at	1448389 at	up	1.287					up	1.189	up	1.146	11091	WDR5	WD repeat domain 5	Hs.397638
223308 s at	1416581 at	up	1.287			up	1.148	up	1.277	up	1.299	11091	WDR5	WD repeat domain 5	Hs.397638
214061 at	1434241 at	up	1.313	up	1.633	up	1.597	up	1.403			93594	WDR67	WD repeat domain 67	Hs.492716
1556429 a at	1434241 at	up	1.508	up	1.633	up	1.597	up	1.403			93594	WDR67	WD repeat domain 67	Hs.492716
202908 at	1448411 at	up	1.287	up	1.617			up	1.299	up	1.357	7466	WFS1	Wolfram syndrome 1 (wolframin)	Hs.518602
213085 s at	1420008 s at	up	1.447	up	2.178	up	1.556	up	1.420	up	1.341	23286	WWC1	WW and C2 domain containing 1	Hs.484047
213085 s at	1427261 at	up	1.447					up	1.170			23286	WWC1	WW and C2 domain containing 1	Hs.484047
213085 s at	1420009 at	up	1.447			up	1.184					23286	WWC1	WW and C2 domain containing 1	Hs.484047
213342 at	1416487 a at	up	1.201	up	1.711	up	1.546	up	1.707	up	1.459	10413	YAP1	Yes-associated protein 1, 65kDa	Hs.503692
213342 at	1448363 at	up	1.201	up	1.993	up	1.580	up	1.966	up	1.740	10413	YAP1	Yes-associated protein 1, 65kDa	Hs.503692
214686 at	1454812 at	up	1.274			up	1.113	up	1.235	up	1.259	10781	ZNF266	zinc finger protein 266	Hs.656185
227195 at	1423835 at	up	1.204	up	2.909	up	1.970	up	1.909	up	2.398	84858	ZNF503	zinc finger protein 503	
222606 at	1416757 at	up	1.450	up	1.729			up	1.724	up	1.921	55055	ZWILCH	Zwilch, kinetochore associated, homolog (Dro	Hs.21331

Entrez Gene (Murine)	Gene Symbol (Murine)	Gene Title (Murine)	UniGene ID (Murine)
268860	Abat	4-aminobutyrate aminotransferase	Mm.259315
268860	Abat	4-aminobutyrate aminotransferase	Mm.259315
268379	Abca13	ATP-binding cassette, sub-family A (ABC1),	Mm.39270
268379	Abca13	ATP-binding cassette, sub-family A (ABC1),	Mm.39270
268379	Abca13	ATP-binding cassette, sub-family A (ABC1),	Mm.39270
268379	Abca13	ATP-binding cassette, sub-family A (ABC1),	Mm.39270
235534	Acpl2	acid phosphatase-like 2	Mm.233031
235534	Acpl2	acid phosphatase-like 2	Mm.233031
0045780 /// 114	Adam19 /// LOC	a disintegrin and metallopeptidase domain 19	Mm.89940
0045780 /// 114	Adam19 /// LOC	a disintegrin and metallopeptidase domain 19	Mm.89940
26876	Adh4	alcohol dehydrogenase 4 (class II), pi polypep	Mm.158750
104923	Adi1	acireductone dioxygenase 1	Mm.291504
104923	Adi1	acireductone dioxygenase 1	Mm.291504
104923	Adi1	acireductone dioxygenase 1	Mm.291504
104923	Adi1	acireductone dioxygenase 1	Mm.291504
104923	Adi1	acireductone dioxygenase 1	Mm.291504
104923	Adi1	acireductone dioxygenase 1	Mm.291504
434756	Akap14	A kinase (PRKA) anchor protein 14	Mm.471848
77337	Akr1c21	aldo-keto reductase family 1, member C1 (dih	Mm.37605
77337	Akr1c21	aldo-keto reductase family 1, member C1 (dih	Mm.27085
77337	Akr1c21	aldo-keto reductase family 1, member C2 (dih	Mm.27085
77337	Akr1c21	aldo-keto reductase family 1, member C2 (dih	Mm.27085
105349	Akr1c18	aldo-keto reductase family 1, member C18	Mm.41337
11668	Aldh1a1	aldehyde dehydrogenase family 1, subfamily A	Mm.435667
230163	Aldob	aldolase B, fructose-bisphosphate	Mm.218862
11687	Alox15	arachidonate 15-lipoxygenase	Mm.4584
59008	Anapc5	anaphase-promoting complex subunit 5	Mm.332667
59008	Anapc5	anaphase-promoting complex subunit 5	Mm.332667
59008	Anapc5	anaphase-promoting complex subunit 5	Mm.332667
59008	Anapc5	anaphase-promoting complex subunit 5	Mm.332667
59008	Anapc5	anaphase-promoting complex subunit 5	Mm.332667
11735	Ank3	ankyrin 3, node of Ranvier (ankyrin G)	Mm.235960
11735	Ank3	ankyrin 3, node of Ranvier (ankyrin G)	Mm.235960
11735	Ank3	ankyrin 3, node of Ranvier (ankyrin G)	Mm.235960
11735	Ank3	ankyrin 3, node of Ranvier (ankyrin G)	Mm.235960
11735	Ank3	ankyrin 3, node of Ranvier (ankyrin G)	Mm.235960
11735	Ank3	ankyrin 3, node of Ranvier (ankyrin G)	Mm.235960
68743	Anln	anillin, actin binding protein	Mm.282751
11761	Aox1	aldehyde oxidase 1	Mm.26787
252903	Ap1s3	adaptor-related protein complex AP-1, sigma	Mm.32409
11815	Apod	apolipoprotein D	Mm.2082
11826	Aqp1	aquaporin 1	Mm.18625
11828	Aqp3	aquaporin 3	Mm.34043
11828	Aqp3	aquaporin 3	Mm.34043
73910	Arhgap18	Rho GTPase activating protein 18	Mm.356496
73910	Arhgap18	Rho GTPase activating protein 18	Mm.356496
73910	Arhgap18	Rho GTPase activating protein 18	Mm.356496
73910	Arhgap18	Rho GTPase activating protein 18	Mm.356496
65973	Asph	aspartate-beta-hydroxylase	Mm.222206
65973	Asph	aspartate-beta-hydroxylase	Mm.222206
65973	Asph	aspartate-beta-hydroxylase	Mm.222206
65973	Asph	aspartate-beta-hydroxylase	Mm.222206
65973	Asph	aspartate-beta-hydroxylase	Mm.222206
65973	Asph	aspartate-beta-hydroxylase	Mm.222206
66514	Asrgl1	asparaginase like 1	Mm.272847
66514	Asrgl1	asparaginase like 1	Mm.272847
66514	Asrgl1	asparaginase like 1	Mm.272847
70472	Atad2	ATPase family, AAA domain containing 2	Mm.221758
70472	Atad2	ATPase family, AAA domain containing 2	Mm.221758
70472	Atad2	ATPase family, AAA domain containing 2	Mm.221758
224088	Atp13a3	ATPase type 13A3	Mm.8924
11938	Atp2a2	ATPase, Ca++ transporting, cardiac muscle,	Mm.227583
11938	Atp2a2	ATPase, Ca++ transporting, cardiac muscle,	Mm.227583
11938	Atp2a2	ATPase, Ca++ transporting, cardiac muscle,	Mm.227583
11938	Atp2a2	ATPase, Ca++ transporting, cardiac muscle,	Mm.227583
11938	Atp2a2	ATPase, Ca++ transporting, cardiac muscle,	Mm.227583
11941	Atp2b2	ATPase, Ca++ transporting, plasma membra	Mm.321755
20878	Aurka	aurora kinase A	Mm.249363
20878	Aurka	aurora kinase A	Mm.249363
27078	B9d1	B9 protein domain 1	Mm.261226
56175	Bace2	beta-site APP-cleaving enzyme 2	Mm.97885
56175	Bace2	beta-site APP-cleaving enzyme 2	Mm.97885
56175	Bace2	beta-site APP-cleaving enzyme 2	Mm.97885
56175	Bace2	beta-site APP-cleaving enzyme 2	Mm.97885
68010	Bambi	BMP and activin membrane-bound inhibitor, h	Mm.284863
14025	Bcl11a	B-cell CLL/lymphoma 11A (zinc finger protein	Mm.53687
14025	Bcl11a	B-cell CLL/lymphoma 11A (zinc finger protein	Mm.53687
4 /// 12045 /// 1	Bcl2a1a /// Bcl2	B-cell leukemia/lymphoma 2 related protein A	Mm.378888
12045 /// 12046	Bcl2a1a /// Bcl2	B-cell leukemia/lymphoma 2 related protein A	Mm.378888
4 /// 12045 /// 1	Bcl2a1a /// Bcl2	B-cell leukemia/lymphoma 2 related protein A	Mm.378888
12122	Bid	BH3 interacting domain death agonist	Mm.235081
12122	Bid	BH3 interacting domain death agonist	Mm.235081
12144	Blm	Bloom syndrome homolog (human)	Mm.12932
171543	Bmf	BCL2 modifying factor	Mm.210125
69550	Bst2	bone marrow stromal cell antigen 2	Mm.260325
227545	5430407P10Ril	RIKEN cDNA 5430407P10 gene	Mm.133542
77744	6720463M24Ri	RIKEN cDNA 6720463M24 gene	Mm.23503
77744	6720463M24Ri	RIKEN cDNA 6720463M24 gene	Mm.23503
75216	4930534B04Ril	RIKEN cDNA 4930534B04 gene	Mm.312204
75216	4930534B04Ril	RIKEN cDNA 4930534B04 gene	Mm.312204
75216	4930534B04Ril	RIKEN cDNA 4930534B04 gene	Mm.312204
75216	4930534B04Ril	RIKEN cDNA 4930534B04 gene	Mm.312204
75216	4930534B04Ril	RIKEN cDNA 4930534B04 gene	Mm.312204
75216	4930534B04Ril	RIKEN cDNA 4930534B04 gene	Mm.312204
51944	D2Ertd750e	DNA segment, Chr 2, ERATO Doi 750, expre	Mm.252695
51944	D2Ertd750e	DNA segment, Chr 2, ERATO Doi 750, expre	Mm.252695
51944	D2Ertd750e	DNA segment, Chr 2, ERATO Doi 750, expre	Mm.252695
51944	D2Ertd750e	DNA segment, Chr 2, ERATO Doi 750, expre	Mm.252695
381306	BC055324	cDNA sequence BC055324	Mm.28152
381306	BC055324	cDNA sequence BC055324	Mm.28152
69399	1700025G04Ril	RIKEN cDNA 1700025G04 gene	Mm.278031
69399	1700025G04Ril	RIKEN cDNA 1700025G04 gene	Mm.278031
69399	1700025G04Ril	RIKEN cDNA 1700025G04 gene	Mm.278031
69399	1700025G04Ril	RIKEN cDNA 1700025G04 gene	Mm.278031
68001	1110004E09Ril	RIKEN cDNA 1110004E09 gene	Mm.45374
68001	1110004E09Ril	RIKEN cDNA 1110004E09 gene	Mm.45374
12268	C4b	complement component 4B (Childo blood gro	Mm.439678
68646	1110020G09Ril	RIKEN cDNA 1110020G09 gene	Mm.244226
68646	1110020G09Ril	RIKEN cDNA 1110020G09 gene	Mm.244226
68646	1110020G09Ril	RIKEN cDNA 1110020G09 gene	Mm.244226
109254	9530008L14Rik	RIKEN cDNA 9530008L14 gene	Mm.25195
544696	D630037F22Ril	RIKEN cDNA D630037F22 gene	Mm.411188
71934	Car13	carbonic anhydrase 13	Mm.158776
71934	Car13	carbonic anhydrase 13	Mm.158776
71934	Car13	carbonic anhydrase 13	Mm.158776
12351	Car4	carbonic anhydrase 4	Mm.1641
12351	Car4	carbonic anhydrase 4	Mm.1641
12351	Car4	carbonic anhydrase 4	Mm.1641
12351	Car4	carbonic anhydrase 4	Mm.1641
63955	Cables1	CDK5 and Abl enzyme substrate 2	Mm.40717
63955	Cables1	CDK5 and Abl enzyme substrate 3	Mm.40717
63955	Cables1	CDK5 and Abl enzyme substrate 2	Mm.40717
63955	Cables1	CDK5 and Abl enzyme substrate 3	Mm.40717
63955	Cables1	CDK5 and Abl enzyme substrate 1 (Cables1)	Mm.40717
63955	Cables1	CDK5 and Abl enzyme substrate 1 (Cables1)	Mm.40717
56808	Cacna2d2	calcium channel, voltage-dependent, alpha 2/	Mm.273084
140723	Cacng5	calcium channel, voltage-dependent, gamma	Mm.87663
54725	Cadm1	cell adhesion molecule 1	Mm.234832
54725	Cadm1	cell adhesion molecule 1	Mm.234832
54725	Cadm1	cell adhesion molecule 1	Mm.234832
54725	Cadm1	cell adhesion molecule 1	Mm.234832
54725	Cadm1	cell adhesion molecule 1	Mm.234832
54725	Cadm1	cell adhesion molecule 1	Mm.234832
54725	Cadm1	cell adhesion molecule 1	Mm.234832
54725	Cadm1	cell adhesion molecule 1	Mm.234832
54725	Cadm1	cell adhesion molecule 1	Mm.234832
12326	Camk4	calcium/calmodulin-dependent protein kinase	Mm.222329
12326	Camk4	calcium/calmodulin-dependent protein kinase	Mm.222329

12326	Camk4	calcium/calmodulin-dependent protein kinase	Mm.222329
12796	Camp	cathelicidin antimicrobial peptide	Mm.3834
12359	Cat	catalase	Mm.4215
12359	Cat	catalase	Mm.4215
12359	Cat	catalase	Mm.4215
12359	Cat	catalase	Mm.4215
59289	Ccbp2	chemokine binding protein 2	Mm.258105
59289	Ccbp2	chemokine binding protein 2	Mm.258105
73254	Ccdc18	coiled-coil domain containing 18	Mm.441231
73254	Ccdc18	coiled-coil domain containing 18	Mm.441231
212514	Ccdc52	coiled-coil domain containing 52	Mm.24035
12428	Ccna2	cyclin A2	Mm.4189
12428	Ccna2	cyclin A2	Mm.4189
12428	Ccna2	cyclin A2	Mm.4189
12428	Ccna2	cyclin A2	Mm.4189
0047134 /// 124	Ccrn4l /// LOC1	CCR4 carbon catabolite repression 4-like (S.	Mm.86541
260409	Cdc42ep3	CDC42 effector protein (Rho GTPase binding	Mm.140601
260409	Cdc42ep3	CDC42 effector protein (Rho GTPase binding	Mm.140601
260409	Cdc42ep3	CDC42 effector protein (Rho GTPase binding	Mm.140601
260409	Cdc42ep3	CDC42 effector protein (Rho GTPase binding	Mm.140601
67849	Cdca5	cell division cycle associated 5	Mm.23526
67849	Cdca5	cell division cycle associated 5	Mm.23526
52276	Cdca8	cell division cycle associated 8	Mm.28038
52276	Cdca8	cell division cycle associated 8	Mm.28038
52276	Cdca8	cell division cycle associated 8	Mm.28038
12562	Cdh5	cadherin 5	Mm.21767
0047490 /// 134	Cdk2ap1 /// LO(	CDK2 (cyclin-dependent kinase 2)-associated	Mm.425284
12577	Cdkn1c	cyclin-dependent kinase inhibitor 1C (P57)	Mm.168789
12577	Cdkn1c	cyclin-dependent kinase inhibitor 1C (P57)	Mm.168789
12577	Cdkn1c	cyclin-dependent kinase inhibitor 1C (P57)	Mm.168789
12577	Cdkn1c	cyclin-dependent kinase inhibitor 1C (P57)	Mm.168789
12577	Cdkn1c	cyclin-dependent kinase inhibitor 1C (P57)	Mm.168789
108000	Cenpf	centromere protein F	Mm.129746
108000	Cenpf	centromere protein F	Mm.129746
99100	Cep152	centrosomal protein 152	Mm.330545
50883	Chek2	CHK2 checkpoint homolog (S. pombe)	Mm.279308
214425	Cilp	cartilage intermediate layer protein, nucleotide	Mm.138455
66197	Cks2	CDC28 protein kinase regulatory subunit 2	Mm.222228
66197	Cks2	CDC28 protein kinase regulatory subunit 2	Mm.222228
229933	Clca5	chloride channel calcium activated 5	Mm.331420
12745	Clgn	calmegin	Mm.358581
12745	Clgn	calmegin	Mm.358581
26889	Cln8	ceroid-lipofuscinosis, neuronal 8	Mm.254027
26889	Cln8	ceroid-lipofuscinosis, neuronal 8	Mm.254027
26889	Cln8	ceroid-lipofuscinosis, neuronal 8	Mm.254027
12764	Cmas	cytidine monophospho-N-acetylneuraminic ac	Mm.3820
21367	Cntn2	contactin 2	Mm.477739
12818	Col14a1	collagen, type XIV, alpha 1	Mm.297859
12818	Col14a1	collagen, type XIV, alpha 1	Mm.297859
12842	Col1a1	collagen, type I, alpha 1	Mm.277735
12842	Col1a1	collagen, type I, alpha 1	Mm.277735
12842	Col1a1	collagen, type I, alpha 1	Mm.277735
12833	Col6a1	collagen, type VI, alpha 1	Mm.2509
12841	Col9a3	collagen, type IX, alpha 3	Mm.141312
12845	Comp	cartilage oligomeric matrix protein	Mm.45071
71883	Coq2	coenzyme Q2 homolog, prenyltransferase (ye	Mm.260661
70574	Cpm	carboxypeptidase M	Mm.339332
70574	Cpm	carboxypeptidase M	Mm.339332
234577	Cpne2	copine II	Mm.291815
227231	Cps1	carbamoyl-phosphate synthetase 1	Mm.343942
227231	Cps1	carbamoyl-phosphate synthetase 1	Mm.343942
72832	Crtac1	Cartilage acidic protein 1 (Crtac1), mRNA	Mm.313558
72832	Crtac1	cartilage acidic protein 1	Mm.313558
72832	Crtac1	cartilage acidic protein 1	Mm.313558
12955	Cryab	crystallin, alpha B	Mm.178
12955	Cryab	crystallin, alpha B	Mm.178
14219	Ctgf	connective tissue growth factor	Mm.393058
107869	Cth	cystathionase (cystathionine gamma-lyase)	Mm.28301
14825	Cxcl1	chemokine (C-X-C motif) ligand 1	Mm.21013
14825	Cxcl1	chemokine (C-X-C motif) ligand 1	Mm.21013
14825	Cxcl1	chemokine (C-X-C motif) ligand 1	Mm.21013
13078	Cyp1b1	cytochrome P450, family 1, subfamily b, polyp	Mm.214016
13078	Cyp1b1	cytochrome P450, family 1, subfamily b, polyp	Mm.214016
13078	Cyp1b1	cytochrome P450, family 1, subfamily b, polyp	Mm.214016
13078	Cyp1b1	cytochrome P450, family 1, subfamily b, polyp	Mm.214016
13078	Cyp1b1	cytochrome P450, family 1, subfamily b, polyp	Mm.214016
13078	Cyp1b1	cytochrome P450, family 1, subfamily b, polyp	Mm.214016
13138	Dag1	dystroglycan 1	Mm.7524
73379	Dcbld2	discoidin, CUB and LCCL domain containing	Mm.373589
73379	Dcbld2	discoidin, CUB and LCCL domain containing	Mm.373589
69219	Ddah1	dimethylarginine dimethylaminohydrolase 1	Mm.234247
69219	Ddah1	dimethylarginine dimethylaminohydrolase 1	Mm.234247
69219	Ddah1	dimethylarginine dimethylaminohydrolase 1	Mm.234247
69219	Ddah1	dimethylarginine dimethylaminohydrolase 1	Mm.234247
230073	Ddx58	DEAD (Asp-Glu-Ala-Asp) box polypeptide 58	Mm.86382
230073	Ddx58	DEAD (Asp-Glu-Ala-Asp) box polypeptide 58	Mm.86382
230073	Ddx58	DEAD (Asp-Glu-Ala-Asp) box polypeptide 58	Mm.86382
230073	Ddx58	DEAD (Asp-Glu-Ala-Asp) box polypeptide 58	Mm.86382
230073	Ddx58	DEAD (Asp-Glu-Ala-Asp) box polypeptide 58	Mm.86382
230073	Ddx58	DEAD (Asp-Glu-Ala-Asp) box polypeptide 58	Mm.86382
234311	Ddx60	DEAD (Asp-Glu-Ala-Asp) box polypeptide 60	Mm.275233
234311	Ddx60	DEAD (Asp-Glu-Ala-Asp) box polypeptide 60	Mm.275233
320560	Dennd5b	DENN/MADD domain containing 5B	Mm.426874
97998	Depdc6	DEP domain containing 6	Mm.295397
97998	Depdc6	DEP domain containing 6	Mm.295397
97998	Depdc6	DEP domain containing 6	Mm.295397
97998	Depdc6	DEP domain containing 6	Mm.295397
13386	Dlk1	delta-like 1 homolog (Drosophila)	Mm.157069
13388	Dll1	delta-like 1 (Drosophila)	Mm.4875
327762	Dna2	DNA replication helicase 2 homolog (yeast)	Mm.21492
327762	Dna2	DNA replication helicase 2 homolog (yeast)	Mm.21492
110084	Dnahc1	dynein, axonemal, heavy chain 1	Mm.101811
327954	Dnahc2	dynein, axonemal, heavy chain 2	Mm.247478
58233	Dnaja4	DnaJ (Hsp40) homolog, subfamily A, member	Mm.28437
58233	Dnaja4	DnaJ (Hsp40) homolog, subfamily A, member	Mm.28437
58233	Dnaja4	DnaJ (Hsp40) homolog, subfamily A, member	Mm.28437
13429	Dnm1	dynamin 1	Mm.44736
13436	Dnmt3b	DNA methyltransferase 3B	Mm.89772
13436	Dnmt3b	DNA methyltransferase 3B	Mm.89772
244745	Dpy19l1	dpy-19-like 1 (C. elegans)	Mm.38526
244745	Dpy19l1	dpy-19-like 1 (C. elegans)	Mm.38526
233115	Dpy19l3	dpy-19-like 3 (C. elegans)	Mm.257808
233115	Dpy19l3	dpy-19-like 3 (C. elegans)	Mm.257808
99586	Dpyd	dihydropyrimidine dehydrogenase	Mm.27907
99586	Dpyd	dihydropyrimidine dehydrogenase	Mm.27907
99586	Dpyd	dihydropyrimidine dehydrogenase	Mm.27907
99586	Dpyd	dihydropyrimidine dehydrogenase	Mm.27907
99586	Dpyd	dihydropyrimidine dehydrogenase	Mm.27907
99586	Dpyd	dihydropyrimidine dehydrogenase	Mm.27907
72107	Dscc1	defective in sister chromatid cohesion 1 homo	Mm.277084
76843	Dtl	denticleless homolog (Drosophila)	Mm.189102
76843	Dtl	denticleless homolog (Drosophila)	Mm.189102
76843	Dtl	denticleless homolog (Drosophila)	Mm.189102
76843	Dtl	denticleless homolog (Drosophila)	Mm.189102
76843	Dtl	denticleless homolog (Drosophila)	Mm.189102
76843	Dtl	denticleless homolog (Drosophila)	Mm.189102
209200	Dtx3l	deltex 3-like (Drosophila)	Mm.390852
70686	Dusp16	dual specificity phosphatase 16	Mm.478037
70686	Dusp16	dual specificity phosphatase 16	Mm.478037
75590	Dusp9	dual specificity phosphatase 9	Mm.16479
68097	Dynll2	dynein light chain LC8-type 2	Mm.246436
68097	Dynll2	dynein light chain LC8-type 2	Mm.246436
224170	Dzip3	DAZ interacting protein 3, zinc finger	Mm.275138
224170	Dzip3	DAZ interacting protein 3, zinc finger	Mm.275138
224170	Dzip3	DAZ interacting protein 3, zinc finger	Mm.275138
50496	E2f6	E2F transcription factor 6	Mm.23296
13593	Ebf3	early B-cell factor 3	Mm.258708
13601	Ecm1	extracellular matrix protein 1	Mm.3433
19106	Eif2ak2	eukaryotic translation initiation factor 2-alpha I	Mm.378990
19106	Eif2ak2	eukaryotic translation initiation factor 2-alpha I	Mm.378990
192657	Ell2	elongation factor RNA polymerase II 2	Mm.21288
192657	Ell2	elongation factor RNA polymerase II 2	Mm.21288

13731	Emp2	epithelial membrane protein 2	Mm.246009
13731	Emp2	epithelial membrane protein 2	Mm.246009
13731	Emp2	epithelial membrane protein 2	Mm.246009
13731	Emp2	epithelial membrane protein 2	Mm.246009
13731	Emp2	epithelial membrane protein 2	Mm.246009
13731	Emp2	epithelial membrane protein 2	Mm.246009
13731	Emp2	epithelial membrane protein 2	Mm.246009
13731	Emp2	epithelial membrane protein 2	Mm.246009
18606	Enpp2	ectonucleotide pyrophosphatase/phosphodies	Mm.250256
18606	Enpp2	ectonucleotide pyrophosphatase/phosphodies	Mm.250256
105298	Epdr1	ependymin related protein 1 (zebrafish)	Mm.275054
105298	Epdr1	ependymin related protein 1 (zebrafish)	Mm.275054
13858	Eps15	epidermal growth factor receptor pathway sub	Mm.318250
13858	Eps15	epidermal growth factor receptor pathway sub	Mm.318250
13858	Eps15	epidermal growth factor receptor pathway sub	Mm.318250
13858	Eps15	epidermal growth factor receptor pathway sub	Mm.318250
13874	Ereg	epiregulin	Mm.4791
71151	Exod1	exonuclease domain containing 1	Mm.274160
226090	Ermp1	endoplasmic reticulum metallopeptidase 1	Mm.267131
71690	Esm1	endothelial cell-specific molecule 1	Mm.38929
75320	Etnk1	ethanolamine kinase 1	Mm.272548
75320	Etnk1	ethanolamine kinase 1	Mm.272548
26909	Exo1	exonuclease 1	Mm.283046
107371	Exoc6	exocyst complex component 6	Mm.24865
107371	Exoc6	exocyst complex component 6	Mm.24865
432940	Fam105b	family with sequence similarity 105, member E	Mm.309164
432940	Fam105b	Family with sequence similarity 105, member	Mm.309164
66540	Fam107b	family with sequence similarity 107, member E	Mm.277864
66540	Fam107b	family with sequence similarity 107, member E	Mm.277864
66540	Fam107b	family with sequence similarity 107, member E	Mm.277864
212326	Fam149a	family with sequence similarity 149, member /	Mm.51259
212326	Fam149a	family with sequence similarity 149, member /	Mm.51259
224093	Fam43a	family with sequence similarity 43, member A	Mm.28244
116972	Fam57a	family with sequence similarity 57, member A	Mm.188413
211651	Fancd2	Fanconi anemia, complementation group D2	Mm.160061
211651	Fancd2	Fanconi anemia, complementation group D2	Mm.160061
211651	Fancd2	Fanconi anemia, complementation group D2	Mm.160061
211651	Fancd2	Fanconi anemia, complementation group D2	Mm.160061
211651	Fancd2	Fanconi anemia, complementation group D2	Mm.160061
211651	Fancd2	Fanconi anemia, complementation group D2	Mm.160061
233079	Ffar2	free fatty acid receptor 2	Mm.97338
233079	Ffar2	free fatty acid receptor 2	Mm.97338
14164	Fgf1	fibroblast growth factor 1	Mm.241282
14164	Fgf1	fibroblast growth factor 1	Mm.241282
14164	Fgf1	fibroblast growth factor 1	Mm.241282
14164	Fgf1	fibroblast growth factor 1	Mm.241282
14167	Fgf12	fibroblast growth factor 12	Mm.7996
14194	Fh1	fumarate hydratase 1	Mm.41502
27055	Fkbp9	FK506 binding protein 9	Mm.20943
27055	Fkbp9	FK506 binding protein 9	Mm.20943
14257	Flt4	FMS-like tyrosine kinase 4	Mm.3291
108655	Foxp1	forkhead box P1	Mm.234965
108655	Foxp1	forkhead box P1	Mm.234965
108655	Foxp1	Forkhead box P1 (Foxp1), mRNA	Mm.234965
108655	Foxp1	forkhead box P1	Mm.234965
108655	Foxp1	forkhead box P1	Mm.234965
108655	Foxp1	forkhead box P1	Mm.234965
108655	Foxp1	forkhead box P1	Mm.234965
108655	Foxp1	forkhead box P1	Mm.234965
14373	G0s2	G0/G1 switch gene 2	Mm.439721
57436	Gabarapl1	gamma-aminobutyric acid (GABA) A receptor	Mm.14638
57436	Gabarapl1	gamma-aminobutyric acid (GABA) A receptor	Mm.14638
57436	Gabarapl1	gamma-aminobutyric acid (GABA) A receptor	Mm.14638
57436	Gabarapl1	gamma-aminobutyric acid (GABA) A receptor	Mm.14638
57436	Gabarapl1	gamma-aminobutyric acid (GABA) A receptor	Mm.14638
57436	Gabarapl1	gamma-aminobutyric acid (GABA) A receptor	Mm.14638
54393	Gabbr1	gamma-aminobutyric acid (GABA) B receptor	Mm.32191
54393	Gabbr1	gamma-aminobutyric acid (GABA) B receptor	Mm.32191
14402	Gabrb3	gamma-aminobutyric acid (GABA) A receptor	Mm.8004
14402	Gabrb3	gamma-aminobutyric acid (GABA) A receptor	Mm.8004
108150	Galnt7	UDP-N-acetyl-alpha-D-galactosamine: polype	Mm.62886
108150	Galnt7	UDP-N-acetyl-alpha-D-galactosamine: polype	Mm.62886
108150	Galnt7	UDP-N-acetyl-alpha-D-galactosamine: polype	Mm.62886
380711	Garnl4	GTPase activating RANGAP domain-like 4	Mm.425690
14457	Gas7	growth arrest specific 7	Mm.40338
14457	Gas7	growth arrest specific 7	Mm.40338
14457	Gas7	growth arrest specific 7	Mm.40338
14461	Gata2	GATA binding protein 2	Mm.476843
14461	Gata2	GATA binding protein 2	Mm.476843
55932	Gbp3	guanylate binding protein 3	Mm.439718
67242	Gemin6	gem (nuclear organelle) associated protein 6	Mm.279985
109801	Glo1	glyoxalase 1	Mm.261984
109801	Glo1	glyoxalase 1	Mm.261984
109801	Glo1	glyoxalase 1	Mm.261984
432486	Gnptab	N-acetylglucosamine-1-phosphate transferase	Mm.335190
14719	Got2	glutamate oxaloacetate transaminase 2, mitoc	Mm.230169
67133	Gp2	glycoprotein 2 (zymogen granule membrane)	Mm.46403
67549	Gpr89	G protein-coupled receptor 89	Mm.46722
67549	Gpr89	G protein-coupled receptor 89	Mm.46722
67549	Gpr89	G protein-coupled receptor 89	Mm.46722
67549	Gpr89	G protein-coupled receptor 89	Mm.46722
69590	Gpx8	glutathione peroxidase 8 (putative)	Mm.12715
50915	Grb14	growth factor receptor bound protein 14	Mm.214554
0045413 /// 2685	Greb1 /// LOC1	gene regulated by estrogen in breast cancer p	Mm.466550
14866	Gstm5	glutathione S-transferase, mu 5	Mm.282351
14865	Gstm4	glutathione S-transferase, mu 4	Mm.31203
269252	Gtf3c4	general transcription factor IIIC, polypeptide 4	Mm.26043
74174	Gtsf1	Gametocyte specific factor 1 (Gtsf1), mRNA	Mm.325325
54195	Gucy1b3	guanylate cyclase 1, soluble, beta 3	Mm.9445
54195	Gucy1b3	guanylate cyclase 1, soluble, beta 3	Mm.9445
110257 /// 1512	Hba-a1 /// Hba-	hemoglobin alpha, adult chain 1 /// hemoglobi	Mm.196110
110257 /// 1512	Hba-a1 /// Hba-	hemoglobin alpha, adult chain 1 /// hemoglobi	Mm.196110
110257 /// 1512	Hba-a1 /// Hba-	hemoglobin alpha, adult chain 1 /// hemoglobi	Mm.196110
15135	Hbb-y	hemoglobin Y, beta-like embryonic chain	Mm.440164
15135	Hbb-y	hemoglobin Y, beta-like embryonic chain	Mm.440164
15135	Hbb-y	hemoglobin Y, beta-like embryonic chain	Mm.440164
15200	Hbegf	heparin-binding EGF-like growth factor	Mm.289681
15200	Hbegf	heparin-binding EGF-like growth factor	Mm.289681
15132	Hbb-bh1	hemoglobin Z, beta-like embryonic chain	Mm.440281
15132	Hbb-bh1	hemoglobin Z, beta-like embryonic chain	Mm.440281
52897	D11Bwg0517e	DNA segment, Chr 11, Brigham & Women's (	Mm.341103
52897	D11Bwg0517e	DNA segment, Chr 11, Brigham & Women's (	Mm.341103
15001	H2-Oa	histocompatibility 2, O region alpha locus	Mm.116
14961	H2-Ab1	histocompatibility 2, class II antigen A, beta 1	Mm.254067
14961	H2-Ab1	histocompatibility 2, class II antigen A, beta 1	Mm.254067
14961	H2-Ab1	histocompatibility 2, class II antigen A, beta 1	Mm.254067
14968	H2-Ea	histocompatibility 2, class II antigen E alpha	Mm.15680
15366	Hmmr	hyaluronan mediated motility receptor (RHAM	Mm.116997
15366	Hmmr	hyaluronan mediated motility receptor (RHAM	Mm.116997
15366	Hmmr	hyaluronan mediated motility receptor (RHAM	Mm.116997
15366	Hmmr	hyaluronan mediated motility receptor (RHAM	Mm.116997
15384	Hnrnpab	heterogeneous nuclear ribonucleoprotein A/B	Mm.280842
15384	Hnrnpab	heterogeneous nuclear ribonucleoprotein A/B	Mm.280842
15384	Hnrnpab	heterogeneous nuclear ribonucleoprotein A/B	Mm.280842
15384	Hnrnpab	heterogeneous nuclear ribonucleoprotein A/B	Mm.280842
15384	Hnrnpab	heterogeneous nuclear ribonucleoprotein A/B	Mm.280842
15519	Hsp90aa1	heat shock protein 90, alpha (cytosolic), class	Mm.1843
15519	Hsp90aa1	heat shock protein 90, alpha (cytosolic), class	Mm.1843
15519	Hsp90aa1	heat shock protein 90, alpha (cytosolic), class	Mm.1843
15519	Hsp90aa1	heat shock protein 90, alpha (cytosolic), class	Mm.1843
15519	Hsp90aa1	heat shock protein 90, alpha (cytosolic), class	Mm.1843
15519	Hsp90aa1	heat shock protein 90, alpha (cytosolic), class	Mm.1843
15519	Hsp90aa1	heat shock protein 90, alpha (cytosolic), class	Mm.1843
15519	Hsp90aa1	heat shock protein 90, alpha (cytosolic), class	Mm.1843
15874	Iapp	islet amyloid polypeptide	Mm.415
15874	Iapp	islet amyloid polypeptide	Mm.415
15874	Iapp	islet amyloid polypeptide	Mm.415
15874	Iapp	islet amyloid polypeptide	Mm.415
15903	Id3	inhibitor of DNA binding 3	Mm.110
15931	Ids	iduronate 2-sulfatase	Mm.233083
15931	Ids	iduronate 2-sulfatase	Mm.233083
15931	Ids	iduronate 2-sulfatase	Mm.233083

15931	Ids	iduronate 2-sulfatase	Mm.233083
15931	Ids	iduronate 2-sulfatase	Mm.233083
15931	Ids	iduronate 2-sulfatase	Mm.233083
15931	Ids	iduronate 2-sulfatase	Mm.233083
15931	Ids	iduronate 2-sulfatase	Mm.233083
52668	Ifi27l1	interferon, alpha-inducible protein 27 like 1	Mm.2121
52668	Ifi27l1	interferon, alpha-inducible protein 27 like 1	Mm.2121
99899	Ifi44	interferon-induced protein 44	Mm.30756
99899	Ifi44	interferon-induced protein 44	Mm.30756
71586	Ifih1	interferon induced with helicase C domain 1	Mm.136224
71586	Ifih1	interferon induced with helicase C domain 1	Mm.136224
71586	Ifih1	interferon induced with helicase C domain 1	Mm.136224
667373	OTTMUSG000	predicted gene, OTTMUSG00000016644	Mm.371956
112419	2010002M12Ri	RIKEN cDNA 2010002M12 gene	Mm.440727
15958	Ifit2	interferon-induced protein with tetratricopeptic	Mm.2036
15959	Ifit3	interferon-induced protein with tetratricopeptic	Mm.426079
15959	Ifit3	interferon-induced protein with tetratricopeptic	Mm.426079
16002	Igf2	insulin-like growth factor 2	Mm.3862
16006	Igfbp1	insulin-like growth factor binding protein 1	Mm.21300
16009	Igfbp3	insulin-like growth factor binding protein 3	Mm.29254
16009	Igfbp3	insulin-like growth factor binding protein 3	Mm.29254
16009	Igfbp3	insulin-like growth factor binding protein 3	Mm.29254
16009	Igfbp3	insulin-like growth factor binding protein 3	Mm.29254
16011	Igfbp5	insulin-like growth factor binding protein 5	Mm.405761
16012	Igfbp6	insulin-like growth factor binding protein 6	Mm.358609
60504	Il21r	interleukin 21 receptor	Mm.155643
16195	Il6st	interleukin 6 signal transducer	Mm.4364
16195	Il6st	interleukin 6 signal transducer	Mm.4364
242291	Impad1	inositol monophosphatase domain containing	Mm.218889
242291	Impad1	inositol monophosphatase domain containing	Mm.218889
242291	Impad1	inositol monophosphatase domain containing	Mm.218889
242291	Impad1	inositol monophosphatase domain containing	Mm.218889
242291	Impad1	inositol monophosphatase domain containing	Mm.218889
242291	Impad1	inositol monophosphatase domain containing	Mm.218889
242291	Impad1	inositol monophosphatase domain containing	Mm.218889
242291	Impad1	inositol monophosphatase domain containing	Mm.218889
242291	Impad1	inositol monophosphatase domain containing	Mm.218889
76582	Ipo11	importin 11	Mm.132208
76582	Ipo11	importin 11	Mm.132208
226432	Ipo9	importin 9	Mm.271642
226432	Ipo9	importin 9	Mm.271642
232227	Iqsec1	IQ motif and Sec7 domain 1	Mm.196943
232227	Iqsec1	IQ motif and Sec7 domain 1	Mm.196943
270110	Irf2bp2	interferon regulatory factor 2 binding protein 2	Mm.334918
270110	Irf2bp2	interferon regulatory factor 2 binding protein 2	Mm.334918
270110	Irf2bp2	interferon regulatory factor 2 binding protein 2	Mm.334918
270110	Irf2bp2	interferon regulatory factor 2 binding protein 2	Mm.334918
270110	Irf2bp2	interferon regulatory factor 2 binding protein 2	Mm.334918
270110	Irf2bp2	interferon regulatory factor 2 binding protein 2	Mm.334918
54123	Irf7	interferon regulatory factor 7	Mm.3233
0038882 /// 677	677168 /// Isg1	predicted gene, 677168 /// ISG15 ubiquitin-lik	Mm.4950
16392	Isl1	ISL1 transcription factor, LIM/homeodomain	Mm.42242
16392	Isl1	ISL1 transcription factor, LIM/homeodomain	Mm.42242
16426	Itih3	inter-alpha trypsin inhibitor, heavy chain 3	Mm.4517
16438	Itpr1	inositol 1,4,5-triphosphate receptor 1	Mm.227912
16438	Itpr1	inositol 1,4,5-triphosphate receptor 1	Mm.227912
76217	Jakmip2	janus kinase and microtubule interacting prote	Mm.134602
545156	Kalrn	kalirin, RhoGEF kinase	Mm.401884
545156	Kalrn	kalirin, RhoGEF kinase	Mm.401884
545156	Kalrn	kalirin, RhoGEF kinase	Mm.401884
545156	Kalrn	kalirin, RhoGEF kinase	Mm.401884
56543	Kcnd3	potassium voltage-gated channel, Shal-relatec	Mm.44530
67498	Kcnv1	potassium channel, subfamily V, member 1	Mm.300079
75605	Kdm5b	lysine (K)-specific demethylase 5B	Mm.28995
75605	Kdm5b	lysine (K)-specific demethylase 5B	Mm.28995
75605	Kdm5b	lysine (K)-specific demethylase 5B	Mm.28995
75605	Kdm5b	lysine (K)-specific demethylase 5B	Mm.28995
75605	Kdm5b	lysine (K)-specific demethylase 5B	Mm.28995
75605	Kdm5b	lysine (K)-specific demethylase 5B	Mm.28995
75605	Kdm5b	lysine (K)-specific demethylase 5B	Mm.28995
75605	Kdm5b	lysine (K)-specific demethylase 5B	Mm.28995
75605	Kdm5b	lysine (K)-specific demethylase 5B	Mm.28995
71955	2400003C14Ril	RIKEN cDNA 2400003C14 gene	Mm.290036
71955	2400003C14Ril	RIKEN cDNA 2400003C14 gene	Mm.290036
217732	2310044G17Ri	RIKEN cDNA 2310044G17 gene	Mm.22337
217732	2310044G17Ri	RIKEN cDNA 2310044G17 gene	Mm.22337
217732	2310044G17Ri	RIKEN cDNA 2310044G17 gene	Mm.22337
16551	Kif11	kinesin family member 11	Mm.42203
16551	Kif11	kinesin family member 11	Mm.42203
240641	Kif20b	kinesin family member 20B	Mm.278471
240641	Kif20b	kinesin family member 20B	Mm.278471
16563	Kif2a	kinesin family member 2A	Mm.355686
16563	Kif2a	kinesin family member 2A	Mm.355686
0046855 /// 165	Klf3 /// LOC100	Kruppel-like factor 3 (basic) /// similar to BKLI	Mm.439720
0046855 /// 165	Klf3 /// LOC100	Kruppel-like factor 3 (basic) /// similar to BKLI	Mm.439720
16599	Klf3	Kruppel-like factor 3 (basic)	Mm.439720
76788	Klhdc10	kelch domain containing 10	Mm.277308
76788	Klhdc10	kelch domain containing 10	Mm.277308
18048	Klk1b4	kallikrein 1-related pepidase b4	Mm.440886
16617	Klk1b24	kallikrein 1-related peptidase b24	Mm.444308
18050	Klk1b3	kallikrein 1-related peptidase b3	Mm.439740
18048	Klk1b4	kallikrein 1-related pepidase b4	Mm.440886
16622	Klk1b5	kallikrein 1-related peptidase b5	Mm.443373
16623	Klk1b1	kallikrein 1-related peptidase b1	Mm.447819
13646 /// 13648	Klk1b22 /// Klk1	kallikrein 1-related peptidase b22 /// kallikrein	Mm.440884
16622	Klk1b5	kallikrein 1-related peptidase b5	Mm.443373
16624	Klk1b8	kallikrein 1-related peptidase b8	Mm.435488
16615	Klk1b16	kallikrein 1-related peptidase b16	Mm.440887
16613	Klk1b11	kallikrein 1-related peptidase b11	Mm.443378
16618	Klk1b26	kallikrein 1-related petidase b26	Mm.439663
16616	Klk1b21	kallikrein 1-related peptidase b21	Mm.443292
16619	Klk1b27	kallikrein 1-related peptidase b27	Mm.445911
68668	Klk5	kallikrein related-peptidase 5	Mm.458122
23993	Klk7	kallikrein related-peptidase 7 (chymotryptic, st	Mm.251227
208628	Kntc1	kinetochore associated 1	Mm.441089
16663	Krt13	keratin 13	Mm.4646
16663	Krt13	keratin 13	Mm.4646
16663	Krt13	keratin 13	Mm.4646
16666	Krt16	keratin 16	Mm.422799
16679	Krt86	keratin 86	Mm.383183
16706	Ksr1	kinase suppressor of ras 1	Mm.4745
16706	Ksr1	kinase suppressor of ras 1	Mm.4745
381591	L1td1	LINE-1 type transposase domain containing 1	Mm.34340
237339	L3mbtl3	l(3)mbt-like 3 (Drosophila)	Mm.338439
237339	L3mbtl3	l(3)mbt-like 3 (Drosophila)	Mm.338439
237339	L3mbtl3	l(3)mbt-like 3 (Drosophila)	Mm.338439
66988	Lap3	leucine aminopeptidase 3	Mm.286830
16819	Lcn2	lipocalin 2	Mm.9537
320202	Lefty2	left-right determination factor 2	Mm.87078
231506	Lin54	lin-54 homolog (C. elegans)	Mm.212568
74488	Lrrc15	leucine rich repeat containing 15	Mm.290677
71738	Mamdc2	MAM domain containing 2	Mm.50841
67216	Mboat2	membrane bound O-acyltransferase domain c	Mm.167671
67216	Mboat2	membrane bound O-acyltransferase domain c	Mm.167671
17219	Mcm6	minichromosome maintenance deficient 6 (MI	Mm.4933
17219	Mcm6	minichromosome maintenance deficient 6 (MI	Mm.4933
76199	Med13l	mediator complex subunit 13-like	Mm.206238
76199	Med13l	mediator complex subunit 13-like	Mm.206238
76199	Med13l	mediator complex subunit 13-like	Mm.206238
30060	Mfi2	antigen p97 (melanoma associated) identified	Mm.30988
69674	Mif4gd	MIF4G domain containing	Mm.390387
17345	Mki67	antigen identified by monoclonal antibody Ki 6	Mm.4078
17345	Mki67	antigen identified by monoclonal antibody Ki 6	Mm.4078
27418	Mkln1	muskelin 1, intracellular mediator containing k	Mm.238038
54484	Mkrn1	makorin, ring finger protein, 1	Mm.270484
74568	Mlkl	mixed lineage kinase domain-like	Mm.207971
17395	Mmp9	matrix metallopeptidase 9	Mm.4406
17395	Mmp9	matrix metallopeptidase 9	Mm.4406
433938	Mn1	meningioma 1	Mm.332576
225651	Mppe1	metallophosphoesterase 1	Mm.235792
225651	Mppe1	metallophosphoesterase 1	Mm.235792
225651	Mppe1	metallophosphoesterase 1	Mm.235792

68481	Mpzl1	myelin protein zero-like 1	Mm.46438
68481	Mpzl1	myelin protein zero-like 1	Mm.46438
68481	Mpzl1	myelin protein zero-like 1	Mm.46438
68481	Mpzl1	myelin protein zero-like 1	Mm.46438
68481	Mpzl1	myelin protein zero-like 1	Mm.46438
68481	Mpzl1	myelin protein zero-like 1	Mm.46438
665563	Mthfd2l	methylenetetrahydrofolate dehydrogenase (NA	Mm.153030
665563	Mthfd2l	methylenetetrahydrofolate dehydrogenase (NA	Mm.153030
665563	Mthfd2l	methylenetetrahydrofolate dehydrogenase (NA	Mm.153030
665563	Mthfd2l	methylenetetrahydrofolate dehydrogenase (NA	Mm.153030
665563	Mthfd2l	methylenetetrahydrofolate dehydrogenase (NA	Mm.153030
665563	Mthfd2l	methylenetetrahydrofolate dehydrogenase (NA	Mm.153030
338367	Myo1d	myosin ID	Mm.151948
338367	Myo1d	myosin ID	Mm.151948
17933	Myt1l	myelin transcription factor 1-like	Mm.253067
17933	Myt1l	myelin transcription factor 1-like	Mm.253067
17933	Myt1l	myelin transcription factor 1-like	Mm.253067
72560	Naalad2	N-acetylated alpha-linked acidic dipeptidase 2	Mm.474510
78658	Ncapd3	non-SMC condensin II complex, subunit D3	Mm.251777
78658	Ncapd3	non-SMC condensin II complex, subunit D3	Mm.251777
18005	Nek2	NIMA (never in mitosis gene a)-related expres	Mm.33773
18005	Nek2	NIMA (never in mitosis gene a)-related expres	Mm.33773
18028	Nfib	nuclear factor I/B	Mm.317947
18028	Nfib	nuclear factor I/B	Mm.317947
18028	Nfib	nuclear factor I/B	Mm.317947
18028	Nfib	nuclear factor I/B	Mm.317947
18028	Nfib	nuclear factor I/B	Mm.317947
18053	Ngfr	nerve growth factor receptor (TNFR superfam	Mm.283893
66866	Nhlrc2	NHL repeat containing 2	Mm.392509
66866	Nhlrc2	NHL repeat containing 2	Mm.46346
233280	Nipa1	non imprinted in Prader-Willi/Angelman syndr	Mm.389901
233280	Nipa1	non imprinted in Prader-Willi/Angelman syndr	Mm.389901
67711	Nsmce1	Non-SMC element 1 homolog (S. cerevisiae),	Mm.4467
67711	Nsmce1	non-SMC element 1 homolog (S. cerevisiae)	Mm.4467
70699	Nup205	nucleoporin 205	Mm.261208
108907	Nusap1	nucleolar and spindle associated protein 1	Mm.290015
246730	Oas1a	2'-5' oligoadenylate synthetase 1A	Mm.14301
246728	Oas2	2'-5' oligoadenylate synthetase 2	Mm.260926
246728	Oas2	2'-5' oligoadenylate synthetase 2	Mm.260926
56177	Olfm1	olfactomedin 1	Mm.43278
56177	Olfm1	olfactomedin 1	Mm.43278
56177	Olfm1	olfactomedin 1	Mm.43278
18406	Orm2	orosomucoid 2	Mm.14173
18405	Orm1	orosomucoid 1	Mm.4777
18600	Padi2	peptidyl arginine deiminase, type II	Mm.2296
18600	Padi2	peptidyl arginine deiminase, type II	Mm.2296
18602	Padi4	peptidyl arginine deiminase, type IV	Mm.250358
243771	Parp12	poly (ADP-ribose) polymerase family, member	Mm.268462
547253	Parp14	poly (ADP-ribose) polymerase family, member	Mm.244406
547253	Parp14	poly (ADP-ribose) polymerase family, member	Mm.244406
269224	Pask	PAS domain containing serine/threonine kinas	Mm.379454
269224	Pask	PAS domain containing serine/threonine kinas	Mm.379454
269224	Pask	PAS domain containing serine/threonine kinas	Mm.379454
52033	Pbk	PDZ binding kinase	Mm.24337
69587	Pcgf3	polycomb group ring finger 3	Mm.261218
54604	Pcnx	pecanex homolog (Drosophila)	Mm.86584
54604	Pcnx	pecanex homolog (Drosophila)	Mm.86584
54604	Pcnx	pecanex homolog (Drosophila)	Mm.86584
54604	Pcnx	pecanex homolog (Drosophila)	Mm.86584
18584	Pde8a	phosphodiesterase 8A	Mm.458069
18584	Pde8a	phosphodiesterase 8A	Mm.458069
18610	Pdyn	prodynorphin	Mm.6239
111175	Pecr	peroxisomal trans-2-enoyl-CoA reductase	Mm.281738
111175	Pecr	peroxisomal trans-2-enoyl-CoA reductase	Mm.281738
72129	Pex13	peroxisomal biogenesis factor 13	Mm.286622
72129	Pex13	peroxisomal biogenesis factor 13	Mm.286622
72157	Pgm2	phosphoglucomutase 2	Mm.217764
72157	Pgm2	phosphoglucomutase 2	Mm.217764
215789	Phactr2	phosphatase and actin regulator 2	Mm.338311
21664	Phlda1	pleckstrin homology-like domain, family A, me	Mm.3117
68770	Phtf2	putative homeodomain transcription factor 2	Mm.86410
68770	Phtf2	Putative homeodomain transcription factor 2,	Mm.86410
68770	Phtf2	putative homeodomain transcription factor 2	Mm.86410
18718	Pip4k2a	Phosphatidylinositol-5-phosphate 4-kinase, ty	Mm.313977
18718	Pip4k2a	phosphatidylinositol-5-phosphate 4-kinase, ty	Mm.313977
18718	Pip4k2a	phosphatidylinositol-5-phosphate 4-kinase, ty	Mm.313977
18718	Pip4k2a	phosphatidylinositol-5-phosphate 4-kinase, ty	Mm.313977
18741	Pitx2	paired-like homeodomain transcription factor 2	Mm.246804
18795	Plcb1	phospholipase C, beta 1	Mm.330607
20873	Plk4	polo-like kinase 4 (Drosophila)	Mm.3794
20873	Plk4	polo-like kinase 4 (Drosophila)	Mm.3794
20873	Plk4	polo-like kinase 4 (Drosophila)	Mm.3794
20873	Plk4	polo-like kinase 4 (Drosophila)	Mm.3794
26432	Plod2	procollagen lysine, 2-oxoglutarate 5-dioxygena	Mm.79983
26432	Plod2	procollagen lysine, 2-oxoglutarate 5-dioxygena	Mm.79983
56695	Pnkd	paroxysmal nonkinesiogenic dyskinesia	Mm.384726
241274	Pnpla7	patatin-like phospholipase domain containing	Mm.389243
69693	Pof1b	premature ovarian failure 1B	Mm.34964
18973	Pole	polymerase (DNA directed), epsilon	Mm.35061
233406	Prc1	protein regulator of cytokinesis 1	Mm.227274
233406	Prc1	protein regulator of cytokinesis 1	Mm.227274
18477	Prdx1	peroxiredoxin 1	Mm.30929
18477 /// 63727	EG637273 /// P	predicted gene, EG637273 /// peroxiredoxin 1	Mm.30929
18477	Prdx1	peroxiredoxin 1	Mm.30929
18477	Prdx1	peroxiredoxin 1	Mm.30929
18477	Prdx1	peroxiredoxin 1	Mm.30929
116847	Prelp	proline arginine-rich end leucine-rich repeat	Mm.214514
18750	Prkca	protein kinase C, alpha	Mm.222178
18750	Prkca	protein kinase C, alpha	Mm.222178
212627	Prpsap2	phosphoribosyl pyrophosphate synthetase-as	Mm.27703
19146	Prss7	protease, serine, 7 (enterokinase)	Mm.5184
234353	Psd3	pleckstrin and Sec7 domain containing 3	Mm.32525
234353	Psd3	pleckstrin and Sec7 domain containing 3	Mm.32525
69077	Psmd11	proteasome (prosome, macropain) 26S subur	Mm.260539
69077	Psmd11	Proteasome (prosome, macropain) 26S subu	Mm.260539
69077	Psmd11	proteasome (prosome, macropain) 26S subur	Mm.260539
19211	Pten	phosphatase and tensin homolog	Mm.245395
19211	Pten	phosphatase and tensin homolog	Mm.245395
19218	Ptger3	prostaglandin E receptor 3 (subtype EP3)	Mm.30424
19218	Ptger3	prostaglandin E receptor 3 (subtype EP3)	Mm.30424
19222	Ptgir	prostaglandin I receptor (IP)	Mm.287572
67103	Ptgr1	prostaglandin reductase 1	Mm.34497
67103	Ptgr1	prostaglandin reductase 1	Mm.34497
77219	Ptgr2	prostaglandin reductase 2	Mm.246127
77219	Ptgr2	prostaglandin reductase 2	Mm.246127
217057	Ptrh2	peptidyl-tRNA hydrolase 2	Mm.24868
69675	Pxdn	peroxidasin homolog (Drosophila)	Mm.251774
69675	Pxdn	peroxidasin homolog (Drosophila)	Mm.251774
99003	Qser1	glutamine and serine rich 1	Mm.274314
99003	Qser1	glutamine and serine rich 1	Mm.274314
99003	Qser1	glutamine and serine rich 1	Mm.274314
99003	Qser1	glutamine and serine rich 1	Mm.274314
99003	Qser1	glutamine and serine rich 1	Mm.274314
99003	Qser1	glutamine and serine rich 1	Mm.274314
215445	Rab11fip3	RAB11 family interacting protein 3 (class II)	Mm.41191
19335	Rab23	RAB23, member RAS oncogene family	Mm.86744
216363	Rab3ip	RAB3A interacting protein	Mm.336394
216363	Rab3ip	RAB3A interacting protein	Mm.336394
19354	Rac2	RAS-related C3 botulinum substrate 2	Mm.1972
19354	Rac2	RAS-related C3 botulinum substrate 2	Mm.1972
241308	Ralgps1	Ral GEF with PH domain and SH3 binding m	Mm.274249
241308	Ralgps1	Ral GEF with PH domain and SH3 binding m	Mm.274249
19701 /// 19702	Ren1 /// Ren2	renin 1 structural /// renin 2 tandem duplicatio	Mm.220955
194590	Reps2	RALBP1 associated Eps domain containing p	Mm.442441
19735	Rgs2	regulator of G-protein signaling 2	Mm.28262
19735	Rgs2	regulator of G-protein signaling 2	Mm.28262
69288	Rhobtb1	Rho-related BTB domain containing 1	Mm.26659
69288	Rhobtb1	Rho-related BTB domain containing 1	Mm.26659
237422	Ric8b	resistance to inhibitors of cholinesterase 8 ho	Mm.18936
108653	Rimklb	ribosomal modification protein rimK-like family	Mm.277939
108653	Rimklb	ribosomal modification protein rimK-like family	Mm.277939
108653	Rimklb	ribosomal modification protein rimK-like family	Mm.277939

54159	Ear5	eosinophil-associated, ribonuclease A family,	Mm.377125
14000	Rnasen	ribonuclease III, nuclear	Mm.293142
26563	Ror1	receptor tyrosine kinase-like orphan receptor 1	Mm.57252
67071	Rps6ka6	ribosomal protein S6 kinase polypeptide 6	Mm.37617
58185	Rsad2	radical S-adenosyl methionine domain contair	Mm.24045
58185	Rsad2	radical S-adenosyl methionine domain contair	Mm.24045
58185	Rsad2	radical S-adenosyl methionine domain contair	Mm.24045
58185	Rsad2	radical S-adenosyl methionine domain contair	Mm.24045
58185	Rsad2	radical S-adenosyl methionine domain contair	Mm.24045
58185	Rsad2	radical S-adenosyl methionine domain contair	Mm.24045
67775	Rtp4	receptor transporter protein 4	Mm.475107
67775	Rtp4	receptor transporter protein 4	Mm.475107
20203	S100b	S100 protein, beta polypeptide, neural	Mm.235998
20203	S100b	S100 protein, beta polypeptide, neural	Mm.235998
13610	S1pr3	sphingosine-1-phosphate receptor 3	Mm.136736
20210	Saa3	serum amyloid A 3	Mm.14277
20210	Saa3	serum amyloid A 3	Mm.14277
20208	Saa1	serum amyloid A 1	Mm.148800
20208	Saa1	serum amyloid A 1	Mm.148800
20208	Saa1	serum amyloid A 1	Mm.148800
20208	Saa1	serum amyloid A 1	Mm.148800
20209	Saa2	serum amyloid A 2	Mm.200941
20209	Saa2	serum amyloid A 2	Mm.200941
56459	Sae1	SUMO1 activating enzyme subunit 1	Mm.258530
56459	Sae1	SUMO1 activating enzyme subunit 1	Mm.258530
56459	Sae1	SUMO1 activating enzyme subunit 1	Mm.258530
72776	Sass6	spindle assembly 6 homolog (C. elegans)	Mm.440836
72776	Sass6	spindle assembly 6 homolog (C. elegans)	Mm.440836
20274	Scn9a	sodium channel, voltage-gated, type IX, alpha	Mm.440889
20274	Scn9a	sodium channel, voltage-gated, type IX, alpha	Mm.440889
20274	Scn9a	sodium channel, voltage-gated, type IX, alpha	Mm.440889
20274	Scn9a	sodium channel, voltage-gated, type IX, alpha	Mm.440889
20274	Scn9a	sodium channel, voltage-gated, type IX, alpha	Mm.440889
20274	Scn9a	sodium channel, voltage-gated, type IX, alpha	Mm.440889
20341	Selenbp1	selenium binding protein 1	Mm.196558
20341	Selenbp1	selenium binding protein 1	Mm.196558
20355	Sema4f	sema domain, immunoglobulin domain (Ig), T	Mm.270543
20355	Sema4f	sema domain, immunoglobulin domain (Ig), T	Mm.270543
20355	Sema4f	sema domain, immunoglobulin domain (Ig), T	Mm.270543
20355	Sema4f	sema domain, immunoglobulin domain (Ig), T	Mm.270543
109079	Sephs1	selenophosphate synthetase 1	Mm.34329
109079	Sephs1	selenophosphate synthetase 1	Mm.34329
109079	Sephs1	selenophosphate synthetase 1	Mm.34329
109079	Sephs1	selenophosphate synthetase 1	Mm.34329
109079	Sephs1	selenophosphate synthetase 1	Mm.34329
109079	Sephs1	selenophosphate synthetase 1	Mm.34329
53860	Sept 9	septin 9	Mm.38450
20725	Serpinb8	serine (or cysteine) peptidase inhibitor, clade	Mm.83909
20725	Serpinb8	serine (or cysteine) peptidase inhibitor, clade	Mm.83909
20389	Sftpc	surfactant associated protein C	Mm.24040
74442	Sgms2	sphingomyelin synthase 2	Mm.273360
74442	Sgms2	sphingomyelin synthase 2	Mm.273360
230126	Shb	src homology 2 domain-containing transformi	Mm.251716
27401	Skp2	S-phase kinase-associated protein 2 (p45)	Mm.35584
27401	Skp2	S-phase kinase-associated protein 2 (p45)	Mm.35584
27401	Skp2	S-phase kinase-associated protein 2 (p45)	Mm.35584
27401	Skp2	S-phase kinase-associated protein 2 (p45)	Mm.35584
27401	Skp2	S-phase kinase-associated protein 2 (p45)	Mm.35584
27401	Skp2	S-phase kinase-associated protein 2 (p45)	Mm.35584
27401	Skp2	S-phase kinase-associated protein 2 (p45)	Mm.35584
27401	Skp2	S-phase kinase-associated protein 2 (p45)	Mm.35584
27218	Slamf1	signaling lymphocytic activation molecule fam	Mm.103648
27218	Slamf1	signaling lymphocytic activation molecule fam	Mm.103648
18174	Slc11a2	solute carrier family 11 (proton-coupled divale	Mm.234608
20496	Slc12a2	solute carrier family 12, member 2	Mm.399997
20496	Slc12a2	solute carrier family 12, member 2	Mm.399997
20496	Slc12a2	solute carrier family 12, member 2	Mm.399997
20510	Slc1a1	solute carrier family 1 (neuronal/epithelial high	Mm.246670
20510	Slc1a1	solute carrier family 1 (neuronal/epithelial high	Mm.246670
72144	Slc37a3	solute carrier family 37 (glycerol-3-phosphate	Mm.277527
72144	Slc37a3	solute carrier family 37 (glycerol-3-phosphate	Mm.277527
268706	Slc38a9	solute carrier family 38, member 9	Mm.259799
268706	Slc38a9	solute carrier family 38, member 9	Mm.259799
67547	Slc39a8	solute carrier family 39 (metal ion transporter)	Mm.30239
71699	Slc41a3	solute carrier family 41, member 3	Mm.272633
71699	Slc41a3	solute carrier family 41, member 3	Mm.272633
58207	Slc43a3	solute carrier family 43, member 3	Mm.290729
212980	Slc45a3	solute carrier family 45, member 3	Mm.200307
52466	Slc46a1	solute carrier family 46, member 1	Mm.131618
20533	Slc4a1	solute carrier family 4 (anion exchanger), men	Mm.7248
20533	Slc4a1	solute carrier family 4 (anion exchanger), men	Mm.7248
20538	Slc6a2	solute carrier family 6 (neurotransmitter transı	Mm.57040
102680	Slc6a20a	solute carrier family 6 (neurotransmitter transı	Mm.440867
20558	Slfn4	schlafen 4	Mm.38192
20568	Slpi	secretory leukocyte peptidase inhibitor	Mm.371583
57277	Slurp1	secreted Ly6/Plaur domain containing 1	Mm.27630
93761	Smarca1	SWI/SNF related, matrix associated, actin de	Mm.229151
93761	Smarca1	SWI/SNF related, matrix associated, actin de	Mm.229151
20646 /// 84704	Snrpn /// Snurf	small nuclear ribonucleoprotein N /// SNRPN	Mm.274995
20646 /// 54506	EG545062 /// S	predicted gene, EG545062 /// small nuclear ri	Mm.274995
20646 /// 84704	Snrpn /// Snurf	small nuclear ribonucleoprotein N /// SNRPN	Mm.274995
20646	Snrpn	small nuclear ribonucleoprotein N	Mm.274995
20646 /// 84704	Snrpn /// Snurf	small nuclear ribonucleoprotein N /// SNRPN	Mm.274995
20646 /// 54506	EG545062 /// S	predicted gene, EG545062 /// small nuclear ri	Mm.274995
20646 /// 84704	Snrpn /// Snurf	small nuclear ribonucleoprotein N /// SNRPN	Mm.274995
20646	Snrpn	small nuclear ribonucleoprotein N	Mm.274995
71982	Snx10	sorting nexin 10	Mm.476868
71982	Snx10	Sorting nexin 10 (Snx10), transcript variant 1,	Mm.417373 /// Mm.476868
72572	Spats2	spermatogenesis associated, serine-rich 2	Mm.276650
20750	Spp1	secreted phosphoprotein 1	Mm.288474
20742	Spnb2	spectrin beta 2	Mm.123110
20742	Spnb2	spectrin beta 2	Mm.123110
20742	Spnb2	spectrin beta 2	Mm.123110
20742	Spnb2	spectrin beta 2	Mm.123110
20742	Spnb2	spectrin beta 2	Mm.123110
59010	Sqrdl	sulfide quinone reductase-like (yeast)	Mm.28986
54613	St3gal6	ST3 beta-galactoside alpha-2,3-sialyltransfera	Mm.212742
54613	St3gal6	ST3 beta-galactoside alpha-2,3-sialyltransfera	Mm.212742
54613	St3gal6	ST3 beta-galactoside alpha-2,3-sialyltransfera	Mm.212742
54613	St3gal6	ST3 beta-galactoside alpha-2,3-sialyltransfera	Mm.212742
20840	Stac	src homology three (SH3) and cysteine rich d	Mm.1414
20846	Stat1	signal transducer and activator of transcriptio	Mm.277406
20846	Stat1	signal transducer and activator of transcriptio	Mm.277406
20846	Stat1	signal transducer and activator of transcriptio	Mm.277406
20846	Stat1	signal transducer and activator of transcriptio	Mm.277406
20846	Stat1	signal transducer and activator of transcriptio	Mm.277406
20846	Stat1	signal transducer and activator of transcriptio	Mm.277406
20846	Stat1	signal transducer and activator of transcriptio	Mm.277406
20846	Stat1	signal transducer and activator of transcriptio	Mm.277406
20846	Stat1	signal transducer and activator of transcriptio	Mm.277406
20846	Stat1	signal transducer and activator of transcriptio	Mm.277406
20846	Stat1	signal transducer and activator of transcriptio	Mm.277406
20846	Stat1	signal transducer and activator of transcriptio	Mm.277406
20851	Stat5b	signal transducer and activator of transcriptio	Mm.34064
20851	Stat5b	signal transducer and activator of transcriptio	Mm.34064
20460	Stil	Scl/Tal1 interrupting locus	Mm.3988
116873	Stim2	stromal interaction molecule 2	Mm.265985
16765 /// 623112	EG623112 /// S	predicted gene, EG623112 /// stathmin 1	Mm.378957
229277	Stoml3	stomatin (Epb7.2)-like 3	Mm.222810
229277	Stoml3	stomatin (Epb7.2)-like 3	Mm.222810
20887	Sult1a1	sulfotransferase family 1A, phenol-preferring,	Mm.368982
20887	Sult1a1	sulfotransferase family 1A, phenol-preferring,	Mm.368982
71733	Susd2	sushi domain containing 2	Mm.247956
75209	Sv2c	synaptic vesicle glycoprotein 2c	Mm.441155
320558	Sycp2	synaptonemal complex protein 2	Mm.70781
20965	Syn2	synapsin II	Mm.441431
20965	Syn2	synapsin II	Mm.441431
20965	Syn2	synapsin II	Mm.441431
20965	Syn2	synapsin II	Mm.441431
20965	Syn2	synapsin II	Mm.441431
20965	Syn2	synapsin II	Mm.441431
20979	Syt1	synaptotagmin I	Mm.289702

20979	Syt1	synaptotagmin I	Mm.289702
20979	Syt1	synaptotagmin I	Mm.289702
54525	Syt7	synaptotagmin VII	Mm.182654
54525	Syt7	synaptotagmin VII	Mm.182654
54525	Syt7	synaptotagmin VII	Mm.182654
27359	Sytl4	synaptotagmin-like 4	Mm.38674
21334	Tac2	tachykinin 2	Mm.2374
56720	Tdo2	tryptophan 2,3-dioxygenase	Mm.258622
56720	Tdo2	tryptophan 2,3-dioxygenase	Mm.258622
100121	Tdrd7	Tudor domain containing 7 (Tdrd7), mRNA	Mm.275413
100121	Tdrd7	tudor domain containing 7	Mm.275413
21810	Tgfbi	transforming growth factor, beta induced	Mm.14455
21810	Tgfbi	transforming growth factor, beta induced	Mm.14455
21810	Tgfbi	transforming growth factor, beta induced	Mm.14455
21810	Tgfbi	transforming growth factor, beta induced	Mm.14455
21835	Thrsp	thyroid hormone responsive SPOT14 homolog	Mm.28585
21835	Thrsp	thyroid hormone responsive SPOT14 homolog	Mm.28585
99929	Tiparp	TCDD-inducible poly(ADP-ribose) polymerase	Mm.246398
99929	Tiparp	TCDD-inducible poly(ADP-ribose) polymerase	Mm.246398
17112	Tm4sf1	transmembrane 4 superfamily member 1	Mm.856
17112	Tm4sf1	transmembrane 4 superfamily member 1	Mm.856
17112	Tm4sf1	transmembrane 4 superfamily member 1	Mm.856
17112	Tm4sf1	transmembrane 4 superfamily member 1	Mm.856
319880	Tmcc3	transmembrane and coiled coil domains 3	Mm.23047
319880	Tmcc3	transmembrane and coiled coil domains 3	Mm.23047
319880	Tmcc3	transmembrane and coiled coil domains 3	Mm.23047
319880	Tmcc3	transmembrane and coiled coil domains 3	Mm.23047
319880	Tmcc3	transmembrane and coiled coil domains 3	Mm.23047
319880	Tmcc3	transmembrane and coiled coil domains 3	Mm.23047
319880	Tmcc3	transmembrane and coiled coil domains 3	Mm.23047
319880	Tmcc3	transmembrane and coiled coil domains 3	Mm.23047
319880	Tmcc3	transmembrane and coiled coil domains 3	Mm.23047
319880	Tmcc3	transmembrane and coiled coil domains 3	Mm.23047
230157	Tmeff1	transmembrane protein with EGF-like and two	Mm.422686
230157	Tmeff1	transmembrane protein with EGF-like and two	Mm.422686
66910	Tmem107	transmembrane protein 107	Mm.33964
66910	Tmem107	transmembrane protein 107	Mm.33964
66910	Tmem107	transmembrane protein 107	Mm.33964
66910	Tmem107	transmembrane protein 107	Mm.33964
98170	Tmem132a	transmembrane protein 132A	Mm.477984
432591	OTTMUSG000	predicted gene, OTTMUSG00000002042	Mm.370372
21927	Tnfaip1	tumor necrosis factor, alpha-induced protein 1	tumor necrosis factor, alpha-induced protein 1 (endothelial)
21927	Tnfaip1	tumor necrosis factor, alpha-induced protein 1	tumor necrosis factor, alpha-induced protein 1 (endothelial)
21927	Tnfaip1	tumor necrosis factor, alpha-induced protein 1	tumor necrosis factor, alpha-induced protein 1 (endothelial)
21927	Tnfaip1	tumor necrosis factor, alpha-induced protein 1	tumor necrosis factor, alpha-induced protein 1 (endothelial)
21924	Tnnc1	troponin C, cardiac/slow skeletal	Mm.439921
27223	Trp53bp1	transformation related protein 53 binding prote	Mm.215389
27223	Trp53bp1	transformation related protein 53 binding prote	Mm.215389
27223	Trp53bp1	transformation related protein 53 binding prote	Mm.215389
68729	Trim37	tripartite motif-containing 37	Mm.17436
68729	Trim37	tripartite motif-containing 37	Mm.17436
69716	Trip13	thyroid hormone receptor interactor 13	Mm.275095
56191	Tro	trophinin	Mm.3597
56191	Tro	trophinin	Mm.3597
56191	Tro	trophinin	Mm.3597
56191	Tro	trophinin	Mm.3597
70423	Tspan15	tetraspanin 15	Mm.416781
241556	Tspan18	tetraspanin 18	Mm.274159
241556	Tspan18	tetraspanin 18	Mm.274159
74044	Ttf2	transcription termination factor, RNA polymera	Mm.270961
74044	Ttf2	transcription termination factor, RNA polymera	Mm.270961
27613 /// 66582	Tubb2c /// Tubt	tubulin, beta 2C /// tubulin, beta 2c, pseudoge	Mm.227260
27613 /// 66582	Tubb2c /// Tubt	tubulin, beta 2C /// tubulin, beta 2c, pseudoge	Mm.227260
227613	Tubb2c	tubulin, beta 2C	Mm.227260
227613	Tubb2c	tubulin, beta 2C	Mm.227260
27613 /// 66582	Tubb2c /// Tubt	tubulin, beta 2C /// tubulin, beta 2c, pseudoge	Mm.227260
27613 /// 66582	Tubb2c /// Tubt	tubulin, beta 2C /// tubulin, beta 2c, pseudoge	Mm.227260
27613 /// 66582	Tubb2c /// Tubt	tubulin, beta 2C /// tubulin, beta 2c, pseudoge	Mm.227260
27613 /// 66582	Tubb2c /// Tubt	tubulin, beta 2C /// tubulin, beta 2c, pseudoge	Mm.227260
56734	Tulp2	tubby-like protein 2	Mm.439742
22178	Tyrp1	tyrosinase-related protein 1	Mm.30438
22178	Tyrp1	tyrosinase-related protein 1	Mm.30438
22178	Tyrp1	tyrosinase-related protein 1	Mm.30438
50995	Uba2	ubiquitin-like modifier activating enzyme 2	Mm.27560
50995	Uba2	ubiquitin-like modifier activating enzyme 2	Mm.27560
50995	Uba2	ubiquitin-like modifier activating enzyme 2	Mm.27560
22235	Ugdh	UDP-glucose dehydrogenase	Mm.344831
52020	Umodl1	uromodulin-like 1	Mm.25923
107449	Unc5b	unc-5 homolog B (C. elegans)	Mm.290433
107449	Unc5b	unc-5 homolog B (C. elegans)	Mm.290433
107449	Unc5b	unc-5 homolog B (C. elegans)	Mm.290433
107449	Unc5b	unc-5 homolog B (C. elegans)	Mm.290433
170707	Usp48	ubiquitin specific peptidase 48	Mm.218478
170707	Usp48	ubiquitin specific peptidase 48	Mm.218478
170707	Usp48	ubiquitin specific peptidase 48	Mm.218478
170707	Usp48	ubiquitin specific peptidase 48	Mm.218478
98910	Usp6nl	USP6 N-terminal like	Mm.40673
98910	Usp6nl	USP6 N-terminal like	Mm.40673
73569	Vgll3	vestigial like 3 (Drosophila)	Mm.25670
73569	Vgll3	vestigial like 3 (Drosophila)	Mm.25670
73569	Vgll3	vestigial like 3 (Drosophila)	Mm.25670
73569	Vgll3	vestigial like 3 (Drosophila)	Mm.25670
22361	Vnn1	vanin 1	Mm.27154
22361	Vnn1	vanin 1	Mm.27154
83767	Wasf1	WASP family 1	Mm.41353
218973	Wdhd1	WD repeat and HMG-box DNA binding protei	Mm.265615
218973	Wdhd1	WD repeat and HMG-box DNA binding protei	Mm.265615
218973	Wdhd1	WD repeat and HMG-box DNA binding protei	Mm.265615
74682	Wdr35	WD repeat domain 35	Mm.87389
140858	Wdr5	WD repeat domain 5	Mm.28265
140858	Wdr5	WD repeat domain 5	Mm.28265
210544	Wdr67	WD repeat domain 67	
210544	Wdr67	WD repeat domain 67	
22393	Wfs1	Wolfram syndrome 1 homolog (human)	Mm.20916
211652	Wwc1	WW, C2 and coiled-coil domain containing 1	Mm.31267
211652	Wwc1	WW, C2 and coiled-coil domain containing 1	Mm.31267
211652	Wwc1	WW, C2 and coiled-coil domain containing 1,	Mm.31267
22601	Yap1	yes-associated protein 1	Mm.221992
22601	Yap1	yes-associated protein 1	Mm.221992
77519	5730601F06Rik	RIKEN cDNA 5730601F06 gene	Mm.428682
218820	Zfp503	zinc finger protein 503	Mm.292401
68014	Zwilch	Zwilch, kinetochore associated, homolog (Dro	Mm.335237
