# Supplementary material for: Meta-Analysis of Autoimmune Regulator-Regulated Genes in Human and Murine Models: A Novel Human Model Provides Insights on the Role of Autoimmune Regulator in Regulating STAT1 and STAT1-Regulated Genes
Source: Front Immunol. 2018 Jun 28;9:1380. doi: 10.3389/fimmu.2018.01380 (PMC6031710; doi:10.3389/fimmu.2018.01380)
Supplement: Table S3 — List of gene-specific primer sequences used for QPCR assays, with PrimerBank (49) reference number where appropriate. [file table_3.PDF]

Supplementary Table 3. QPCR Primer Sequences

Gene	Forward primer (5'-3')	Reverse primer (5'-3')	Primer bank ID
β-Actin	TCCCCCAACTTGAGATGTATGAAG (0.1μM)	AACTGGTCTCAAGTCAGTGTACAGG (0.1μM)	
AIRE	CATCCAGCAGGTGTTTGAGTCA (0.3μM)	CGAACTTGCTGGGAGTGTAGAA (0.6μM)	
CAMK2B	ACCGTCATCCATAACCCAGTG (0.3μM)	CTGAACCCCTTCTCACAGAGT (0.3μM)	
CAV2	AAGACCTGCCTAATGGTTCTGC (0.3μM)	CTCGTACACAATGGAGCAATGAT (0.3μM)	332164665c1
CCBP2	CTTGCTCCGTTACGTGCCT (0.3μM)	GAAACTCCCGAAGACCCAATG (0.3μM)	156523973c1
CCL3	AGTTCTCTGCATCACTTGCTG (0.3μM)	CGGCTTCGCTTGGTTAGGAA (0.3μM)	4506843a1
CCL5	CTCATTGCTACTGCCCTCTGCGCTCCTGC (0.3μM)	GCTCATCTCCAAAGAGTTGATGTACTC (0.3μM)	
CDKN1C	AGCTGCACTCGGGGATTT (0.3μM)	AAGAAATCGGAGATCAGAGGC (0.3μM);	
CEACAM1	AAAGCGACCCCATCATGCT (0.3μM)	AGGGCCACTACTCCAATCACA (0.3μM)	
COL14A1	ATGCCAGACCAGAATTACACAG (0.3μM)	ACCATCGACCAGGATTACAATGT (0.3μM)	55743095c1
DPY19L1	GAAAGGGAGATGGCTTTTCGC (0.3μM)	AATGAGGGTGCTTCCACAATAG (0.3μM)	148839334c1
DPYD	GGCGGACATCGAGAGTATCCT (0.3μM)	TTCTTGGCCGAAGTGGAACAC (0.3μM)	119943097c1
DUSP4	TGTGGAGATCCTTCCCTTCCT (0.3μM)	CAGGGCGTCCAGCATGTC (0.3μM)	
DUSP16	GCCCATGAGATGATTGGAACTC (0.3μM)	CGGCTATCAATTAGCAGCACTTT (0.3μM)	295849296c1
FNBP1L	GGATCAGTTCGACAGCTTAGAC (0.3μM)	AGGCTACACACGAGGTAAACC (0.3μM)	284172409c1
GAS7	CATCGCCAAGCAAAAAGCAGA (0.3μM)	AGCCCAGAAGTAGTCGCAGT (0.3μM)	41406079c1
IFI44	ATGGCAGTGACAACTCGTTTG (0.3μM)	TCCTGGTAACTCTCTTCTGCATA (0.3μM)	166706910c1
IFIT1	TTGATGACGATGAAATGCCTGA (0.3μM)	CAGGTCACCAGACTCCTCAC (0.3μM)	116534936c1
IFIT3	TCAGAAGTCTAGTCACTTGGGG (0.3μM)	ACACCTTCGCCCTTTCATTTC (0.3μM)	197276657c1
IL6	AAATTCGGTACATCCTCGACGG (0.3μM)	GGAAGGTTCAGGTTGTTTTCTGC (0.3μM)	
IRF7	CCCACGCTATACCATCTACCT (0.3μM)	GATGTCGTCATAGAGGCTGTTG (0.3μM)	98985817c3
KRT14	CATGAGTGTGGAAGCCGACAT (0.3μM)	GCCTCTCAGGGCATTCATCTC (0.3μM)	
KRT17	GGTGGGTGGTGAGATCAATGT (0.3μM)	CGCGGTTCAGTTCCTCTGTC (0.3μM)	21754583a1
KSR1	GGGGAGCACAAGGAGGACT (0.3μM)	GCGTGCAGGGGAATACAGG (0.3μM)	75677324c1
LEFTY2	TGGACCTCAGGGACTATGGAG-3' (0.3μM)	CCGAGGCGATACACTGTCG (0.3μM)	289063467c1
MAGEB2	CAGCCAGGGGTGAATTCTCAG (0.3μM)	TTCTCACGGGCACGGAGCTTA (0.3μM)	
OAS1	TGTCCAAGGTGGTAAAGGGTG (0.3μM)	CCGGCGATTTAACTGATCCTG (0.3μM)	74229012c1
OAS2	AGGTGGCTCCTATGGACGG (0.3μM)	TTTATCGAGGATGTCACGTTGG (0.3μM)	74229020c2
OAS3	GCTTCAAGAGCTATGTGGACC (0.3μM)	GGAAACGTGAGTCTCAGACCA (0.3μM)	45007006c2
NIPA1	CGCCCTTGGAGTACCGTTC (0.3μM)	GAGTGGATAATCAGCACGACG (0.3μM)	214010178c1
PIP4K2A	AAGAAGAAGCACTTCGTAGCG (0.3μM)	ATGGCTCAGTTCATTGATCGAG (0.3μM)	156416001c1
PRKCA	GTCCACAAGAGGTGCCATGAA (0.3μM)	AAGGTGGGGCTTCCGTAAGT (0.3μM)	47157319c1
PSD3	AACACGGCTAGAAGCTCATTC (0.3μM)	TCCAGCGTCATTCCTGTAAAATC (0.3μM)	117606358c1
RALGPS1	CAAGAGCTATGATGCCGTTGT (0.3μM)	GGCAAGACTGTGTTTCTCCTTC (0.3μM)	299758449c1
RAP1A	CGTGAGTACAAGCTAGTGGTCC (0.3μM)	CCAGGATTTCGAGCATACACTG (0.3μM)	58331201c1
REPS2	CTGACCTGTTTCGGGCATC (0.3μM)	GTTCCCAAATCGTATCTCACCAT (0.3μM)	125625325c1
RHOBTB1	ATGGACGCTGACATGGACTAC (0.3μM)	ATCCCGAGAACGCTCCAAGA (0.3μM)	334358908c1
SEPT 9	TTCGGCTACGTGGGGATTG (0.3μM)	CTGCCCGACCACCATGATG (0.3μM)	164698495c1
SNX10	CACTTTTGCTTTCAGATAGCAGC (0.3μM)	ACACACGCCTCAATGTCTTCT (0.3μM)	315360663c1
STAT1	GCAGGTTCACCAGCTTTATGA (0.3μM)	TGAAGATTACGCTTGCTTTTCCT (0.3μM)	189458859c1
TCEA1	GTCAGTTAATGCTATTCGCAAGC (0.3μM)	GCGATGTAATTGCAGGTTCTTTC (0.3μM)	
TXNIP	GGTCTTTAACGACCCTGAAAAGG (0.3μM)	ACACGAGTAACTTCACACACCT (0.3μM)	171184420c1
TULP2	AGCGCCTTTTAGGTGACAGAG (0.3μM)	TCCACGGAGACTTCCTCCAAT (0.3μM)	238859544c1
VCL	CTCGTCCGGGTTGGAAAAGAG (0.3μM)	AGTAAGGGTCTGACTGAAGCAT (0.3μM)	50593538c1
